# Design, Synthesis, and Antifungal/Anti-Oomycete Activities of Novel 1,2,4-Triazole Derivatives Containing Carboxamide Fragments

**DOI:** 10.3390/jof10020160

**Published:** 2024-02-19

**Authors:** Jiali Wang, Haoran Shi, Aidang Lu

**Affiliations:** School of Chemical Engineering and Technology, Hebei University of Technology, Tianjin 300401, China; galizhu999@163.com (J.W.); shr15130128422@163.com (H.S.)

**Keywords:** 1,2,4-triazole derivatives, carboxamide fragments, fungicidal activity, *Physalospora piricola*, *Phytophthora capsici*, molecular docking

## Abstract

Plant diseases caused by pathogenic fungi or oomycetes seriously affect crop growth and the quality and yield of products. A series of novel 1,2,4-triazole derivatives containing carboxamide fragments based on amide fragments widely used in fungicides and the commercialized mefentrifluconazole were designed and synthesized. Their antifungal activities were evaluated against seven kinds of phytopathogenic fungi/oomycete. Results showed that most compounds had similar or better antifungal activities compared to mefentrifluconazole’s inhibitory activity against *Physalospora piricola*, especially compound **6h** (92%), which possessed outstanding activity. Compound **6h** (EC_50_ = 13.095 μg/mL) showed a better effect than that of mefentrifluconazole (EC_50_ = 39.516 μg/mL). Compound **5j** (90%) displayed outstanding anti-oomycete activity against *Phytophthora capsici*, with an EC_50_ value of 17.362 μg/mL, far superior to that of mefentrifluconazole (EC_50_ = 75.433 μg/mL). The result of molecular docking showed that compounds **5j** and **6h** possessed a stronger affinity for 14α-demethylase (CYP51). This study provides a new approach to expanding the fungicidal spectrum of 1,2,4-triazole derivatives.

## 1. Introduction

Phytopathogenic fungi are responsible for about two-thirds of infectious plant diseases and pose a great threat to crop production [1,2,3]. They not only significantly affect the production and quality of crops but also endangers the health of humans and animals through the creation and enrichment of toxins [4,5]. In particular, *Physalospora piricola* (*P. piricola*), which infects apple branches, fruit, and leaves, has a serious impact on the growth and yield of fruit trees and is the main causal agent of apple ring rot [6]. It is widely distributed in most apple-growing areas in the country but is most severe in eastern China (Liaoning, Shandong, Henan, and Hebei provinces), where summer temperatures and rainfall are high. The earliest record of a severe loss caused by this disease in China was in Chengdu of Sichuan Province in 1942, when the disease caused approximately 20% fruit loss of Yuxia (Mapi) apples before harvest and another 79% loss in storage [7]. Since the 1980s, the importance of apple ring rot in eastern China has increased with the widespread planting of the cultivar Fuji. The emergence of pathogenic fungi like *P. piricola*, showing resistance to current market fungicides, underscores the need for new compounds [8]. These new antifungals are crucial not only because they can overcome the resistance developed by pathogens but also to expand the arsenal of available treatments. Therefore, there is an urgent and continuous need to seek novel and effective approaches to control pathogenic fungi and mitigate losses caused by *P. piricola*. Diversified modification from novel leading compounds is an important and widely applied strategy in the continuous innovation and development of chemical fungicides [9,10,11]. Thus, the design and synthesis of new active molecules is one of the most important recent milestones in fungicide research.

**Figure 1:**
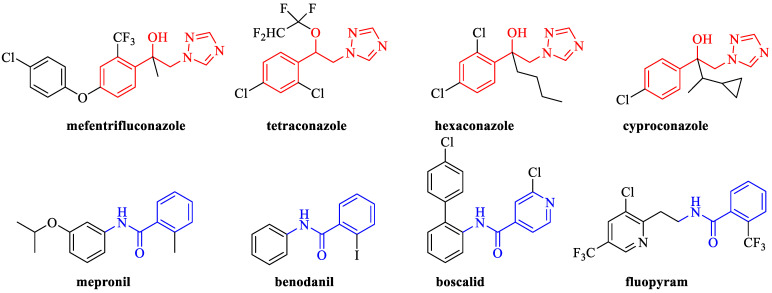
Some commercialized triazole fungicides and SDHI fungicides contain amide fragments.

Triazole fungicides mainly belong to sterol demethylation inhibitor (DMI) fungicides, which occupy an important position in the field of fungicides, such as mefentrifluconazole, tetraconazole, hexaconzole, cyproconaole, etc. (Figure 1) [12]. Mefentrifluconazole, [(2*RS*)-2-(4-(4-chlorophenoxy)-α,α,α-trifluoro-o-tolyl)-1-(1*H*-1,2,4-triazol-1-yl)propan-2-ol], is the first isopropanol triazole broad-spectrum fungicide developed by the company Badische Anilin-und-Soda-Fabrik (BASF) and it has become widely used to control a range of fungal crop diseases [13]. Similar to traditional triazole fungicides, the nitrogen atom on its heterocyclic ring has a lone pair of electrons, which can be combined with the iron atom in the heme-iron active center of 14α-demethylase (CYP51) in pathogenic fungi by ligand bonding to form an iron porphyrin-centered ligand complex, resulting in the reduction in the combination of oxygen atoms with CYP51 and the inhibition of the catalytic capacity of CYP51 oxidation, which results in the blocking of the 14α-demethylation reaction, thus interfering with the synthesis of fungal ergosterol [14,15]. On the one hand, ergosterol deficiency can lead to abnormal cell membrane structure and function, resulting in changes in membrane bonding and enzyme activity, making fungal cells vulnerable to damage and even causing dysfunction in the activation of ergosterol hormones, which affects cell growth and proliferation [16,17]; on the other hand, the blockage of the 14α-demethylation reaction can lead to the accumulation of large quantities of 24-methylenedihydrolanosterol, which inhibits the flow of fungal cell membranes [18]. In brief, mefentrifluconazole impedes the biosynthesis of ergosterol in the cell membrane of pathogens, causing changes in its biological structure, inhibiting cell growth, and finally achieving the effects of being bacteriostatic and bactericidal [19,20,21]. Unlike the existing triazole fungicides, the unique isopropanol moiety of mefentrifluconazole can be easily transformed into a “hook” that binds tightly with the target protein, resulting in potent fungicidal activity with little cross-resistance [19,22,23,24]. Mefentrifluconazole can be used to control many fungal diseases in more than 60 species of crop plants, such as potatoes, pome fruits, and soybeans, and has been registered in many countries [20]. Among them, mefentrifluconazole has been registered for the control of apple brown spot (*Marssonina mali*) in China, yet no studies have been conducted to determine its inhibitory activity against *P. piricola* and it is less effective in inhibiting oomycetes.

Studies have shown that mefentrifluconazole can also be used in combination with succinate dehydrogenase inhibitor (SDHI) fungicides such as florylpicoxamid, fluxapyroxad, boscalid, and bixafen, thereby delaying the development of fungicide resistance. SDHI fungicides disrupt the mitochondrial respiratory chain by inhibiting SDH, which leads to fungal death [25,26]. Their mechanisms of action suggest that these structurally dissimilar substances possess the common pharmacophore carboxamide, which consists of three parts, namely a polar moiety, amide bond, and hydrophobic tail [27,28,29]. Among them, amide bonds are the core feature of SDHIs and the introduction of structurally diverse benzene rings flanking the amide bond enables the compounds to exhibit good broad-spectrum activity [30]. It is desired to enhance the bactericidal activity of triazole derivatives by introducing active groups to give a broader spectrum of bioactivity.

**Figure 2:**
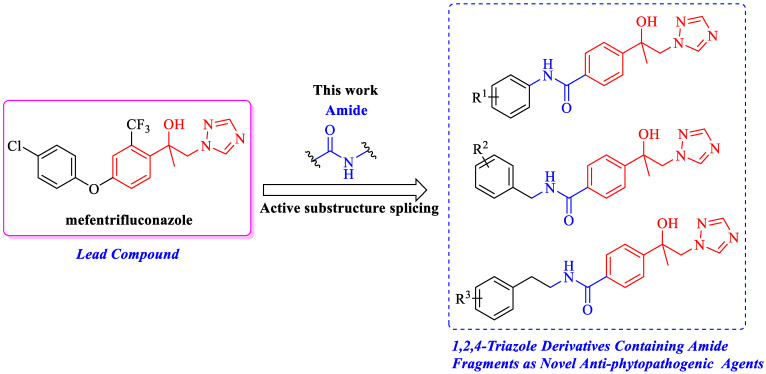
Design of 1,2,4-triazole derivatives containing carboxamide fragments.

In this study, the commercial fungicide mefentrifluconazole was used as the lead compound, retained its isopropanol triazole part, and then introduced amide fragments by using active substructure splicing. A total of 34 novel 1,2,4-triazole derivatives containing carboxamide fragments were designed and synthesized (Figure 2). Their fungicidal activities against seven kinds of phytopathogenic fungi/oomycete at 50 μg/mL were evaluated and the mechanism of action of highly effective compounds **5j** and **6h** were preliminarily evaluated.

## 2. Materials and Methods

### 2.1. Chemicals and Instruments

All reagents used were analytical reagent (AR) grade or chemically pure (CR), which were purchased from commercial sources (Tianjin Guangda Chemical Reagents Ltd., Tianjin, China). The melting point of the target compounds were measured on an X-4 binocular microscope (Beijing Zhongke Instrument Co., Ltd., Beijing, China). Nuclear magnetic resonance (NMR) spectra were acquired with a 400 MHz (100 MHz for ^13^C) instrument (Bruker, Billerica, MA, USA) at room temperature. Chemical shifts were measured relative to residual solvent peaks of CDCl_3_ (^1^H: *δ* = 7.26 ppm; ^13^C: *δ* = 77.0 ppm) and DMSO-*d*_6_ (^1^H: *δ* = 2.5 and 3.3 ppm; ^13^C: *δ* = 39.9 ppm) as internal standards. The following abbreviations are used to designate chemical shift multiplicities: s = singlet, d = doublet, dd = doublet of doublets, t = triplet, m = multiplet, and brs = broad singlet. High-resolution mass spectrometry (HRMS) data were recorded with a quadrupol Fourier transform-electrospray ionization (QFT-ESI) instrument (Varian, Palo Alto, CA, USA).

### 2.2. Synthetic Procedures

#### 2.2.1. Synthesis of Compounds **5a**–**5n**, **6a**–**6m**, and **7a**–**7g**

To a stirred solution of NaH (60% in mineral oil, 1.840 g, 46.0 mmol) in dry tetrahydrofuran (THF) (30 mL), trimethylsulfonium iodide (Me_3_S^+^I^−^) (8.163 g, 40.0 mmol) in dry dimethyl sulfoxide (DMSO) (50 mL) was added at room temperature under nitrogen. The reaction mixture was left stirring for 1 h at room temperature. A solution of compound **1** (3.564 g, 20.0 mmol) in dry DMSO (20 mL) was added dropwise and the reaction mixture was left stirring for 14 h. The reaction mixture was quenched with a saturated solution of ammonium chloride, followed by extractions with ethyl acetate (EtOAc) (3 × 20 mL). The combined organic layers were dried and the solvent was evaporated. The crude product was purified by column chromatography to obtain compound **2**. Light yellow solid, 75% yield, m.p. 46–48 °C; ^1^H NMR (400 MHz, DMSO-*d*_6_) *δ* 7.93 (d, *J* = 8.1 Hz, 2H), 7.49 (d, *J* = 8.0 Hz, 2H), 3.84 (s, 3H), 3.04 (d, *J* = 6.0 Hz, 1H), 2.79 (d, *J* = 5.2 Hz, 1H), 1.67 (s, 3H); ^13^C NMR (100 MHz, DMSO-*d*_6_) *δ* 166.5, 147.0, 129.7, 129.2, 126.2, 57.0, 56.7, 52.6, 21.5.

Compound **2** (1.922 g, 10.0 mmol), 1,2,4-Triazole (2.763 g, 40.0 mmol), and NaOH (0.800 g, 20.0 mmol.) were added to *N*,*N*-dimethylformamide (DMF) (50 mL) and stirred at 110 °C for 4 h, cooled to room temperature, saturated NH_4_Cl solution was added, and the aqueous phase was extracted with EtOAc (3 × 15 mL). The combined organic phases were washed with saturated brine, dried over anhydrous magnesium sulfate, filtered with suction, spin-dried, and recrystallized from dichloromethane ether to obtain compound **3**. White solid, 80% yield, m.p. 98–100 °C; ^1^H NMR (400 MHz, DMSO-*d*_6_) *δ* 8.23 (s, 1H), 7.89 (d, *J* = 8.2 Hz, 2H), 7.83 (s, 1H), 7.57 (d, *J* = 8.0 Hz, 2H), 5.73 (s, 1H), 4.41 (s, 2H), 3.84 (s, 3H), 1.46 (s, 3H); ^13^C NMR (100 MHz, DMSO-*d*_6_) *δ* 166.6, 151.8, 150.9, 145.4, 129.3, 128.6, 126.2, 73.2, 59.7, 52.6, 27.5.

To a stirred solution of **3** (1.306 g, 5.0 mmol) in THF (30 mL), NaOH (0.600 g, 15.0 mmol.) in H_2_O (30 mL) was added and the reaction mixture was stirred at room temperature for 6 h. After completion of the reaction, the solvent was removed in vacuo. Then, dilute HCl (10 mL, 1 M) was added dropwise to the reaction mixture to adjust the pH to 3–5, the solid was filtered and dried, and compound **4** was obtained. White solid, 74% yield, m.p. 199–202 °C; ^1^H NMR (400 MHz, DMSO-*d*_6_) *δ* 12.86 (s, 1H), 8.23 (s, 1H), 7.90–7.82 (m, 3H), 7.55 (d, *J* = 8.2 Hz, 2H), 5.70 (s, 1H), 4.41 (s, 2H), 1.45 (s, 3H); ^13^C NMR (100 MHz, DMSO-*d*_6_) *δ* 167.7, 151.3, 150.8, 145.4, 129.8, 129.5, 126.0, 73.2, 59.7, 27.6.

To a stirred solution of **4** (0.247 g, 1.0 mmol) in CH_2_Cl_2_ (3 mL), Et_3_N (0.202 g, 2.0 mmol), 1-hydroxybenzotriazole (HOBt) (0.203 g, 1.5 mmol), and 1-(3-dimethylaminopropyl)-3-ethylcarbodiimide hydrochloride (EDCI) (0.288 g, 1.5 mmol) were added and the reaction mixture was stirred at room temperature for 30 min. Subsequently, various substituted aromatic amines (1.5 mmol) were added and the resulting solution was stirred at room temperature for 12 h. The reaction mixture was diluted with H_2_O (10 mL), followed by extractions with EtOAc (3 × 15 mL). The combined organic phase was dried with anhydrous sodium sulfate and the solvent was evaporated. The crude product was purified by column chromatography to obtain compounds **5a**–**5n**, **6a**–**6m**, and **7a**–**7g**.

4-(2-Hydroxy-1-(1*H*-1,2,4-triazol-1-yl)propan-2-yl)-*N*-phenylbenzamide (**5a**). White solid, 85% yield, m.p. 181–183 °C; ^1^H NMR (400 MHz, DMSO-*d*_6_) *δ* 10.21 (s, 1H), 8.27 (s, 1H), 7.93–7.84 (m, 3H), 7.77 (d, *J* = 7.9 Hz, 2H), 7.59 (d, *J* = 8.0 Hz, 2H), 7.35 (t, *J* = 8.1 Hz, 2H), 7.09 (t, *J* = 7.7 Hz, 1H), 5.71 (s, 1H), 4.45 (s, 2H), 1.47 (s, 3H); ^13^C NMR (100 MHz, DMSO-*d*_6_) *δ* 165.8, 150.9, 150.0, 145.4, 139.7, 133.9, 129.1, 127.8, 125.8, 124.1, 120.8, 73.2, 59.8, 27.8; HR-MS (ESI): calcd for C_18_H_18_N_4_O_2_ [M+H]^+^ 323.1503, found (ESI^+^) 323.1509.

4-(2-Hydroxy-1-(1*H*-1,2,4-triazol-1-yl)propan-2-yl)-*N*-(o-tolyl)benzamide (**5b**). White solid, 84% yield, m.p. 145–147 °C; ^1^H NMR (400 MHz, DMSO-*d*_6_) *δ* 9.83 (s, 1H), 8.27 (s, 1H), 7.94–7.84 (m, 3H), 7.58 (d, *J* = 8.1 Hz, 2H), 7.32 (d, *J* = 7.8 Hz, 1H), 7.27 (d, *J* = 7.5 Hz, 1H), 7.19 (m, 2H), 5.70 (s, 1H), 4.44 (s, 2H), 2.23 (s, 3H), 1.47 (s, 3H); ^13^C NMR (100 MHz, DMSO-*d*_6_) *δ* 165.6, 151.0, 149.9, 145.4, 137.0, 134.3, 133.4, 130.8, 127.8, 127.1, 126.5, 125.8, 73.2, 59.8, 27.8, 18.4; HR-MS (ESI): calcd for C_19_H_20_N_4_O_2_ [M+H]^+^ 337.1660, found (ESI^+^) 337.1651.

4-(2-Hydroxy-1-(1*H*-1,2,4-triazol-1-yl)propan-2-yl)-*N*-(p-tolyl)benzamide (**5c**). White solid, 79% yield, m.p. 167–169 °C; ^1^H NMR (400 MHz, DMSO-*d*_6_) *δ* 10.12 (s, 1H), 8.26 (s, 1H), 7.91–7.84 (m, 3H), 7.65 (d, *J* = 8.0 Hz, 2H), 7.58 (d, *J* = 8.1 Hz, 2H), 7.15 (d, *J* = 8.0 Hz, 2H), 5.70 (s, 1H), 4.44 (s, 2H), 2.28 (s, 3H), 1.46 (s, 3H); ^13^C NMR (100 MHz, DMSO-*d*_6_) δ 165.6, 151.0, 149.9, 145.4, 137.2, 134.0, 133.1, 129.5, 127.8, 125.7, 120.9, 73.2, 59.8, 27.7, 21.0; HR-MS (ESI): calcd for C_19_H_20_N_4_O_2_ [M+H]^+^ 337.1660, found (ESI^+^) 337.1653.

4-(2-Hydroxy-1-(1*H*-1,2,4-triazol-1-yl)propan-2-yl)-*N*-(2-methoxyphenyl)benzamide (**5d**). Brown solid, 80% yield, m.p. 156–158 °C; ^1^H NMR (400 MHz, DMSO-*d*_6_) *δ* 9.38 (s, 1H), 8.26 (s, 1H), 7.91–7.86 (t, 3H), 7.77 (d, *J* = 7.8 Hz, 1H), 7.57 (d, *J* = 8.4 Hz, 2H), 7.18 (t, *J* = 7.8 Hz, 1H), 7.09 (d, *J* = 8.2 Hz, 1H), 6.97 (t, *J* = 7.6 Hz, 1H), 5.71 (s, 1H), 4.44 (s, 2H), 3.83 (s, 3H), 1.47 (s, 3H); ^13^C NMR (100 MHz, DMSO-*d*_6_) *δ* 167.7, 165.3, 152.0, 151.0, 150.0, 145.4, 133.4, 129.5, 127.6, 125.9, 124.8, 120.7, 111.9, 73.2, 59.8, 56.2, 27.7; HR-MS (ESI): calcd for C_19_H_20_N_4_O_3_ [M+H]^+^ 353.1609, found (ESI^+^) 353.1617.

4-(2-Hydroxy-1-(1*H*-1,2,4-triazol-1-yl)propan-2-yl)-*N*-(3-methoxyphenyl)benzamide (**5e**). Yellow crystals, 67% yield, m.p. 116–118 °C; ^1^H NMR (400 MHz, DMSO-*d*_6_) *δ* 10.18 (s, 1H), 8.27 (s, 1H), 7.91–7.83 (m, 3H), 7.59 (d, *J* = 8.0 Hz, 2H), 7.48 (s, 1H), 7.37 (d, *J* = 8.1 Hz, 1H), 7.24 (t, *J* = 8.1 Hz, 1H), 6.68 (d, *J* = 8.2 Hz, 1H), 5.71 (s, 1H), 4.44 (s, 2H), 3.75 (s, 3H), 1.47 (s, 3H); ^13^C NMR (100 MHz, DMSO-*d*_6_) *δ* 165.9, 159.9, 150.9, 150.0, 145.4, 140.9, 133.9, 129.9, 127.8, 125.8, 113.0, 109.6, 106.5, 73.2, 59.8, 55.5, 27.7; HR-MS (ESI): calcd for C_19_H_20_N_4_O_3_ [M+H]^+^ 353.1609, found (ESI^+^) 353.1619.

4-(2-Hydroxy-1-(1*H*-1,2,4-triazol-1-yl)propan-2-yl)-*N*-(4-methoxyphenyl)benzamide (**5f**). White solid, 77% yield, m.p. 175–178 °C; ^1^H NMR (400 MHz, DMSO-*d*_6_) *δ* 10.09 (s, 1H), 8.26 (s, 1H), 7.90–7.84 (m, 3H), 7.67 (d, *J* = 8.6 Hz, 2H), 7.57 (d, *J* = 8.1 Hz, 2H), 6.92 (d, *J* = 8.6 Hz, 2H), 5.70 (s, 1H), 4.44 (s, 2H), 3.74 (s, 3H), 1.47 (s, 3H); ^13^C NMR (100 MHz, DMSO-*d*_6_) *δ* 165.4, 156.0, 151.0, 149.8, 145.4, 133.9, 132.7, 127.7, 125.7, 122.5, 114.2, 73.2, 59.8, 55.7, 27.7; HR-MS (ESI): calcd for C_19_H_20_N_4_O_3_ [M+H]^+^ 353.1609, found (ESI^+^) 353.1615.

4-(2-Hydroxy-1-(1H-1,2,4-triazol-1-yl)propan-2-yl)-*N*-(3-(trifluoromethyl)phenyl)benzamide (**5g**). White solid, 51% yield, m.p. 65–67 °C; 1H NMR (400 MHz, DMSO-d6) δ 10.52 (s, 1H), 8.26 (d, *J* = 6.0 Hz, 2H), 8.06 (d, *J* = 8.2 Hz, 1H), 7.92 (d, *J* = 8.2 Hz, 2H), 7.85 (s, 1H), 7.61 (d, *J* = 8.4 Hz, 3H), 7.45 (d, *J* = 8.1 Hz, 1H), 5.73 (s, 1H), 4.45 (s, 2H), 1.48 (s, 3H); 13C NMR (100 MHz, DMSO-d6) δ 166.2, 150.9, 150.4, 145.4, 140.5, 133.3, 130.4, 129.9 (d, *J* = 31.7 Hz), 127.9, 125.9, 124.3, 123.3, 120.4, 116.8 (d, *J* = 4.3 Hz), 73.2, 59.8, 27.7; HR-MS (ESI): calcd for C19H17F3N4O2 [M+H]+ 391.1377, found (ESI+) 391.1370.

*N*-(3-Fluorophenyl)-4-(2-hydroxy-1-(1*H*-1,2,4-triazol-1-yl)propan-2-yl)benzamide (**5h**). White solid, 44% yield, m.p. 132–135 °C; ^1^H NMR (400 MHz, DMSO-*d*_6_) *δ* 10.39 (s, 1H), 8.27 (s, 1H), 7.92–7.83 (m, 3H), 7.76 (d, *J* = 12.0 Hz, 1H), 7.63–7.53 (m, 3H), 7.38 (q, *J* = 7.8 Hz, 1H), 6.92 (t, *J* = 8.8 Hz, 1H), 5.72 (s, 1H), 4.45 (s, 2H), 1.47 (s, 3H); ^13^C NMR (100 MHz, DMSO-*d*_6_) *δ* 166.1, 162.6 (d, *J* = 240.9 Hz), 150.9, 150.2, 145.4, 141.5 (d, *J* = 10.9 Hz), 133.5, 130.7 (d, *J* = 9.5 Hz), 127.9, 125.8, 116.5, 110.6 (d, *J* = 21.1 Hz), 107.4 (d, *J* = 26.2 Hz), 73.2, 59.8, 27.8; HR-MS (ESI): calcd for C_18_H_17_FN_4_O_2_ [M+H]^+^ 341.1409, found (ESI^+^) 341.1407.

*N*-(4-Fluorophenyl)-4-(2-hydroxy-1-(1*H*-1,2,4-triazol-1-yl)propan-2-yl)benzamide (**5i**). White solid, 74% yield, m.p. 154–157 °C; ^1^H NMR (400 MHz, DMSO-*d*_6_) *δ* 10.26 (s, 1H), 8.26 (s, 1H), 7.91–7.84 (m, 3H), 7.82–7.75 (m, 2H), 7.59 (d, *J* = 8.0 Hz, 2H), 7.19 (t, *J* = 8.9 Hz, 2H), 5.71 (s, 1H), 4.44 (s, 2H), 1.47 (s, 3H); ^13^C NMR (100 MHz, DMSO-*d*_6_) *δ* 165.8, 158.8 (d, *J* = 240.4 Hz), 150.9, 150.0, 145.4, 136.0, 133.7, 127.8, 125.8, 122. 7 (d, *J* = 7.8 Hz), 115.7 (d, *J* = 22.2 Hz), 73.2, 59.8, 27.7; HR-MS (ESI): calcd for C_18_H_17_FN_4_O_2_ [M+H]^+^ 341.1409, found (ESI^+^) 341.1405.

*N*-(3-Chlorophenyl)-4-(2-hydroxy-1-(1*H*-1,2,4-triazol-1-yl)propan-2-yl)benzamide (**5j**). Yellow solid, 77% yield, m.p. 115–118 °C; ^1^H NMR (400 MHz, DMSO-*d*_6_) *δ* 10.36 (s, 1H), 8.26 (s, 1H), 7.96 (s, 1H), 7.90–7.83 (m, 3H), 7.71 (d, *J* = 8.3 Hz, 1H), 7.59 (d, *J* = 8.0 Hz, 2H), 7.37 (t, *J* = 8.1 Hz, 1H), 7.15 (d, *J* = 8.1 Hz, 1H), 5.72 (s, 1H), 4.44 (s, 2H), 1.47 (s, 3H); ^13^C NMR (100 MHz, DMSO-*d*_6_) *δ* 171.0, 166.1, 151.0, 150.3, 145.4, 141.2, 133.4, 130.8, 127.9, 125.9, 123.8, 120.2, 119.1, 73.2, 59.8, 27.8; HR-MS (ESI): calcd for C_18_H_17_ClN_4_O_2_ [M+H]^+^ 357.1113, found (ESI^+^) 357.1105.

*N*-(4-Chlorophenyl)-4-(2-hydroxy-1-(1*H*-1,2,4-triazol-1-yl)propan-2-yl)benzamide (**5k**). Light yellow solid, 54% yield, m.p. 215–217 °C; ^1^H NMR (400 MHz, DMSO-*d*_6_) *δ* 10.34 (s, 1H), 8.27 (s, 1H), 7.92–7.77 (m, 5H), 7.59 (d, *J* = 8.1 Hz, 2H), 7.41 (d, *J* = 8.5 Hz, 2H), 5.72 (s, 1H), 4.44 (s, 2H), 1.47 (s, 3H); ^13^C NMR (100 MHz, DMSO-*d*_6_) *δ* 166.0, 150.9, 150.1, 145.4, 138.7, 133.6, 129.0, 127.9, 127.7, 125.8, 122.4, 73.2, 59.8, 27.8; HR-MS (ESI): calcd for C_18_H_17_ClN_4_O_2_ [M+H]^+^ 357.1113, found (ESI^+^) 357.1104.

*N*-(3-Bromophenyl)-4-(2-hydroxy-1-(1*H*-1,2,4-triazol-1-yl)propan-2-yl)benzamide (**5l**). White solid, 50% yield, m.p. 76–79 °C; ^1^H NMR (400 MHz, DMSO-*d*_6_) *δ* 10.35 (s, 1H), 8.27 (s, 1H), 8.11 (s, 1H), 7.91–7.84 (m, 3H), 7.76 (d, *J* = 7.6 Hz, 1H), 7.60 (d, *J* = 8.1 Hz, 2H), 7.31 (q, *J* = 8.8, 8.4 Hz, 2H), 5.72 (s, 1H), 4.45 (s, 2H), 1.47 (s, 3H); ^13^C NMR (100 MHz, DMSO-*d*_6_) *δ* 166.1, 150.9, 150.3, 145.4, 141.3, 133.4, 131.1, 127.9, 126.7, 125.8, 123.0, 121.9, 119.5, 73.2, 59.8, 27.8; HR-MS (ESI): calcd for C_18_H_17_BrN_4_O_2_ [M+H]^+^ 401.0608, found (ESI^+^) 401.0616.

*N*-(4-Bromophenyl)-4-(2-hydroxy-1-(1*H*-1,2,4-triazol-1-yl)propan-2-yl)benzamide (**5m**). White solid, 75% yield, m.p. 219–222 °C; ^1^H NMR (400 MHz, DMSO-*d*_6_) *δ* 10.33 (s, 1H), 8.27 (s, 1H), 7.91–7.83 (m, 3H), 7.77 (d, *J* = 8.6 Hz, 2H), 7.59 (d, *J* = 8.1 Hz, 2H), 7.53 (d, *J* = 8.6 Hz, 2H), 5.71 (s, 1H), 4.44 (s, 2H), 1.47 (s, 3H); ^13^C NMR (100 MHz, DMSO-*d*_6_) *δ* 166.0, 150.9, 150.2, 145.4, 139.1, 133.6, 131.9, 127.9, 125.8, 122.7, 115.8, 73.2, 59.8, 27.8; HR-MS (ESI): calcd for C_18_H_17_BrN_4_O_2_ [M+H]^+^ 401.0608, found (ESI^+^) 401.0614.

*N*-(3-Chloro-4-methoxyphenyl)-4-(2-hydroxy-1-(1*H*-1,2,4-triazol-1-yl)propan-2-yl)benzamide (**5n**). White solid, 71% yield, m.p. 175–177 °C; ^1^H NMR (400 MHz, DMSO-*d*_6_) *δ* 10.20 (s, 1H), 8.26 (s, 1H), 7.93 (s, 1H), 7.90–7.84 (m, 3H), 7.67 (d, *J* = 8.9 Hz, 1H), 7.58 (d, *J* = 8.0 Hz, 2H), 7.15 (d, *J* = 8.9 Hz, 1H), 5.71 (s, 1H), 4.44 (s, 2H), 3.84 (s, 3H), 1.47 (s, 3H); ^13^C NMR (100 MHz, DMSO-*d*_6_) *δ* 165.6, 151.3, 150.9, 150.0, 145.4, 133.6, 133.4, 127.8, 125.8, 122.4, 120.9, 120.7, 113.3, 73.2, 59.8, 56.7, 27.7; HR-MS (ESI): calcd for C_19_H_19_ClN_4_O_3_ [M+H]^+^ 387.1219, found (ESI^+^) 387.1213.

*N*-Benzyl-4-(2-hydroxy-1-(1*H*-1,2,4-triazol-1-yl)propan-2-yl)benzamide (**6a**). White solid, 81% yield, m.p. 131–134 °C; ^1^H NMR (400 MHz, DMSO-*d*_6_) *δ* 9.05–8.98 (m, 1H), 8.24 (s, 1H), 7.84 (d, *J* = 7.4 Hz, 3H), 7.53 (d, *J* = 8.0 Hz, 2H), 7.32 (s, 5H), 5.67 (s, 1H), 4.48 (d, *J* = 5.9 Hz, 2H), 4.41 (s, 2H), 1.45 (s, 3H); ^13^C NMR (100 MHz, DMSO-*d*_6_) *δ* 166.5, 150.9, 149.6, 145.4, 140.2, 133.2, 128.8, 127.7, 127.4, 127.2, 125.7, 73.2, 59.8, 43.1, 27.7; HR-MS (ESI): calcd for C_19_H_20_N_4_O_2_ [M+H]^+^ 337.1660, found (ESI^+^) 337.1652.

4-(2-Hydroxy-1-(1*H*-1,2,4-triazol-1-yl)propan-2-yl)-*N*-(2-methylbenzyl)benzamide (**6b**). White solid, 83% yield, m.p. 118–121 °C; ^1^H NMR (400 MHz, DMSO-*d*_6_) *δ* 8.87 (m, 1H), 8.25 (s, 1H), 7.84 (d, *J* = 5.7 Hz, 3H), 7.53 (d, *J* = 7.9 Hz, 2H), 7.23 (d, *J* = 5.8 Hz, 1H), 7.16 (s, 3H), 5.67 (s, 1H), 4.45 (d, *J* = 5.5 Hz, 2H), 4.42 (s, 2H), 2.32 (s, 3H), 1.44 (s, 3H); ^13^C NMR (100 MHz, DMSO-*d*_6_) *δ* 166.5, 150.9, 149.6, 145.4, 137.7, 136.0, 133.3, 130.4, 127.8, 127.4, 127.2, 126.2, 125.7, 73.2, 59.8, 41.1, 27.7, 19.3; HR-MS (ESI): calcd for C_20_H_22_N_4_O_2_ [M+H]^+^ 351.1816, found (ESI^+^) 351.1808.

4-(2-Hydroxy-1-(1*H*-1,2,4-triazol-1-yl)propan-2-yl)-*N*-(3-methylbenzyl)benzamide (**6c**). White solid, 85% yield, m.p. 55–57 °C; ^1^H NMR (400 MHz, DMSO-*d*_6_) *δ* 8.97 (t, *J* = 6.3 Hz, 1H), 8.24 (s, 1H), 7.83 (d, *J* = 8.0 Hz, 3H), 7.52 (d, *J* = 8.0 Hz, 2H), 7.20 (t, *J* = 7.6 Hz, 1H), 7.14–7.08 (m, 2H), 7.05 (d, *J* = 7.5 Hz, 1H), 5.66 (s, 1H), 4.42 (dd, *J* = 10.8, 5.2 Hz, 1H), 2.28 (s, 3H), 1.44 (s, 3H); ^13^C NMR (100 MHz, DMSO-*d*_6_) *δ* 166.4, 150.9, 149.6, 145.4, 140.1, 137.8, 133.3, 128.7, 128.3, 127.9, 127.4, 125.7, 124.8, 73.2, 59.8, 43.0, 27.7, 21.6; HR-MS (ESI): calcd for C_20_H_22_N_4_O_2_ [M+H]^+^ 351.1816, found (ESI^+^) 351.1821.

4-(2-Hydroxy-1-(1*H*-1,2,4-triazol-1-yl)propan-2-yl)-*N*-(4-methylbenzyl)benzamide (**6d**). White solid, 75% yield, m.p. 141–144 °C; ^1^H NMR (400 MHz, DMSO-*d*_6_) *δ* 8.96 (t, *J* = 5.1 Hz, 1H), 8.24 (s, 1H), 7.82 (d, *J* = 9.4 Hz, 3H), 7.52 (d, *J* = 7.8 Hz, 2H), 7.20 (d, *J* = 7.8 Hz, 2H), 7.12 (d, *J* = 7.4 Hz, 2H), 5.66 (s, 1H), 4.42 (m, 4H), 2.27 (s, 3H), 1.44 (s, 3H); ^13^C NMR (100 MHz, DMSO-*d*_6_) *δ* 166.4, 150.9, 149.6, 145.4, 137.2, 136.2, 133.3, 129.3, 127.7, 127.4, 125.7, 73.2, 59.8, 42.8, 27.7, 21.2; HR-MS (ESI): calcd for C_20_H_22_N_4_O_2_ [M+H]^+^ 351.1816, found (ESI^+^) 351.1809.

4-(2-Hydroxy-1-(1*H*-1,2,4-triazol-1-yl)propan-2-yl)-*N*-(4-methoxybenzyl)benzamide (**6e**). White solid, 85% yield, m.p. 115–118 °C; ^1^H NMR (400 MHz, DMSO-*d*_6_) *δ* 8.96–8.89 (m, 1H), 8.24 (s, 1H), 7.84–7.79 (m, 3H), 7.51 (d, *J* = 8.0 Hz, 2H), 7.24 (d, *J* = 8.1 Hz, 2H), 6.88 (d, *J* = 8.0 Hz, 2H), 5.66 (s, 1H), 4.40 (d, *J* = 6.3 Hz, 4H), 3.72 (s, 3H), 1.44 (s, 3H); ^13^C NMR (100 MHz, DMSO-*d*_6_) *δ* 166.4, 158.7, 150.9, 149.5, 145.3, 133.3, 132.2, 129.1, 127.4, 125.7, 114.2, 73.1, 59.8, 55.6, 42.5, 27.7; HR-MS (ESI): calcd for C_20_H_22_N_4_O_3_ [M+H]^+^ 367.1765, found (ESI^+^) 367.1761.

*N*-(2,3-Dimethoxybenzyl)-4-(2-hydroxy-1-(1*H*-1,2,4-triazol-1-yl)propan-2-yl)benzamide (**6f**). White solid, 58% yield, m.p. 50–52 °C; ^1^H NMR (400 MHz, DMSO-*d*_6_) *δ* 8.90–8.83 (m, 1H), 8.24 (s, 1H), 7.83 (d, *J* = 7.6 Hz, 3H), 7.52 (d, *J* = 8.0 Hz, 2H), 7.01 (t, *J* = 8.1 Hz, 1H), 6.94 (d, *J* = 8.1 Hz, 1H), 6.84 (d, *J* = 7.6 Hz, 1H), 5.66 (s, 1H), 4.48 (d, *J* = 5.9 Hz, 2H), 4.41 (s, 2H), 3.80 (s, 3H), 3.77 (s, 3H), 1.44 (s, 3H); ^13^C NMR (100 MHz, DMSO-*d*_6_) *δ* 166.5, 152.8, 150.9, 149.7, 146.6, 145.4, 133.4, 133.3, 127.4, 125.7, 124.3, 120.3, 112.0, 73.2, 60.5, 59.8, 56.2, 37.8, 27.7; HR-MS (ESI): calcd for C_21_H_24_N_4_O_4_ [M+H]^+^ 397.1871, found (ESI^+^) 397.1867.

4-(2-Hydroxy-1-(1*H*-1,2,4-triazol-1-yl)propan-2-yl)-*N*-(4-(trifluoromethyl)benzyl)benzamide (**6g**). White solid, 80% yield, m.p. 63–65 °C; ^1^H NMR (400 MHz, DMSO-*d*_6_) *δ* 9.17–9.07 (m, 1H), 8.25 (s, 1H), 7.84 (d, *J* = 6.0 Hz, 3H), 7.69 (d, *J* = 8.0 Hz, 2H), 7.54 (d, *J* = 8.0 Hz, 4H), 5.68 (s, 1H), 4.55 (d, *J* = 5.8 Hz, 2H), 4.42 (s, 2H), 1.45 (s, 3H); ^13^C NMR (100 MHz, DMSO-*d*_6_) *δ* 166.7, 150.9, 149.8, 145.4, 145.1, 133.0, 128.4, 128.0 (d, *J* = 31.8 Hz), 127.4, 126.2, 125.8, 125.7 (d, *J* = 4.0 Hz), 123.5, 73.2, 59.8, 42.8, 27.7; HR-MS (ESI): calcd for C_20_H_19_F_3_N_4_O_2_ [M+H]^+^ 405.1533, found (ESI^+^) 405.1528.

*N*-(2-Fluorobenzyl)-4-(2-hydroxy-1-(1*H*-1,2,4-triazol-1-yl)propan-2-yl)benzamide (**6h**). White solid, 57% yield, m.p. 165–167 °C; ^1^H NMR (400 MHz, DMSO-*d*_6_) *δ* 9.03–8.97 (m, 1H), 8.25 (s, 1H), 7.84 (d, *J* = 7.4 Hz, 3H), 7.53 (d, *J* = 8.1 Hz, 2H), 7.33 (dt, *J* = 24.6, 7.2 Hz, 2H), 7.20–7.15 (m, 2H), 5.67 (s, 1H), 4.51 (d, *J* = 5.7 Hz, 2H), 4.41 (s, 2H), 1.44 (s, 3H); ^13^C NMR (100 MHz, DMSO-*d*_6_) *δ* 166.6, 160.5 (d, *J* = 244.0 Hz), 150.9, 149.7, 145.4, 133.0, 129.9 (d, *J* = 4.4 Hz), 129.3 (d, *J* = 8.2 Hz), 127.4, 125.7, 124.8 (d, *J* = 3.4 Hz), 115.6 (d, *J* = 21.3 Hz), 73.2, 59.8, 36.9 (d, *J* = 4.7 Hz), 27.7; HR-MS (ESI): calcd for C_19_H_19_FN_4_O_2_ [M+H]^+^ 355.1565, found (ESI^+^) 355.1572.

*N*-(4-Fluorobenzyl)-4-(2-hydroxy-1-(1*H*-1,2,4-triazol-1-yl)propan-2-yl)benzamide (**6i**). White solid, 65% yield, m.p. 149–151 °C; ^1^H NMR (400 MHz, DMSO-*d*_6_) *δ* 9.05–8.99 (m, 1H), 8.24 (s, 1H), 7.83 (d, *J* = 9.3 Hz, 3H), 7.52 (d, *J* = 8.0 Hz, 2H), 7.35 (t, *J* = 7.0 Hz, 2H), 7.14 (t, *J* = 8.8 Hz, 2H), 5.67 (s, 1H), 4.45 (d, *J* = 5.7 Hz, 2H), 4.41 (s, 2H), 1.44 (s, 3H); ^13^C NMR (100 MHz, DMSO-*d*_6_) *δ* 166.5, 161.7 (d, *J* = 242.0 Hz), 150.9, 149.6, 145.4, 136.4 (d, *J* = 3.1 Hz), 133.2, 129.7 (d, *J* = 8.0 Hz), 127.4, 125.7, 115.5 (d, *J* = 21.3 Hz), 73.2, 59.8, 42.4, 27.7; HR-MS (ESI): calcd for C_19_H_19_FN_4_O_2_ [M+H]^+^ 355.1565, found (ESI^+^) 355.1569.

*N*-(2-Chlorobenzyl)-4-(2-hydroxy-1-(1*H*-1,2,4-triazol-1-yl)propan-2-yl)benzamide (**6j**). White solid, 66% yield, m.p. 150–153 °C; ^1^H NMR (400 MHz, DMSO-*d*_6_) *δ* 9.06–8.98 (m, 1H), 8.26 (s, 1H), 7.86 (d, *J* = 8.2 Hz, 3H), 7.55 (d, *J* = 8.1 Hz, 2H), 7.46 (d, *J* = 7.3 Hz, 1H), 7.32 (dd, *J* = 13.5, 7.0 Hz, 3H), 5.68 (s, 1H), 4.54 (d, *J* = 5.7 Hz, 2H), 4.42 (s, 2H), 1.45 (s, 3H); ^13^C NMR (100 MHz, DMSO-*d*_6_) *δ* 166.8, 150.9, 149.8, 145.4, 136.9, 133.0, 132.4, 129.6, 129.1, 127.7, 127.5, 125.8, 73.2, 59.8, 41.0, 27.7; HR-MS (ESI): calcd for C_19_H_19_ClN_4_O_2_ [M+H]^+^ 371.1270, found (ESI^+^) 371.1267.

*N*-(3-Chlorobenzyl)-4-(2-hydroxy-1-(1*H*-1,2,4-triazol-1-yl)propan-2-yl)benzamide (**6k**). White solid, 82% yield, m.p. 132–135 °C; ^1^H NMR (400 MHz, DMSO-*d*_6_) *δ* 9.09–9.02 (m, 1H), 8.25 (s, 1H), 7.83 (d, *J* = 8.3 Hz, 3H), 7.53 (d, *J* = 8.2 Hz, 2H), 7.35 (t, *J* = 7.7 Hz, 2H), 7.29 (t, *J* = 8.5 Hz, 2H), 5.67 (s, 1H), 4.47 (d, *J* = 5.9 Hz, 2H), 4.41 (d, *J* = 3.7 Hz, 2H), 1.44 (s, 3H); ^13^C NMR (100 MHz, DMSO-*d*_6_) *δ* 166.6, 150.9, 149.7, 145.4, 142.8, 133.5, 133.0, 130.7, 127.4, 127.2, 126.4, 125.8, 73.2, 59.8, 42.6, 27.7; HR-MS (ESI): calcd for C_19_H_19_ClN_4_O_2_ [M+H]^+^ 371.1270, found (ESI^+^) 371.1265.

*N*-(4-Chlorobenzyl)-4-(2-hydroxy-1-(1*H*-1,2,4-triazol-1-yl)propan-2-yl)benzamide (**6l**). White solid, 60% yield, m.p. 148–151 °C; ^1^H NMR (400 MHz, DMSO-*d*_6_) *δ* 9.02 (m, 1H), 8.24 (s, 1H), 7.82 (d, *J* = 9.4 Hz, 3H), 7.52 (d, *J* = 8.0 Hz, 2H), 7.36 (q, *J* = 9.6, 8.9 Hz, 4H), 5.66 (s, 1H), 4.45 (d, *J* = 5.7 Hz, 2H), 4.41 (s, 2H), 1.44 (s, 3H); ^13^C NMR (100 MHz, DMSO-*d*_6_) *δ* 166.5, 150.9, 149.7, 145.4, 139.3, 133.1, 131.8, 129.6, 128.7, 127.4, 125.7, 73.2, 59.8, 42.5, 27.7; HR-MS (ESI): calcd for C_19_H_19_ClN_4_O_2_ [M+H]^+^ 371.1270, found (ESI^+^) 371.1273.

*N*-(4-Bromobenzyl)-4-(2-hydroxy-1-(1*H*-1,2,4-triazol-1-yl)propan-2-yl)benzamide (**6m**). White solid, 82% yield, m.p. 74–77 °C; ^1^H NMR (400 MHz, DMSO-*d*_6_) *δ* 9.05 (m, 1H), 8.23 (s, 1H), 7.85–7.78 (m, 3H), 7.51 (d, *J* = 8.1 Hz, 4H), 7.27 (d, *J* = 8.1 Hz, 2H), 5.72 (s, 1H), 4.42 (d, *J* = 8.3 Hz, 4H), 1.44 (s, 3H); ^13^C NMR (100 MHz, DMSO-*d*_6_) *δ* 166.7, 150.9, 149.6, 145.3, 139.6, 133.0, 131.6, 130.0, 127.4, 125.7, 120.2, 73.2, 59.8, 42.5, 27.7; HR-MS (ESI): calcd for C_19_H_19_BrN_4_O_2_ [M+H]^+^ 415.0765, found (ESI^+^) 415.0763.

4-(2-Hydroxy-1-(1H-1,2,4-triazol-1-yl)propan-2-yl)-N-phenethylbenzamide (**7a**). White solid, 74% yield, m.p. 155–158 °C; ^1^H NMR (400 MHz, DMSO-*d*_6_) *δ* 8.52 (m, 1H), 8.24 (s, 1H), 7.85 (s, 1H), 7.76 (d, *J* = 8.1 Hz, 2H), 7.51 (d, *J* = 8.0 Hz, 2H), 7.25 (m, 5H), 5.65 (s, 1H), 4.40 (s, 2H), 3.48 (q, *J* = 8.0, 7.4 Hz, 2H), 2.84 (t, *J* = 7.5 Hz, 2H), 1.44 (s, 3H); ^13^C NMR (100 MHz, DMSO-*d*_6_) *δ* 166.5, 150.9, 149.4, 145.4, 140.1, 133.5, 129.2, 128.9, 127.3, 126.6, 125.6, 73.1, 59.8, 41.4, 35.6, 27.6; HR-MS (ESI): calcd for C_20_H_22_N_4_O_2_ [M+H]^+^ 351.1816, found (ESI^+^) 351.1821.

4-(2-Hydroxy-1-(1*H*-1,2,4-triazol-1-yl)propan-2-yl)-*N*-(4-methoxyphenethyl)benzamide (**7b**). White solid, 62% yield, m.p. 138–141 °C; ^1^H NMR (400 MHz, DMSO-*d*_6_) *δ* 8.53–8.46 (m, 1H), 8.24 (s, 1H), 7.85 (s, 1H), 7.76 (d, *J* = 8.1 Hz, 2H), 7.51 (d, *J* = 8.0 Hz, 2H), 7.15 (d, *J* = 8.1 Hz, 2H), 6.86 (d, *J* = 8.2 Hz, 2H), 5.66 (s, 1H), 4.41 (s, 2H), 3.71 (s, 3H), 3.43 (q, *J* = 6.8 Hz, 2H), 2.77 (t, *J* = 7.6 Hz, 2H), 1.44 (s, 3H); ^13^C NMR (100 MHz, DMSO-*d*_6_) *δ* 166.5, 158.2, 150.9, 149.4, 145.4, 133.5, 131.9, 130.1, 127.3, 125.6, 114.3, 73.1, 59.8, 55.5, 41.6, 34.7, 27.6; HR-MS (ESI): calcd for C_21_H_24_N_4_O_3_ [M+H]^+^ 381.1922, found (ESI^+^) 381.1929.

*N*-(2-Fluorophenethyl)-4-(2-hydroxy-1-(1*H*-1,2,4-triazol-1-yl)propan-2-yl)benzamide (**7c**). Light pink solid, 73% yield, m.p. 149–151 °C; ^1^H NMR (400 MHz, DMSO-*d*_6_) *δ* 8.55 (m, 1H), 8.24 (s, 1H), 7.85 (s, 1H), 7.74 (d, *J* = 8.0 Hz, 2H), 7.50 (d, *J* = 8.0 Hz, 2H), 7.28 (m, 2H), 7.14 (q, *J* = 8.8, 7.4 Hz, 2H), 5.65 (s, 1H), 4.40 (s, 2H), 3.48 (q, *J* = 6.7 Hz, 2H), 2.88 (t, *J* = 7.5 Hz, 2H), 1.43 (s, 3H); ^13^C NMR (100 MHz, DMSO-*d*_6_) *δ* 166.5, 161.3 (d, *J* = 243.3 Hz), 150.9, 149.4, 145.3, 133.5, 131.7 (d, *J* = 4.9 Hz), 128.8 (d, *J* = 8.0 Hz), 127.2, 126.6 (d, *J* = 16.0 Hz), 125.6, 124.9 (d, *J* = 3.2 Hz), 115.6 (d, *J* = 21.9 Hz), 73.1, 59.8, 41.2, 29.1, 27.6; HR-MS (ESI): calcd for C_20_H_21_FN_4_O_2_ [M+H]^+^ 369.1722, found (ESI^+^) 369.1731.

*N*-(4-Fluorophenethyl)-4-(2-hydroxy-1-(1*H*-1,2,4-triazol-1-yl)propan-2-yl)benzamide (**7d**). White solid, 74% yield, m.p. 160–162 °C; ^1^H NMR (400 MHz, DMSO-*d*_6_) *δ* 8.50 (m, 1H), 8.24 (s, 1H), 7.84 (s, 1H), 7.74 (d, *J* = 8.0 Hz, 2H), 7.50 (d, *J* = 8.0 Hz, 2H), 7.27 (t, *J* = 7.0 Hz, 2H), 7.11 (t, *J* = 8.7 Hz, 2H), 5.65 (s, 1H), 4.40 (s, 2H), 3.46 (q, *J* = 6.8 Hz, 2H), 2.83 (t, *J* = 7.5 Hz, 2H), 1.44 (s, 3H); ^13^C NMR (100 MHz, DMSO-*d*_6_) *δ* 166.5, 161.3 (d, *J* = 241.3 Hz), 150.9, 149.4, 145.3, 136.2 (d, *J* = 3.0 Hz), 133.5, 130.9 (d, *J* = 7.9 Hz), 127.3, 125.6, 115.5 (d, *J* = 21.0 Hz), 73.1, 59.8, 41.3, 34.7, 27.6; HR-MS (ESI): calcd for C_20_H_21_FN_4_O_2_ [M+H]^+^ 369.1722, found (ESI^+^) 369.1719.

*N*-(4-Chlorophenethyl)-4-(2-hydroxy-1-(1*H*-1,2,4-triazol-1-yl)propan-2-yl)benzamide (**7e**). White solid, 66% yield, m.p. 185–188 °C; ^1^H NMR (400 MHz, DMSO-*d*_6_) *δ* 8.50 (m, 1H), 8.24 (s, 1H), 7.84 (s, 1H), 7.74 (d, *J* = 8.1 Hz, 2H), 7.50 (d, *J* = 8.0 Hz, 2H), 7.34 (d, *J* = 8.1 Hz, 2H), 7.26 (d, *J* = 8.1 Hz, 2H), 5.65 (s, 1H), 4.40 (s, 2H), 3.47 (q, *J* = 6.8 Hz, 2H), 2.83 (t, *J* = 7.3 Hz, 2H), 1.43 (s, 3H); ^13^C NMR (100 MHz, DMSO-*d*_6_) *δ* 166.5, 150.9, 149.4, 145.3, 139.1, 133.5, 131.1, 128.7, 127.2, 125.6, 73.1, 59.8, 41.1, 34.8, 27.6; HR-MS (ESI): calcd for C_20_H_21_ClN_4_O_2_ [M+H]^+^ 385.1426, found (ESI^+^) 385.1422.

*N*-(2-Bromophenethyl)-4-(2-hydroxy-1-(1*H*-1,2,4-triazol-1-yl)propan-2-yl)benzamide (**7f**). White solid, 77% yield, m.p. 57–59 °C; ^1^H NMR (400 MHz, DMSO-*d*_6_) *δ* 8.55 (m, 1H), 8.24 (s, 1H), 7.84 (s, 1H), 7.75 (d, *J* = 8.1 Hz, 2H), 7.60 (d, *J* = 8.0 Hz, 1H), 7.50 (d, *J* = 8.1 Hz, 2H), 7.32 (d, *J* = 6.8 Hz, 2H), 7.16 (t, *J* = 8.0 Hz, 1H), 5.65 (s, 1H), 4.40 (s, 2H), 3.50 (q, *J* = 6.8 Hz, 2H), 2.98 (t, *J* = 7.3 Hz, 2H), 1.44 (s, 3H); ^13^C NMR (100 MHz, DMSO-*d*_6_) *δ* 166.6, 150.9, 149.4, 145.4, 139.1, 133.5, 133.0, 131.6, 129.0, 128.3, 127.3, 125.6, 124.5, 73.1, 59.8, 41.1, 35.8, 27.6; HR-MS (ESI): calcd for C_20_H_21_BrN_4_O_2_ [M+H]^+^ 429.0921, found (ESI^+^) 429.0927.

*N*-(4-Bromophenethyl)-4-(2-hydroxy-1-(1*H*-1,2,4-triazol-1-yl)propan-2-yl)benzamide (**7g**). White solid, 75% yield, m.p. 188–190 °C; ^1^H NMR (400 MHz, DMSO-*d*_6_) *δ* 8.53–8.47 (m, 1H), 8.24 (s, 1H), 7.84 (s, 1H), 7.74 (d, *J* = 8.2 Hz, 2H), 7.53–7.45 (m, 4H), 7.20 (d, *J* = 8.1 Hz, 2H), 5.65 (s, 1H), 4.40 (s, 2H), 3.47 (q, *J* = 6.9 Hz, 2H), 2.82 (t, *J* = 7.2 Hz, 2H), 1.43 (s, 3H); ^13^C NMR (100 MHz, DMSO-*d*_6_) *δ* 166.5, 150.9, 149.4, 145.4, 139.5, 133.5, 131.7, 131.5, 127.3, 125.7, 119.7, 73.1, 59.8, 41.0, 34.9, 27.6; HR-MS (ESI): calcd for C_20_H_21_BrN_4_O_2_ [M+H]^+^ 429.0921, found (ESI^+^) 429.0917.

#### 2.2.2. Synthesis of Mefentrifluconazole

Mefentrifluconazole was synthesized according to the reported methods [31,32,33]. It was smoothly prepared from 1-(4-fluoro-2-(trifluoromethyl)phenyl)ethenone as a raw material via etherification, epoxidation, and ring-opening reactions.

### 2.3. In Vitro Target Compounds against Seven Phytopathogenic Fungi

We selected seven common and representative phytopathogenic fungi/oomycete that are harmful to crops (cereals, fruits, and vegetables) and have a range of impacts to test the newly synthesized compounds, including *Pyricularia oryzae* (*P. oryzae*), *Sclerotinia sclerotiorum* (*S. sclerotiorum*), *Fusarium oxysporium* f. sp. *Cucumeris* (*F. oxysporium* f. sp. *Cucumeris*), *Cercospora arachidicola Hori* (*C. arachidicola Hori*), *P. piricola, Rhizoctonia cerealis* (*R. cerealis*), and *Phytophthora capsici* (*P. capsici*), with the agricultural fungicide mefentrifluconazole as the control [34,35,36,37,38]. The in vitro antifungal/anti-oomycete activity test was performed using the mycelial growth rate method to evaluate the activity of the compounds. The literature was provided in the Supporting Information.

### 2.4. Calculation Procedures for Molecular Docking Research

The 3D crystal structure of C-14α demethylase (PDB code: 3L4D) was downloaded from the protein data bank (PDB). Detailed procedures are provided in the support information.

## 3. Results

### 3.1. Chemicals

**Scheme 1:**
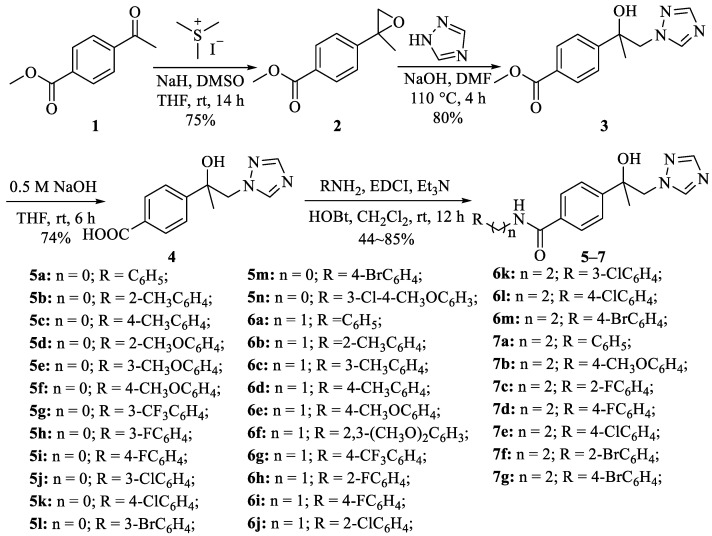
Synthesis of the compounds **5a**–**5n**, **6a**–**6m**, and **7a**–**7g**.

The synthesis of intermediates and target compounds was performed as shown in Scheme 1. The intermediate **2**, **3**, and **4** were prepared with a similar method described by Theodorou and Gebhardt [32,39]. Intermediate **4** was reacted with various substituted amines in the presence of 1-ethyl-3-(3-dimethylaminopropyl)carbodiimide hydrochloride (EDCI) and 1-hydroxybenzotriazole (HOBt) to obtain the target compounds **5**–**7** [40]. The chemical structures of all target compounds were confirmed by ^1^H and ^13^C NMR and HRMS and characterization data are provided in the Supplementary Materials.

### 3.2. In Vitro Antifungal/Anti-Oomycete Activities of Target Compounds ***5a***–***5n***, ***6a***–***6m***, and ***7a***–***7g***

A series of novel 1,2,4-triazole derivatives were tested for in vitro antifungal/anti-oomycete activity against seven phytopathogenic fungi at 50 μg/mL. The results were compared to the commercialized fungicide mefentrifluconazole, as indicated in Table 1. According to the results in Table 1, compounds **5k**, **6h**, and **7d** showed higher antifungal activities compared to mefentrifluconazole against *P. piricola* (80% versus 54%, 92% versus 54%, and 66% versus 54% inhibition, respectively). For *P. capsici*, most of the compounds especially compounds **5j**, **6k**, and **6m**, showed much better anti-oomycete activity (90%, 73%, and 73%, respectively) than that of mefentrifluconazole (32%).

To understand the antifungal/anti-oomycete activities of compounds **5j**, **6h**, **6k**, and **6m** more clearly and intuitively, the half maximal effective concentration (EC_50_) value of compounds **5j**, **6h**, **6k**, and **6m** were determined and the results are shown in Table 2. Compound **6h** (EC_50_ = 13.095 μg/mL) showed about three times higher potency than mefentrifluconazole (EC_50_ = 39.516 μg/mL). Compounds **5j**, **6k**, and **6m** exhibited excellent in vitro activity effects against *P. capsici*, with EC_50_ values of 17.362, 29.970, and 33.152 μg/mL, superior to the intrinsic activity of mefentrifluconazole (EC_50_ = 75.433 μg/mL).

### 3.3. Molecular Docking Research

To further elucidate the possible mechanism of the interaction between designed 1,2,4-triazole derivatives and CPY51, AutoDock Vina 1.1.2 was used for molecular docking [41]. It can be proven that there are some H-bond interactions and strong binding affinity between 1,2,4-triazole derivatives **5j** and **6h** containing amide fragments and CPY51.

## 4. Discussion

### 4.1. Synthesis

According to a similar approach reported, the 4-position acetyl of methyl 4-acetylbenzoate was epoxidized with trimethylsulfonium iodide in the presence of NaH to obtain compound **2** and then through a substitution reaction with 1,2,4-triazole in DMF to obtain compound **3**. The key intermediate **4** was obtained from **3** through hydrolysis in the presence of sodium hydroxide, which was amidated with aniline, benzylamine, and phenethylamine derivatives in the presence of 1-ethyl-3-(3-dimethylaminopropyl)carbodiimide hydrochloride (EDCI) and 1-hydroxybenzotriazole (HOBt) to obtain the target compounds **5**–**7** (Scheme 1) [40]. The agricultural fungicide mefentrifluconazole was synthesized according to the procedure reported [32]. With 1-(4-Fluoro-2-(trifluoromethyl)phenyl)ethanone as the starting material, a series of reactions by substitution, epoxidation, and triazole substitution were performed and mefentrifluconazole was finally synthesized. Aniline, benzylamine, and phenethylamine derivative fragments were mainly introduced into the isopropanol triazole skeleton by the active structure splicing strategy to achieve structural diversity in the derivation of isopropanol triazole compounds.

The structures of all compounds were characterized and confirmed by ^1^H NMR, ^13^C NMR, and HRMS. Mefentrifluconazole is consistent with the literature report. In the ^1^H NMR spectra of target compounds, the following can be said: (i) for compounds **5a**–**5n**, the characteristic amide group proton signal can be found around 10.0 ppm as a single peak; (ii) for compounds **6a**–**6m**, the characteristic amide group proton signal can be found around 9.0 ppm as multiple peaks and the double peak at around 4.0 ppm was assigned as the CH_2_ on the benzyl; and (iii) for compounds **7a**–**7g**, the characteristic amide group proton signal can be found around 8.5 ppm as a multiple peak and the triple peak at around 2.8 ppm and the quadruple peak at around 3.5 ppm were assigned as the CH_2_ on the phenylethyl. In the ^13^C NMR spectra, the single peak around 42 ppm was assigned as the CH_2_ on the benzyl and the single peak around 34 ppm and 41 ppm were assigned as the CH_2_ on the phenylethyl. These spectroscopic features confirmed that aniline, benzylamine, and phenethylamine derivative fragments were successfully introduced into the isopropanol triazole skeleton.

### 4.2. Structure–Activity Relationship (SAR) Analysis for the Antifungal/Anti-Oomycete Activity

The agricultural fungicide mefentrifluconazole was chosen as one positive control. The preliminary in vitro antifungal activities of target compounds **5a**–**5n**, **6a**–**6m**, and **7a**–**7g** are shown in Table 1 and the EC_50_ values of the selected compounds are shown in Table 2.

As shown in Table 1, most target compounds showed poor antifungal activities against *P. oryzae*, *F. oxysporium* f. sp. *Cucumeris*, *C. arachidicola Hori*, and *R. cerealis* at the concentration of 50 μg/mL. For *S. sclerotiorum*, only two compounds **6b** and **6j** displayed good fungicidal activities (more than 70% inhibition rate), which was much lower than mefentrifluconazole (96%). To our excitement, most of the compounds had similar or better antifungal activities compared to mefentrifluconazole against *P. piricola* inhibitory activity, especially compounds **5k**, **6h**, and **7d**, which showed higher antifungal activities compared to mefentrifluconazole (80% versus 54%, 92% versus 54%, and 66% versus 54% inhibition, respectively). Most compounds displayed good anti oomycete activities against *P. capsici*, with compounds **5g**, **5j**, **6c**, **6g**, **6k**, and **6m** showing greatly superior inhibition rates (71%, 90%, 71%, 71%, 73%, and 73%, respectively) with mefentrifluconazole (32%).

For the antifungal effect of different substituted aniline derivatives against *P. oryzae*, *S. sclerotiorum*, and *P. capsici*, the meta substitution on the benzene ring was better than that of the para substitution. For example, compound **5e**, bearing a methoxy at the meta position of the phenyl ring, showed much higher antifungal activity than compound **5f** containing a para substitution. This effect could also be verified by the results that compounds **5h**, **5j**, and **5l** were more active than compounds **5i**, **5k**, and **5m**, respectively.

In terms of anti-oomycete activities against *P. capsici*, substituents on the benzene ring of benzylamine derivatives, whether electron-withdrawing groups [compounds **6b** (2-CH_3_C_6_H_4_), **6c** (3-CH_3_C_6_H_4_), and **6d** (4-CH_3_C_6_H_4_)] or electron-rich groups [compounds **6j** (2-ClC_6_H_4_), **6k** (3-ClC_6_H_4_), and **6l** (4-ClC_6_H_4_)], the meta substitution was better than that of the para substitution, which was superior to that of the ortho substitution. For aniline derivatives, position 3 of the benzene ring was substituted with –Cl and the corresponding compound **5j** exhibited optimal anti-oomycete activity. In terms of antifungal activities against *S. sclerotiorum*, substituents on the benzene ring of benzylamine derivatives, whether electron-withdrawing groups [compounds **6b** (2-CH_3_C_6_H_4_), **6c** (3-CH_3_C_6_H_4_), and **6d** (4-CH_3_C_6_H_4_)] or electron-rich groups [compounds **6j** (2-ClC_6_H_4_), **6k** (3-ClC_6_H_4_), and **6l** (4-ClC_6_H_4_)], the ortho substitution was better than that of the meta substitution, which was superior to that of the para substitution. For *P. piricola*, position 2 of the benzyl ring was substituted with –F and the corresponding compound **6h** exhibited optimal antifungal activity. Therefore, it is further shown that the ortho substitution on the benzyl ring is beneficial for antifungal activity.

As shown in Table 2, compound **6h** (EC_50_ = 13.095 μg/mL) showed about three times higher potency than mefentrifluconazole (EC_50_ = 39.516 μg/mL). It is further shown that compound **6h** has an excellent antifungal effect against *P. piricola*. For *P. capsici*, even though compounds **5j**, **6k**, and **6m** only exhibited moderate anti oomycete activity, with EC_50_ values of 17.362, 29.970, and 33.152 μg/mL, respectively, they were still much better than those of mefentrifluconazole (EC_50_ = 75.433 μg/mL). Remarkably, the substitution of –Cl in the meta position of the benzene ring may be an important moiety to enhance the anti-oomycete activity of the compounds.

### 4.3. Molecular Docking

**Figure 3:**
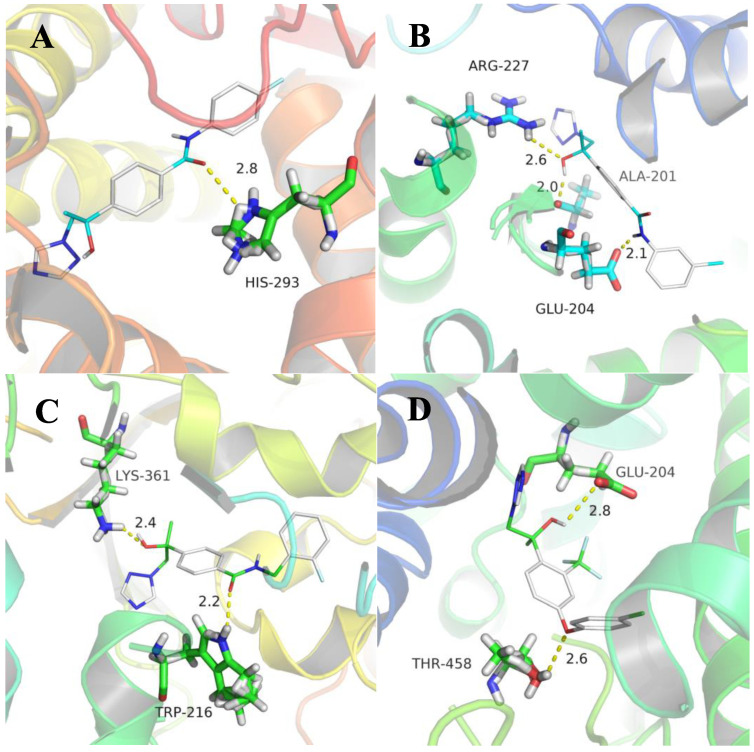
Molecule docking results of **5i** (**A**), **5j** (**B**), **6h** (**C**), and mefentrifluconazole (**D**) with CYP51 (PDB code: 3L4D).

Since the newly synthesized 1,2,4-triazole derivatives were derived from mefentrifluconazole, which can be combined with the iron atom in the heme-iron active center of 14α-demethylase (CYP51), the interaction between the selected compounds and CYP51 was further studied. To elucidate the possible mechanism of designed compounds, compounds **5i**, **5j**, **6h**, and mefentrifluconazole were selected for the docking simulation and the model was generated based on the reported crystal complex (PDB code: 3L4D) [42]. Major hydrogen bonds between the compounds and the amino acid residues are shown in Figure 3. In the binding modes of **5j**, the oxygen of the hydroxyl group formed a hydrogen bond (2.6 Å) with the NH of amino acid residue ARG-227, the H of the hydroxyl group formed a hydrogen bond (2.0 Å) with the oxygen of amino acid residue ALA-201, and the H of the amide formed a hydrogen bond (2.1 Å) with the oxygen of amino acid residue GLU-204 (Figure 3B). In the docking models with **6h**, the oxygen of the hydroxyl group formed a hydrogen bond with the NH of amino acid residue LYS-361 and the oxygen of the amide and the NH of amino acid residue TRP-216 formed a hydrogen bond with corresponding binding distances of 2.4 and 2.2, respectively (Figure 3C). In the binding modes of mefentrifluconazole, the H of the hydroxyl group formed a hydrogen bond (2.8 Å) with the oxygen of amino acid residue GLU-204 and the oxygen on the ether group formed a hydrogen bond (2.6 Å) with the OH of amino acid residue THR-458 (Figure 3D). Compounds **5j**, **6h**, and mefentrifluconazole are similar in that both have active sites on the hydroxyl group. However, compound **5i** only formed a hydrogen bond (2.8 Å) between the oxygen of the amide and the NH of amino acid residue HIS-293 (Figure 3A). These results may partly explain the differences in the aforementioned antifungal phenotypic profiles, with the introduction of carboxamide group increasing its antifungal activity but the hydroxyl group remaining the main active group interacting with CPY51.

In summary, a series of novel 1,2,4-triazole derivatives containing carboxamide fragments were designed and synthesized. Their fungicidal activities against seven kinds of phytopathogenic fungi at 50 μg/mL were evaluated. The bioassay results showed that most compounds had better inhibitory effects against *P. piricola*, especially compounds **5k**, **6h**, and **7d**, which showed higher antifungal activities compared to commercial DMI fungicide mefentrifluconazole (80 versus 54%, 92 versus 54%, and 66 versus 54% inhibition, respectively). Compound **6h** (EC_50_ = 13.095 μg/mL) showed about three times higher potency than mefentrifluconazole (EC_50_ = 39.516 μg/mL). For *P. capsici*, most of the compounds showed good anti-oomycete activity, especially compounds **5j**, **6k**, and **6m** (90, 73, and 73%, respectively), which showed much better inhibition than mefentrifluconazole (32%). Of these, compound **5j** (EC_50_ = 17.362 μg/mL) showed about four times higher potency than mefentrifluconazole (EC_50_ = 75.433 μg/mL). Molecular docking analysis revealed that compounds **5j** and **6h** possessed a stronger affinity to CYP51. All of these results showed that compound **6h** was a novel and promising candidate as a fungicide for the control of apple ring rot and compound **5j** was a candidate for the control of oomycetes. Remarkably, the chemical structure of mefentrifluconazole contains a chiral center with a pair of enantiomers and further study of its stereoselective differences from the chiral level will be a key direction for future research.

## Figures and Tables

**Table 1:** In vitro antifungal/anti-oomycete activities of the compounds **5a**–**5n**, **6a**–**6m**, **7a**–**7m,** and mefentrifluconazole at 50 μg/mL ^1^.

Compound	Inhibition Rate (%)						
	*P.o.* ^2^	*S.s.* ^2^	*F.c.* ^2^	*C.h.* ^2^	*P.p.* ^2^	*R.c.* ^2^	*P.c.* ^2^
**5a**	25 ± 3	57 ± 3	28 ± 3	5 ± 1	48 ± 2	17 ± 3	37 ± 2
**5b**	25 ± 3	59 ± 2	14 ± 3	12 ± 2	54 ± 1	14 ± 2	59 ± 3
**5c**	25 ± 3	52 ± 2	24 ± 3	0 ± 0	48 ± 2	21 ± 2	44 ± 1
**5d**	25 ± 3	61 ± 1	8 ± 3	12 ± 2	48 ± 2	14 ± 2	59 ± 3
**5e**	50 ± 3	63 ± 3	14 ± 3	10 ± 1	54 ± 1	18 ± 2	54 ± 2
**5f**	13 ± 3	52 ± 2	14 ± 3	12 ± 2	48 ± 2	20 ± 3	24 ± 1
**5g**	38 ± 3	50 ± 3	14 ± 3	7 ± 1	48 ± 2	17 ± 3	71 ± 2
**5h**	50 ± 3	61 ± 1	18 ± 3	27 ± 1	35 ± 1	44 ± 3	54 ± 2
**5i**	25 ± 3	37 ± 3	22 ± 3	2 ± 1	35 ± 1	20 ± 3	17 ± 2
**5j**	44 ± 1	52 ± 2	24 ± 3	10 ± 1	37 ± 2	17 ± 3	90 ± 2
**5k**	25 ± 3	50 ± 3	26 ± 3	24 ± 1	80 ± 2	40 ± 3	22 ± 3
**5l**	50 ± 3	65 ± 2	28 ± 3	49 ± 1	44 ± 1	20 ± 3	68 ± 1
**5m**	25 ± 3	44 ± 3	18 ± 3	10 ± 1	39 ± 2	28 ± 2	12 ± 2
**5n**	13 ± 3	50 ± 3	18 ± 3	17 ± 2	51 ± 2	34 ± 2	22 ± 3
**6a**	13 ± 3	46 ± 2	14 ± 3	12 ± 2	48 ± 2	34 ± 2	37 ± 2
**6b**	25 ± 3	72 ± 2	16 ± 3	7 ± 1	48 ± 2	58 ± 2	17 ± 2
**6c**	13 ± 3	65 ± 2	16 ± 3	7 ± 1	48 ± 2	25 ± 2	71 ± 2
**6d**	63 ± 3	54 ± 1	12 ± 3	5 ± 1	48 ± 2	17 ± 3	32 ± 2
**6e**	25 ± 3	37 ± 3	18 ± 3	17 ± 2	48 ± 2	26 ± 1	42 ± 3
**6f**	25 ± 3	48 ± 1	24 ± 3	10 ± 1	46 ± 2	17 ± 3	22 ± 3
**6g**	50 ± 3	37 ± 3	18 ± 3	0 ± 0	52 ± 1	26 ± 1	71 ± 2
**6h**	25 ± 3	67 ± 1	30 ± 3	24 ± 1	92 ± 1	20 ± 3	24 ± 1
**6i**	25 ± 3	48 ± 1	18 ± 3	7 ± 1	42 ± 1	31 ± 2	37 ± 2
**6j**	13 ± 3	76 ± 2	12 ± 3	12 ± 2	39 ± 2	20 ± 3	44 ± 1
**6k**	44 ± 1	63 ± 3	8 ± 3	7 ± 1	53 ± 2	17 ± 3	73 ± 2
**6l**	38 ± 3	30 ± 1	12 ± 3	7 ± 1	44 ± 1	19 ± 1	61 ± 3
**6m**	13 ± 3	22 ± 2	0 ± 0	5 ± 1	42 ± 1	17 ± 3	73 ± 2
**7a**	38 ± 3	44 ± 3	14 ± 3	7 ± 1	54 ± 1	28 ± 2	24 ± 1
**7b**	13 ± 3	44 ± 3	16 ± 3	2 ± 1	42 ± 1	20 ± 3	17 ± 2
**7c**	6 ± 1	33 ± 2	14 ± 3	7 ± 1	48 ± 2	19 ± 1	32 ± 2
**7d**	6 ± 1	44 ± 3	8 ± 3	5 ± 1	66 ± 1	17 ± 3	37 ± 2
**7e**	50 ± 3	54 ± 1	12 ± 3	5 ± 1	54 ± 1	22 ± 1	46 ± 1
**7f**	6 ± 1	37 ± 3	18 ± 3	24 ± 1	44 ± 1	46 ± 1	24 ± 1
**7g**	6 ± 1	44 ± 3	8 ± 3	2 ± 1	48 ± 2	18 ± 2	24 ± 1
mefentrifluconazole	100	96 ± 2	70 ± 3	78 ± 3	54 ± 1	100	32 ± 2

^1^ The experiments were repeated three times. All results are expressed as mean ± SD. ^2^ *P.o.* = *Pyricularia oryzae*; *S.s.* = *Sclerotinia sclerotiorum*; *F.c.* = *Fusarium oxysporium* f. sp. *cucumeris*; *C.h.* = *Cercospora arachidicola Hori*; *P.p.* = *Physalospora piricola*; *R.c.* = *Rhizoctonia cerealis*; *P.c.* = *Phytophthora capsici*.

**Table 2:** In vitro EC_50_ value (μg/mL) of selected compounds against P. piricola and P. capsici ^1^.

Strain	Compound	EC_50_ (μg/mL)	95% Confidence Interval	Regression Equation	*R* ^2^
*P. piricola*	**6h**	13.095	11.423–15.026	y = 3.762x − 4.203	0.988
	mefentrifluconazole	39.516	33.822–46.867	y = 3.147x − 5.024	0.942
*P. capsici*	**5j**	17.362	11.664–27.413	y = 4.008x − 4.968	0.962
	**6k**	29.970	20.794–44.597	y = 3.256x − 4.808	0.955
	**6m**	33.152	28.925–38.217	y = 3.745x − 5.694	0.984
	mefentrifluconazole	75.433	62.167–96.948	y = 3.000x − 5.632	0.980

^1^ The experiments were repeated three times.

## Data Availability

Data are contained within the article and Supplementary Materials.

## Supplementary Materials

The following supporting information can be downloaded at https://www.mdpi.com/article/10.3390/jof10020160/s1. Section S1: Detailed bioassay procedures for the in vitro antifungal/anti-oomycete activities; Section S2: Calculation procedures for molecular docking research; Section S3: Copies of NMR spectra (Figures S1–S74). References [43,44] were cited in Supplementary Materials.
