# Supplementary material for: Design, Synthesis, and Antifungal/Anti-Oomycete Activities of Novel 1,2,4-Triazole Derivatives Containing Carboxamide Fragments
Source: J Fungi (Basel). 2024 Feb 19;10(2):160. doi: 10.3390/jof10020160 (PMC10890616; doi:10.3390/jof10020160)
Supplement: Supplementary file 1 [file jof-10-00160-s001.zip › jof-2839271-supplementary.pdf]

Supplementary Material

Design, Synthesis, and Antifungal/Anti-oomycete Activities of Novel 1,2,4-Triazole Derivatives Containing Carboxamide Fragments

Jiali Wang †, Haoran Shi † and Aidang Lu *

School of Chemical Engineering and Technology, Hebei University of Technology, Tianjin 300401, China; galizhu999@163.com (J.W.); shr15130128422@163.com (H.S.)

* Correspondence: luaidang@hebut.edu.cn; Tel. +86-22-60202812

Contents

Section S1: Detailed bioassay procedures for the in vitro antifungal/anti-oomycete activities ········ 3

Section S2: Calculation procedures for molecular docking research ········ 3

Section S3: Copies of NMR spectra (Figures S1–S74) ········ 4

Figures S1–S2. ^{1}H NMR and ^{13}C NMR spectra of **2** ········ 4

Figures S3–S4. ^{1}H NMR and ^{13}C NMR spectra of **3** ········ 5

Figures S5–S6. ^{1}H NMR and ^{13}C NMR spectra of **4** ········ 6

Figures S7–S8. ^{1}H NMR and ^{13}C NMR spectra of **5a** ········ 7

Figures S9–S10. ^{1}H NMR and ^{13}C NMR spectra of **5b** ········ 8

Figures S11–S12. ^{1}H NMR and ^{13}C NMR spectra of **5c** ········ 9

Figures S13–S14. ^{1}H NMR and ^{13}C NMR spectra of **5d** ········ 10

Figures S15–S16. ^{1}H NMR and ^{13}C NMR spectra of **5e** ········ 11

Figures S17–S18. ^{1}H NMR and ^{13}C NMR spectra of **5f** ········ 12

Figures S19–S20. ^{1}H NMR and ^{13}C NMR spectra of **5g** ········ 13

Figures S21–S22. ^{1}H NMR and ^{13}C NMR spectra of **5h** ········ 14

Figures S23–S24. ^{1}H NMR and ^{13}C NMR spectra of **5i** ········ 15

Figures S25–S26. ^{1}H NMR and ^{13}C NMR spectra of **5j** ········ 16

Figures S27–S28. ^{1}H NMR and ^{13}C NMR spectra of **5k** ········ 17

Figures S29–S30. ^{1}H NMR and ^{13}C NMR spectra of **5l** ········ 18

Figures S31–S32. ^{1}H NMR and ^{13}C NMR spectra of **5m**······19

Figures S33–S34. ^{1}H NMR and ^{13}C NMR spectra of **5n**······20

Figures S35–S36. ^{1}H NMR and ^{13}C NMR spectra of **6a**······21

Figures S37–S38. ^{1}H NMR and ^{13}C NMR spectra of **6b**······22

Figures S39–S40. ^{1}H NMR and ^{13}C NMR spectra of **6c**······23

Figures S41–S42. ^{1}H NMR and ^{13}C NMR spectra of **6d**······24

Figures S43–S44. ^{1}H NMR and ^{13}C NMR spectra of **6e**······25

Figures S45–S46. ^{1}H NMR and ^{13}C NMR spectra of **6f**······26

Figures S47–S48. ^{1}H NMR and ^{13}C NMR spectra of **6g**······27

Figures S49–S50. ^{1}H NMR and ^{13}C NMR spectra of **6h**······28

Figures S51–S52. ^{1}H NMR and ^{13}C NMR spectra of **6i**······29

Figures S53–S54. ^{1}H NMR and ^{13}C NMR spectra of **6j** 30

Figures S55–S56. ^{1}H NMR and ^{13}C NMR spectra of **6k**······31

Figures S57–S58. ^{1}H NMR and ^{13}C NMR spectra of **6l**······32

Figures S59–S60. ^{1}H NMR and ^{13}C NMR spectra of **6m**······33

Figures S61–S62. ^{1}H NMR and ^{13}C NMR spectra of **7a**······34

Figures S63–S64. ^{1}H NMR and ^{13}C NMR spectra of **7b**······35

Figures S65–S66. ^{1}H NMR and ^{13}C NMR spectra of **7c**······36

Figures S67–S68. ^{1}H NMR and ^{13}C NMR spectra of **7d**······37

Figures S69–S70. ^{1}H NMR and ^{13}C NMR spectra of **74**······38

Figures S71–S72. ^{1}H NMR and ^{13}C NMR spectra of **7f**······39

Figures S73–S74. ^{1}H NMR and ^{13}C NMR spectra of **7g**······40

Section S1. Detailed bioassay procedures for the in vitro antifungal/anti-oomycete activities

The fungicidal activities of compounds were evaluated in mycelial growth tests conducted in artificial media against 7 plant pathogens at a rate of 50 μg/mL. Each test compound was dissolved in a suitable amount of acetone and diluted with water containing 0.1% TW-80 to a concentration of 500 μg/mL. To each petri dish was added 1 mL of the test solution and 9 mL of culture medium to make a 50 μg/mL concentration of the test compound, while in another petri dish was added 1 mL distilled water containing 0.1% TW-80 and 9 mL of culture medium as a blank control. A 4 mm diameter of hyphal growth was cut using a hole puncher on a growing fungal culture and the hyphae were moved to the petri dish containing the test compound. Each assay was performed three times. The dishes were stored in controlled environment cabinets (24 ± 1 °C) for 4 days, after which the diameter of mycelial growth was measured and the percentage inhibition was calculated using the following equation: Percentage inhibition (%) = (averaged diameter of mycelia in blank controls—averaged diameter of mycelia in medicated tablets)/(averaged diameter of mycelia in blank controls—4 mm) × 100 [43].

Section S2. Calculation procedures for molecular docking research

The calculation procedures for molecular docking research consist of four steps [44].

Receptor Preparation. The 3D crystal structure of C-14α demethylase (PDB code: 3L4D) was downloaded from the protein data bank (PDB) and this was used as the receptor for molecular docking. Water molecules were removed from the target protein and hydrogen atoms were added using AutoDock Tools prior to molecular docking.

Ligand preparation. Target compounds are drawn using ChemOffice 2015 as ligands followed by management of its conformer and the minimisation process.

Molecular Docking Using AutoDock Vina. The input files for AutoDock Vina were prepared using AutoDock Tools. The protein was placed in a grid box (grid parameters: center x = 38.01, center y = -33.901, center z = -28.152, size x = 102.63, size y = 76.506, size z = 117.58), using AutoDock Vina at 1.00 Å to define the binding site. The docking procedure was performed using the instructed command prompts.

Analyzing and Output Visualisation using PyMOL. The docking poses were ranked according to their docking scores. The scoring function in Auto Dock was used to predict the binding affinity of one ligand to the receptor molecule. The conformation with the lowest binding affinity was selected for further analysis after the docking process. The docking results included the locations of hydrogen bonds and closely interacting residues were performed by PyMOL software.

References

43. Zhao, H.P.; Liu, Y.X.; Cui, Z.P.; Beattie, D.; Gu, Y.C.; Wang, Q.M. Design, synthesis, and biological activities of arylmethylamine substituted chlorotriazine and methylthiotriazine compounds. *J. Agric. Food Chem.* **2011**, *59*, 11711–11717.
44. Seyedi, S.S.; Shukri, M.; Hassandarvish, P.; Oo, A.; Muthu, S.E.; Abubakar, S.; Zandi, K. Computational approach towards exploring potential anti-chikungunya activity of selected flavonoids. *Sci. Rep.* **2016**, *6*, 24027.

Section S3. Figures S1–S74. ^{1}H, and ^{13}C NMR spectra of 2–7

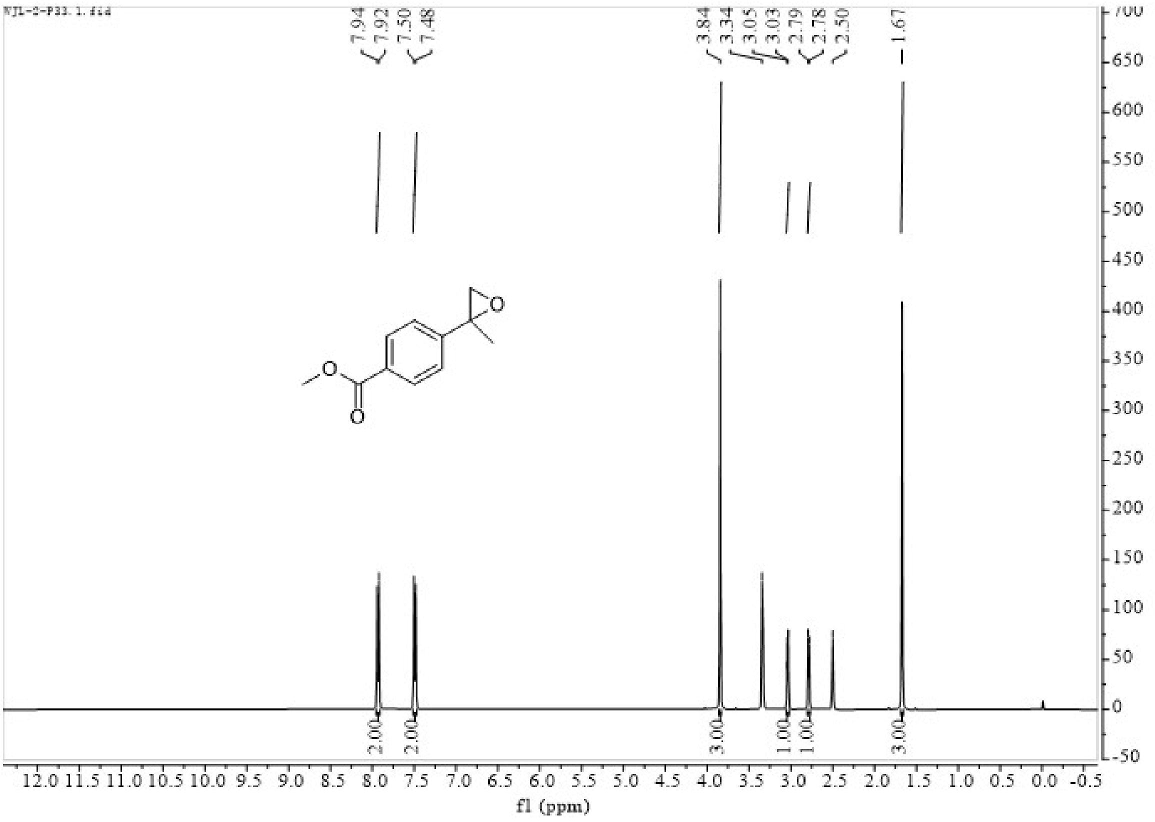

Figure S1. ^{1}H NMR spectrum of **2**

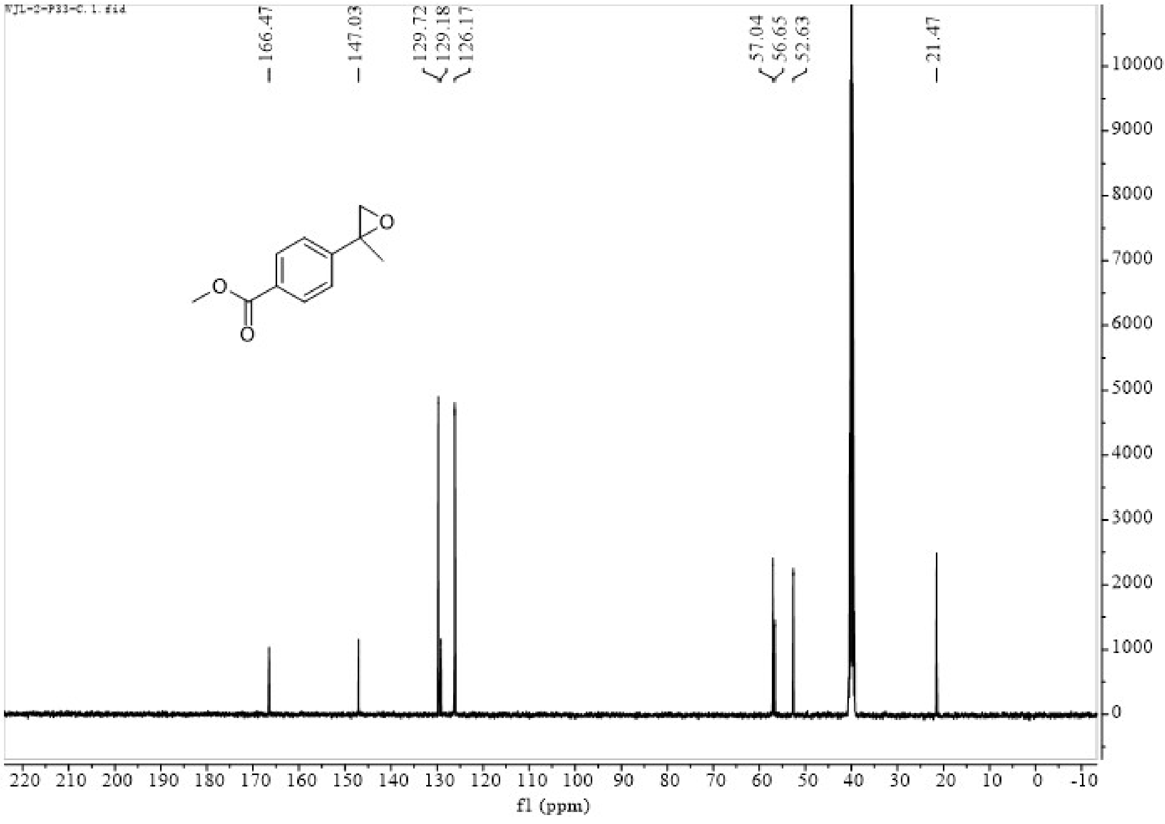

Figure S2. ^{13}C NMR spectrum of **2**

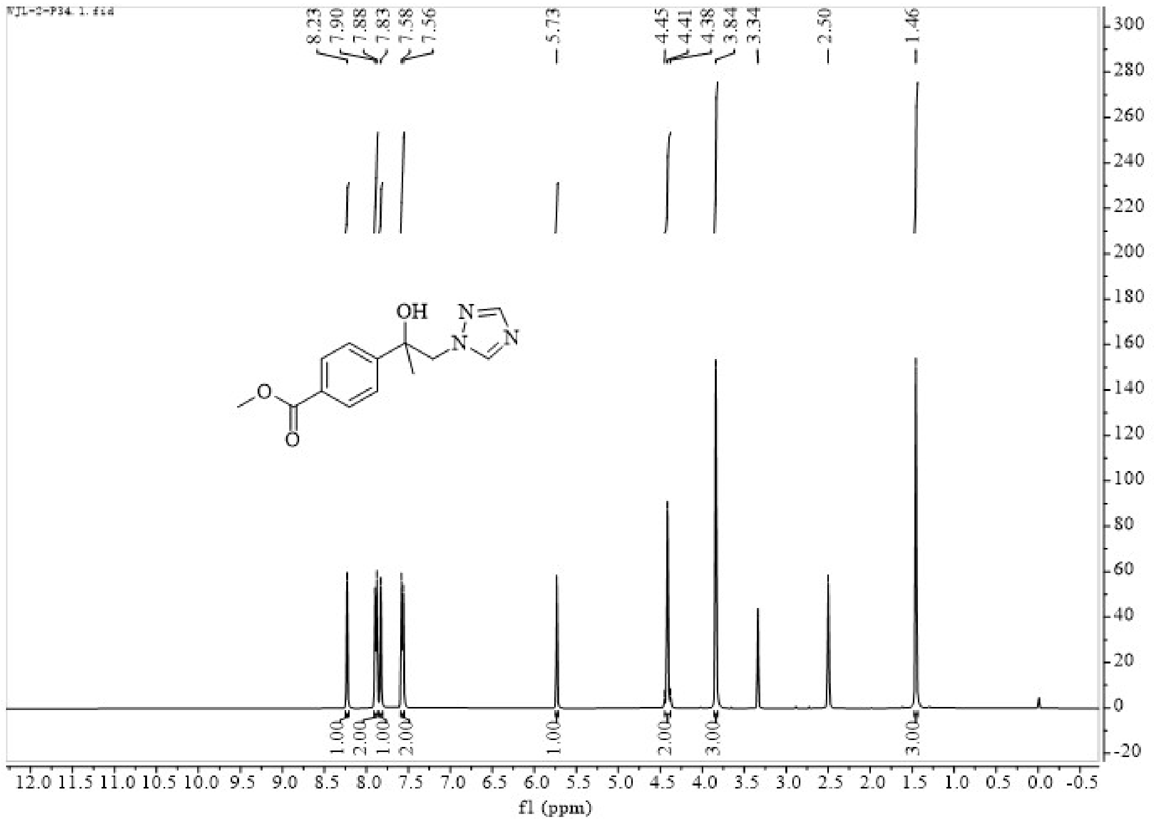

Figure S3. ^{1}H NMR spectrum of **3**

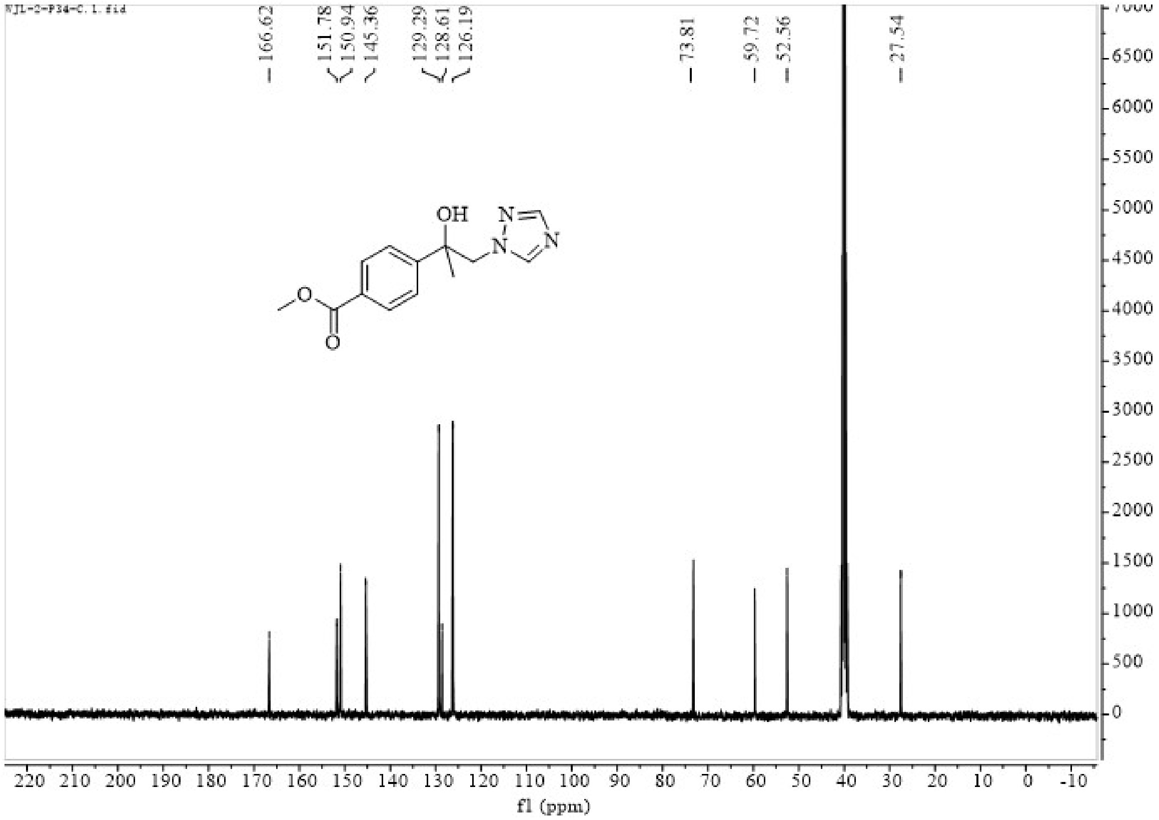

Figure S4. ^{13}C NMR spectrum of **3**

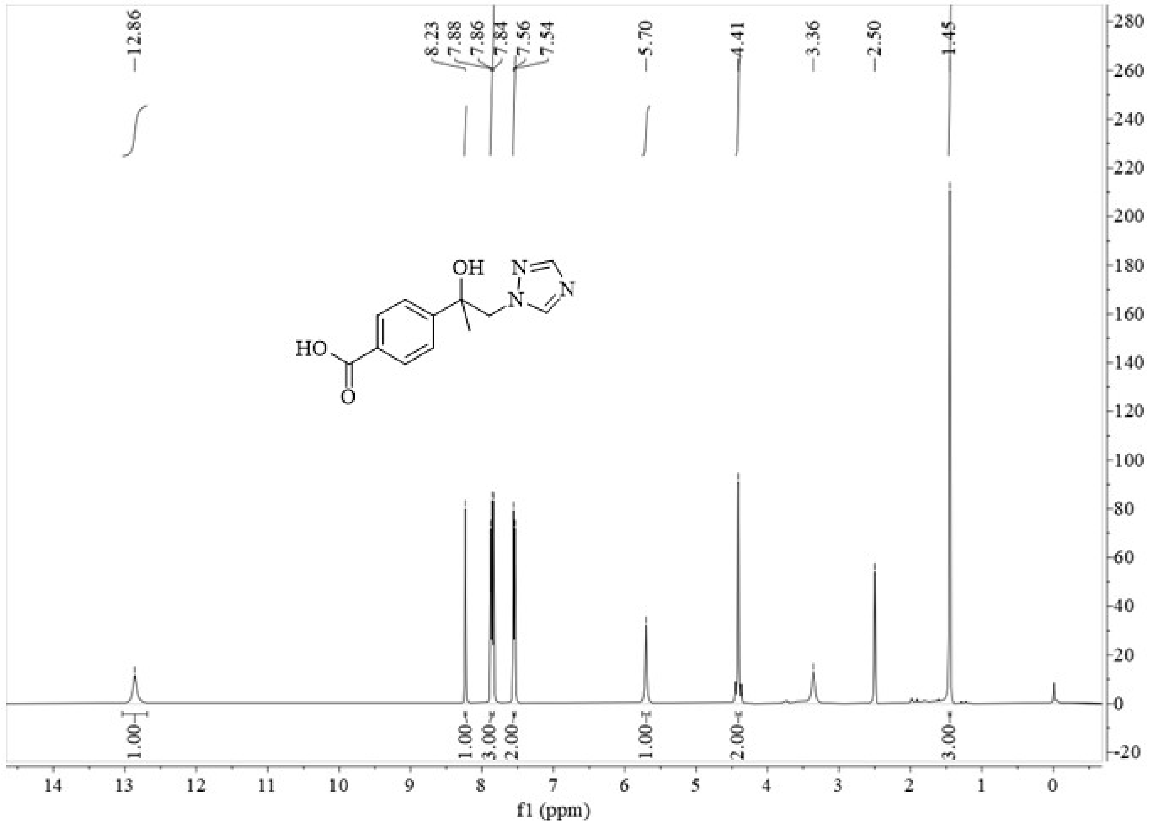

Figure S5. ^{1}H NMR spectrum of **4**

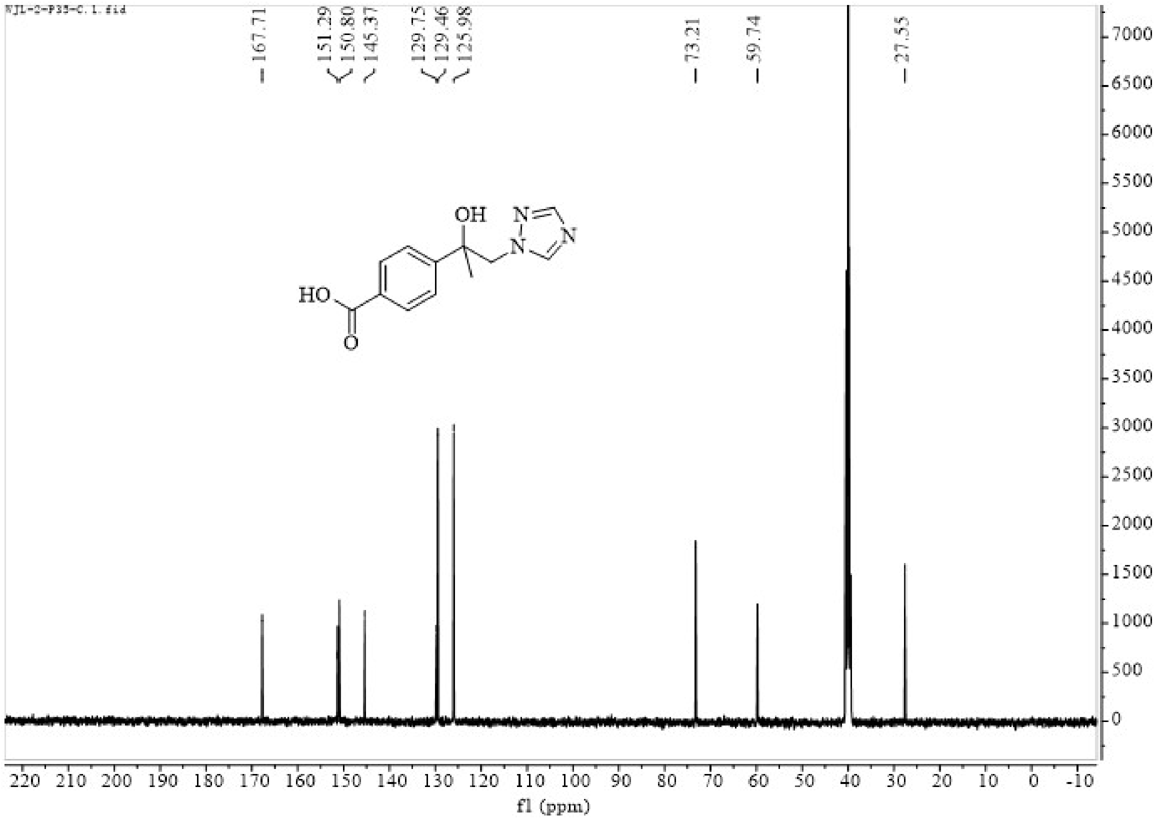

Figure S6. ^{13}C NMR spectrum of **4**

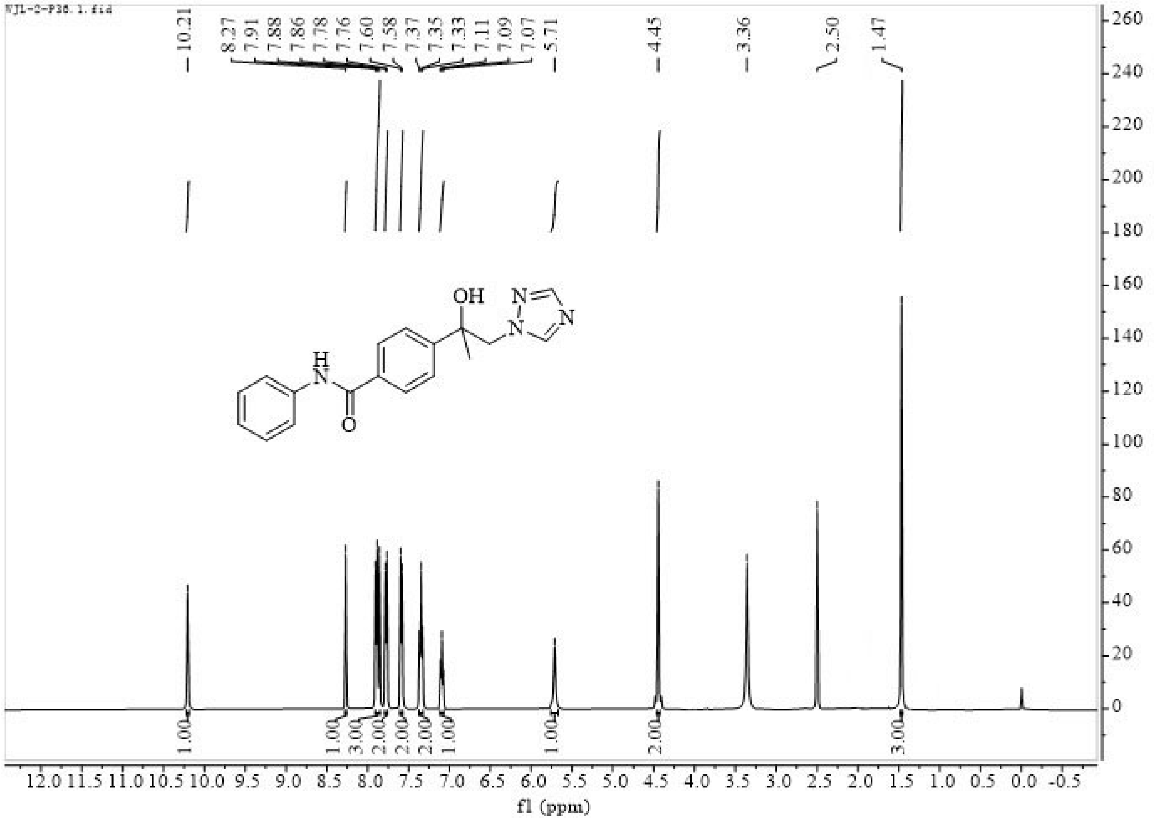

Figure S7. ^{1}H NMR spectrum of **5a**

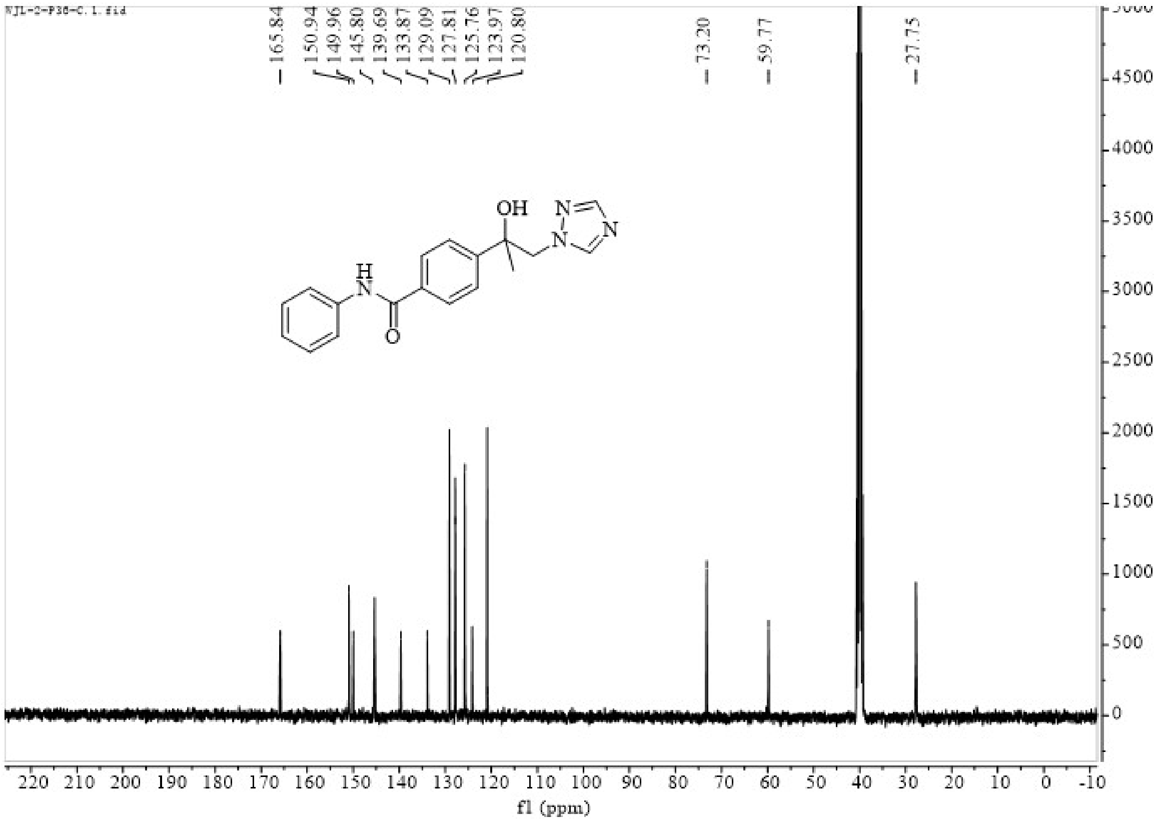

Figure S8. ^{13}C NMR spectrum of **5a**

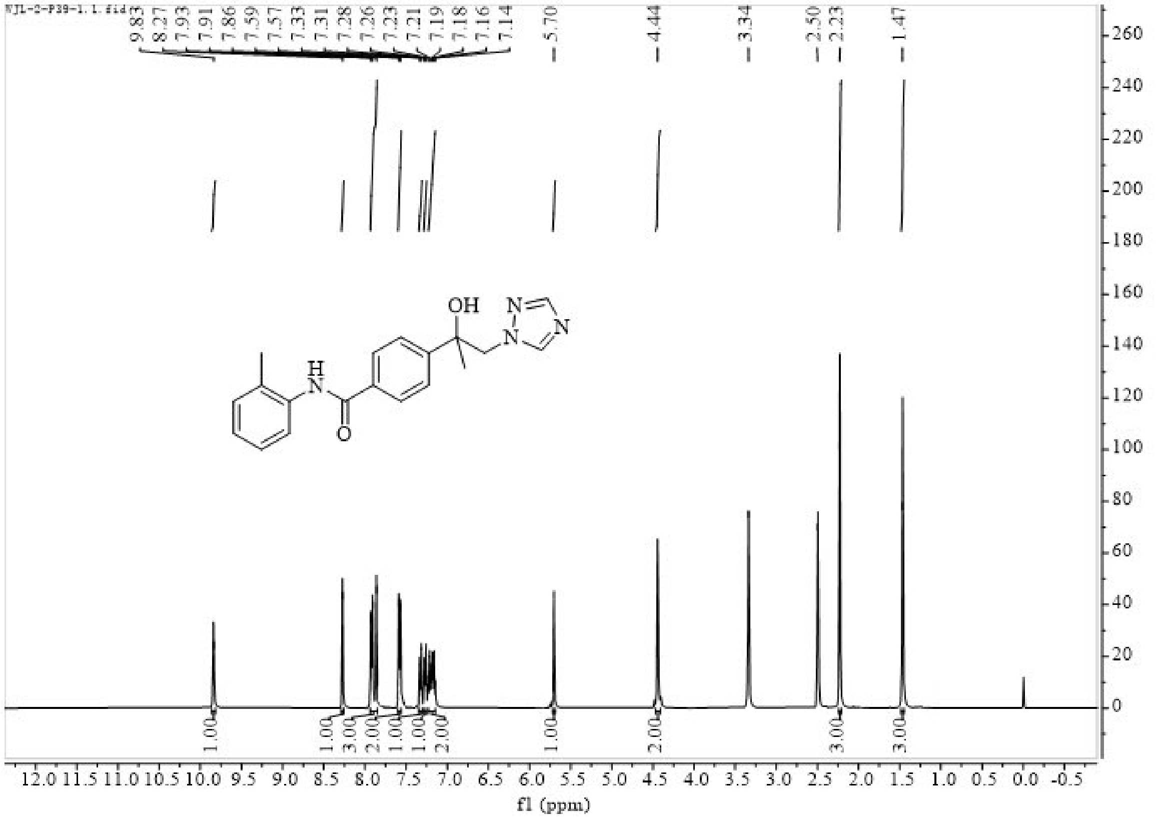

Figure S9. ^{1}H NMR spectrum of **5b**

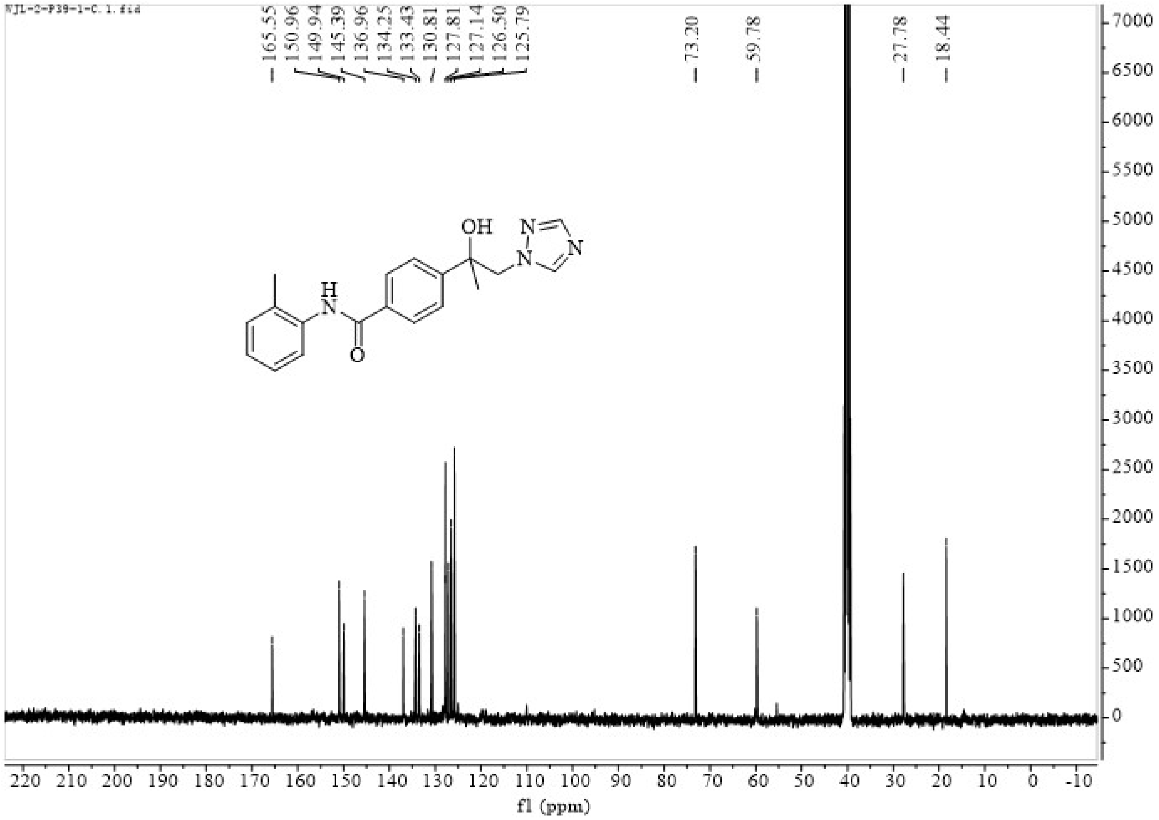

Figure S10. ^{13}C NMR spectrum of **5b**

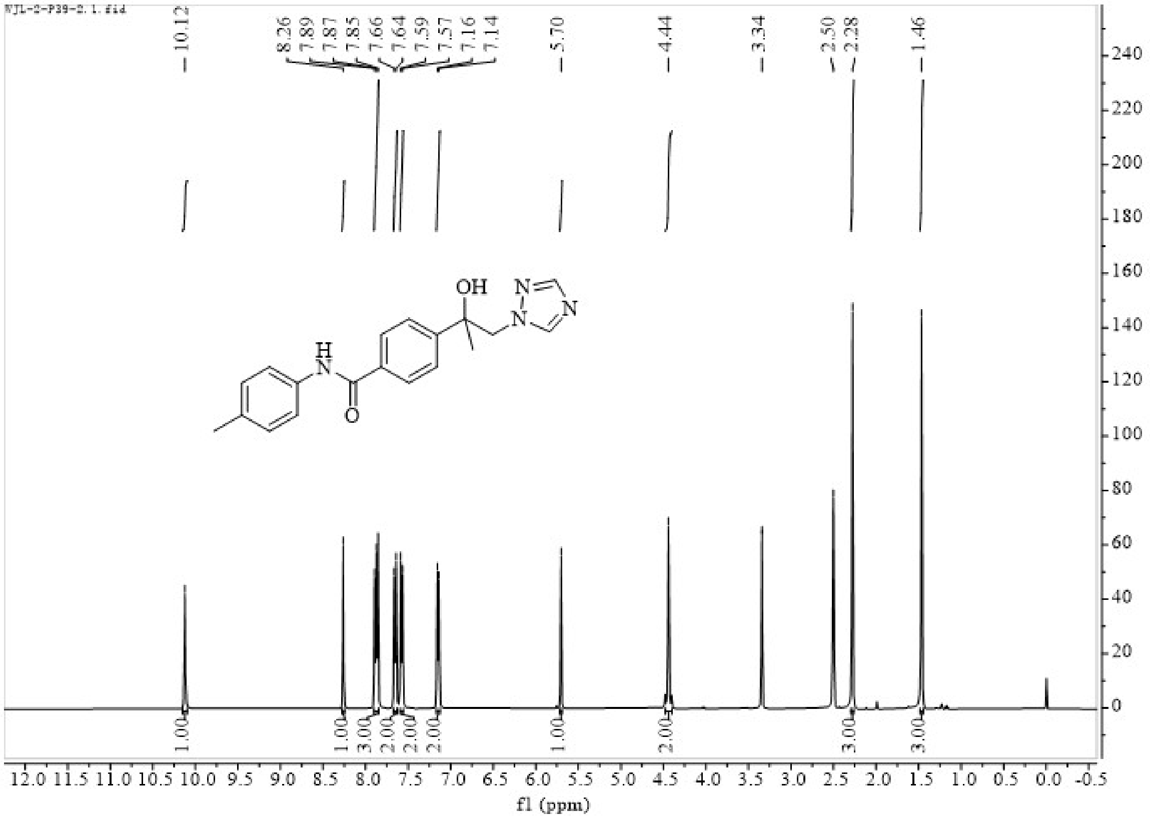

Figure S11. ^{1}H NMR spectrum of **5c**

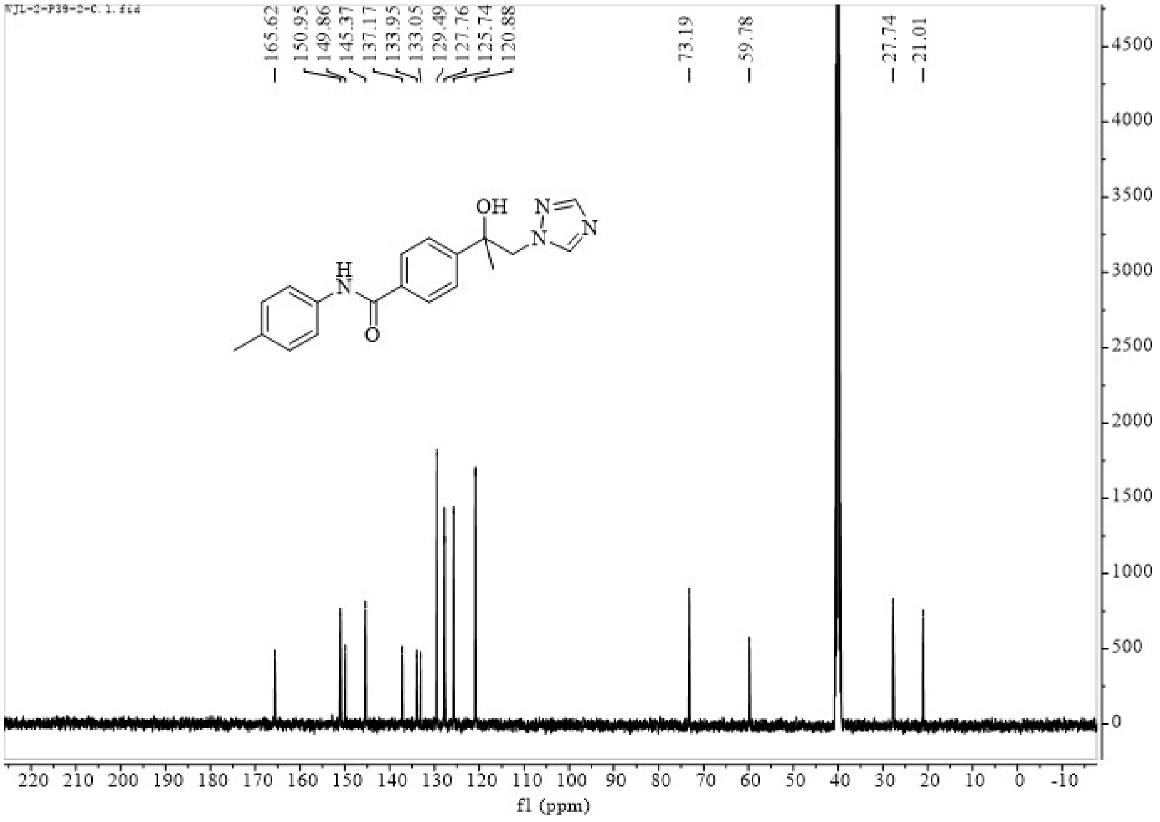

Figure S12. ^{13}C NMR spectrum of **5c**

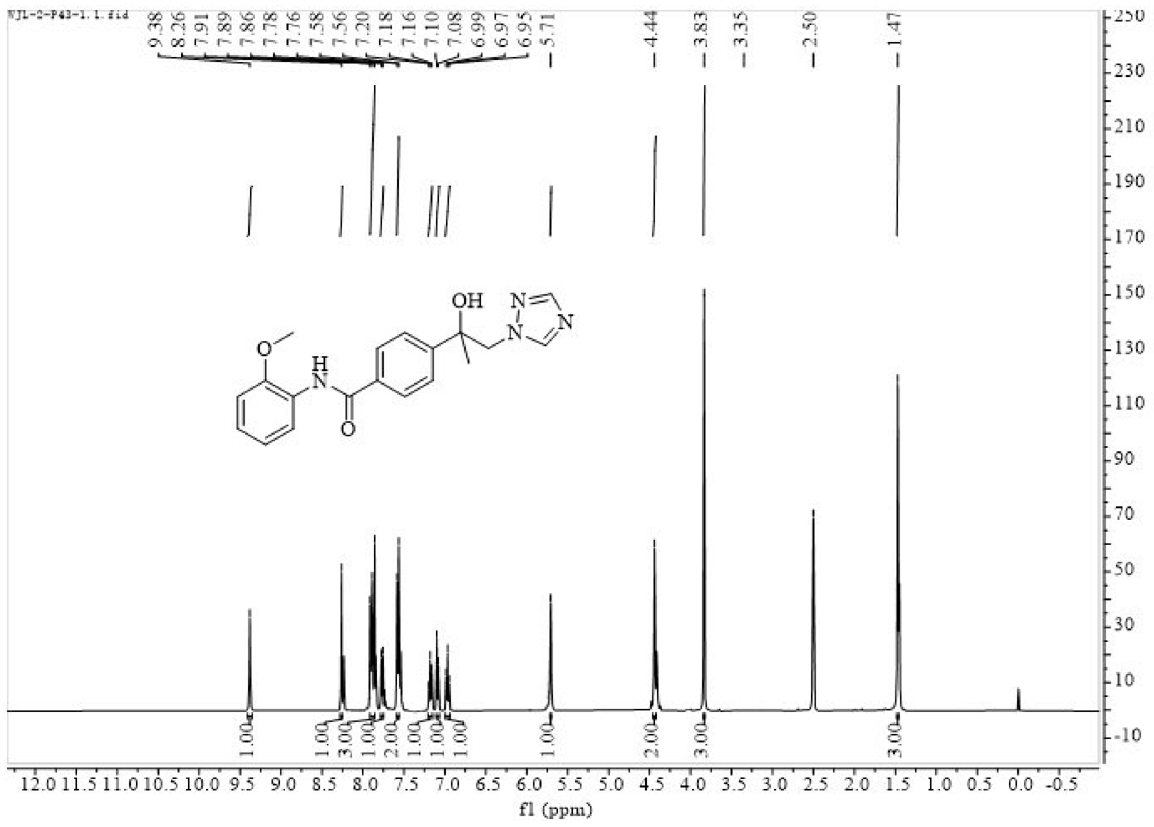

Figure S13. ^{1}H NMR spectrum of **5d**

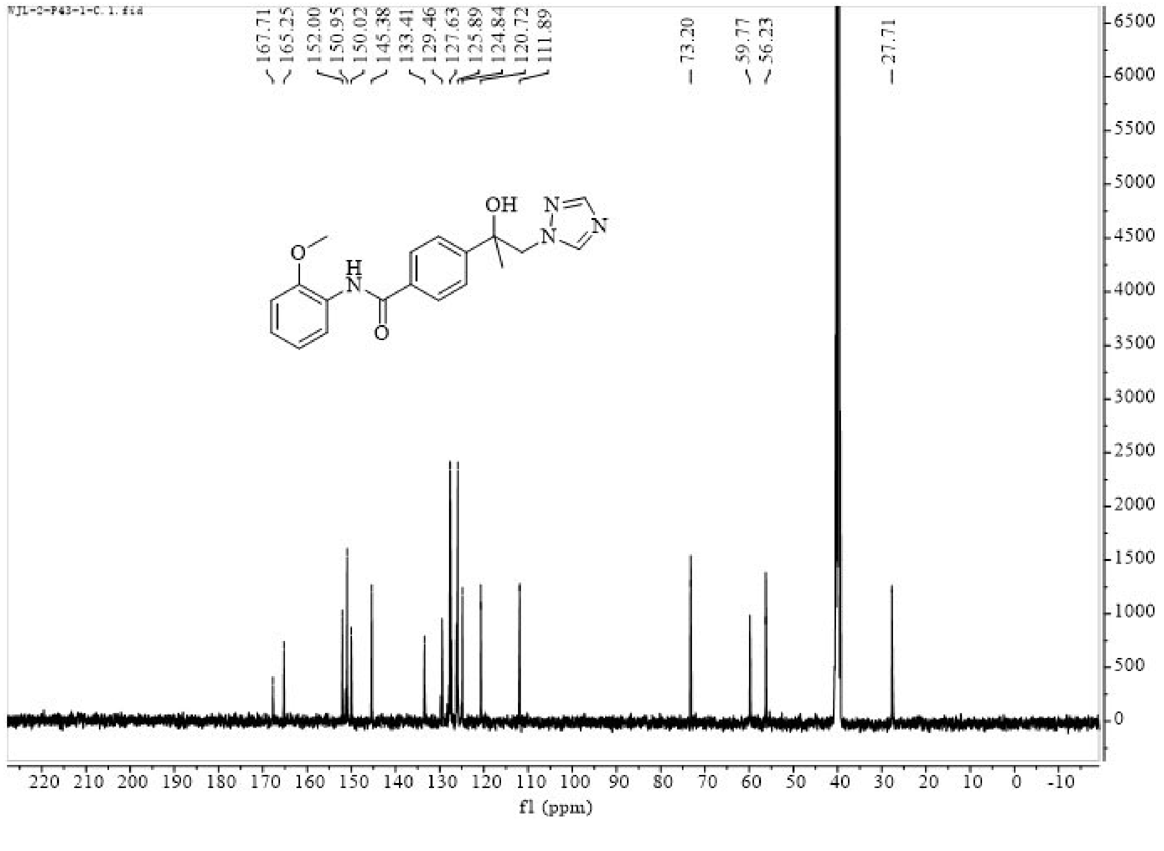

Figure S14. ^{13}C NMR spectrum of **5d**

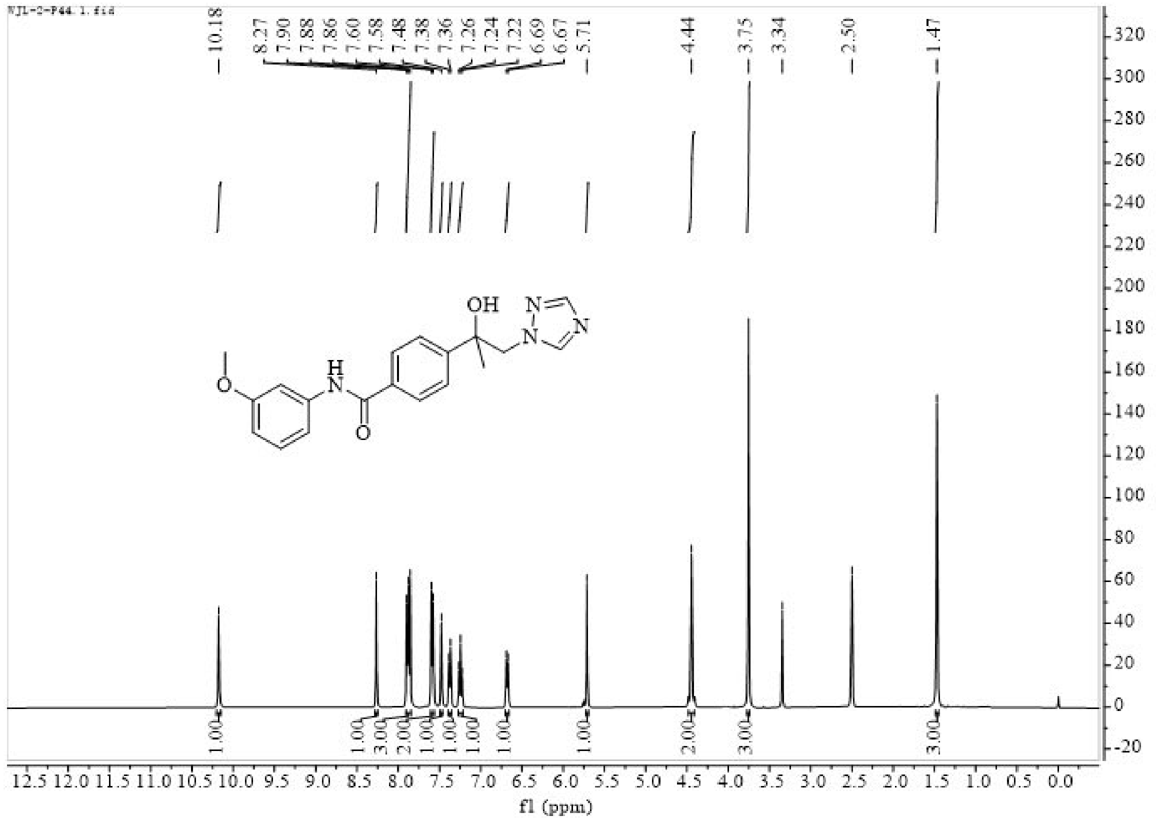

Figure S15. ^{1}H NMR spectrum of **5e**

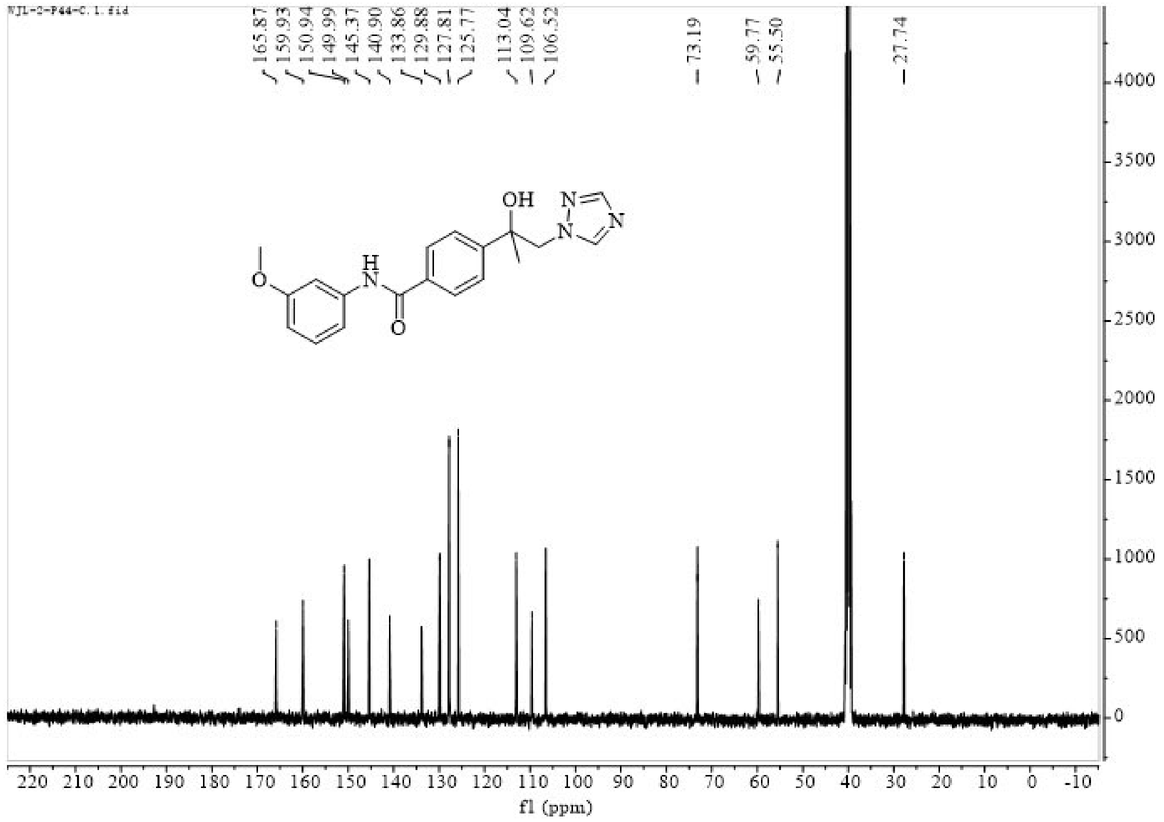

Figure S16. ^{13}C NMR spectrum of **5e**

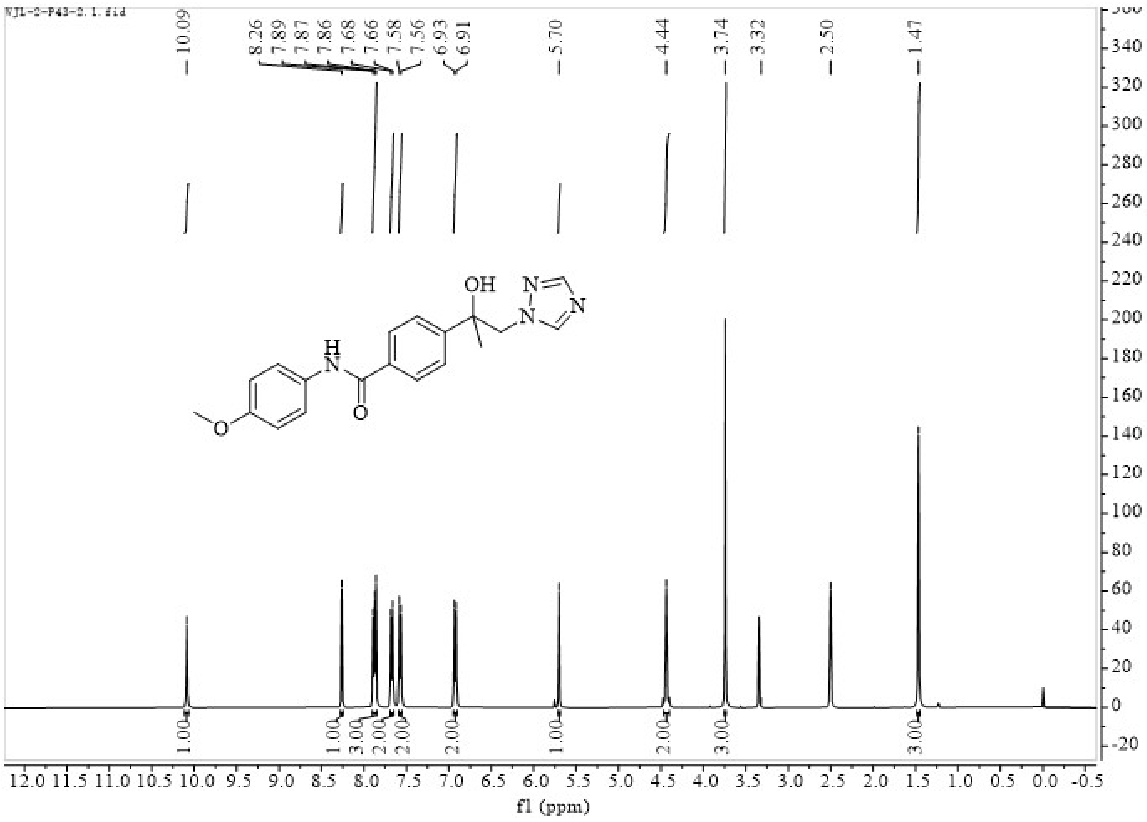

Figure S17. ^{1}H NMR spectrum of **5f**

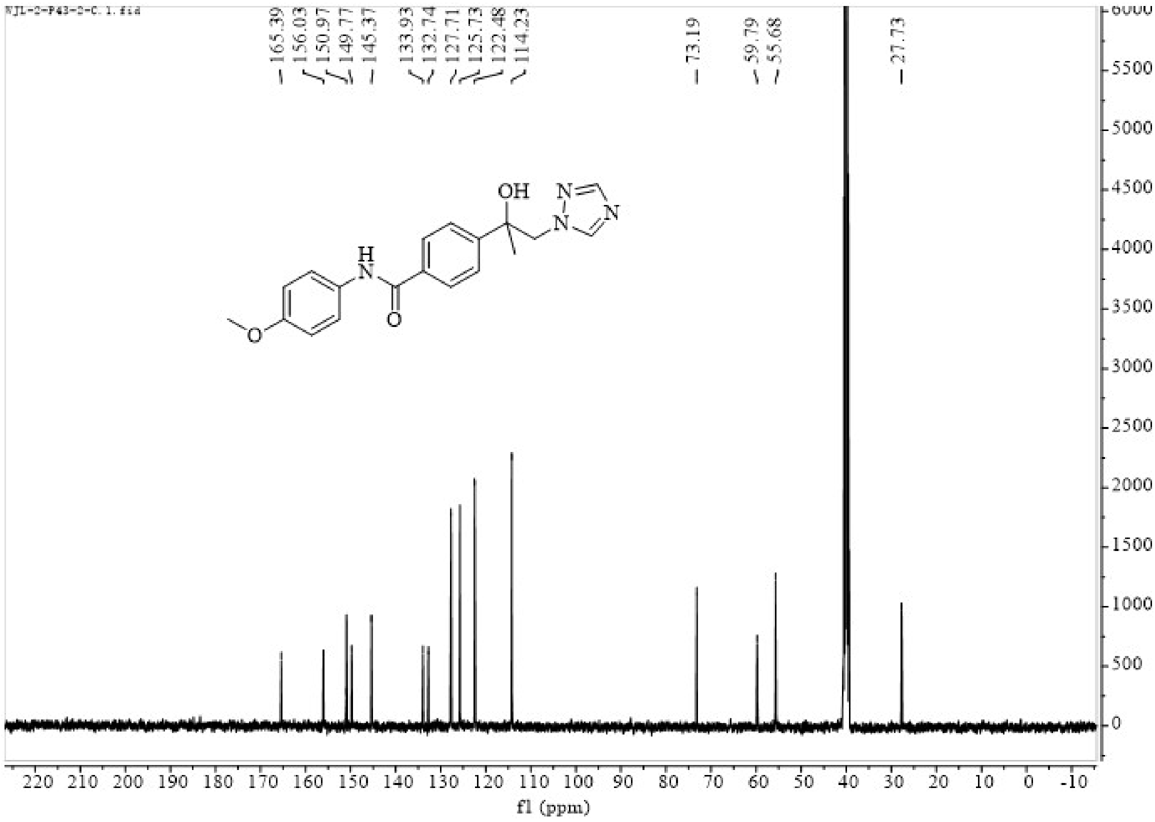

Figure S18. ^{13}C NMR spectrum of **5f**

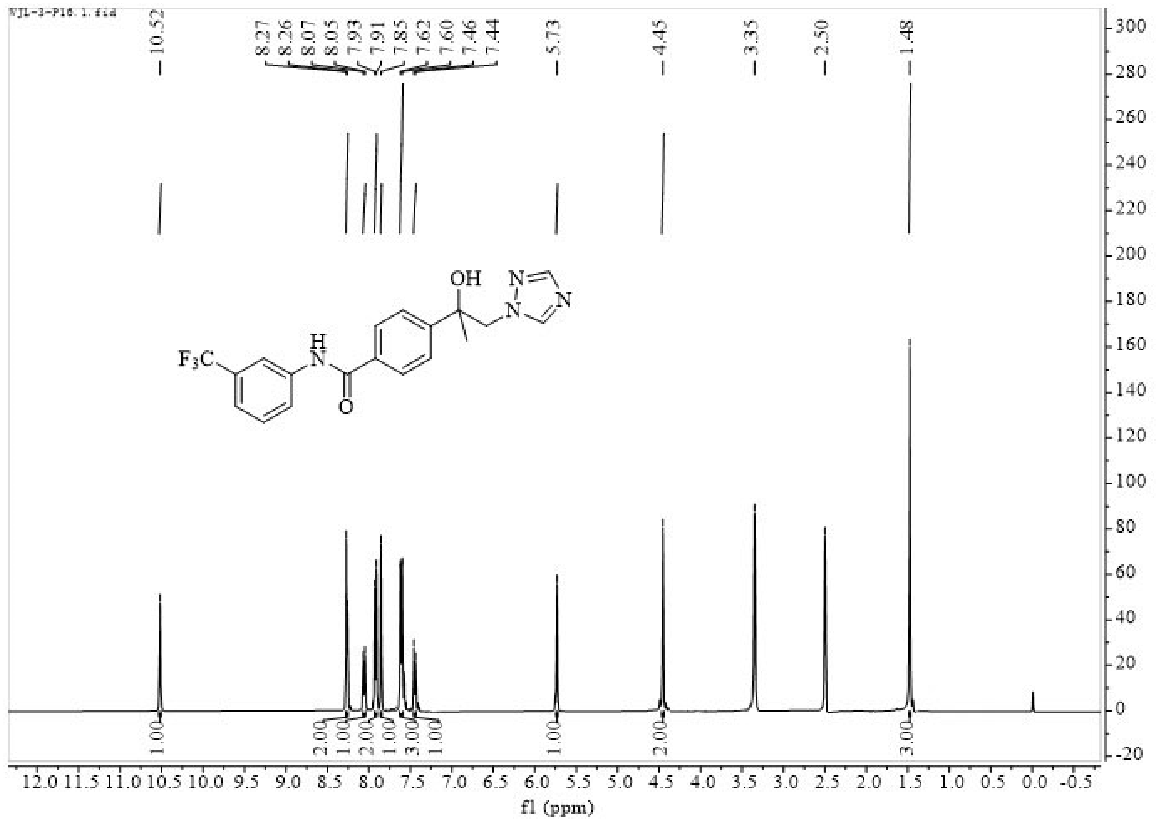

Figure S19. ^{1}H NMR spectrum of **5g**

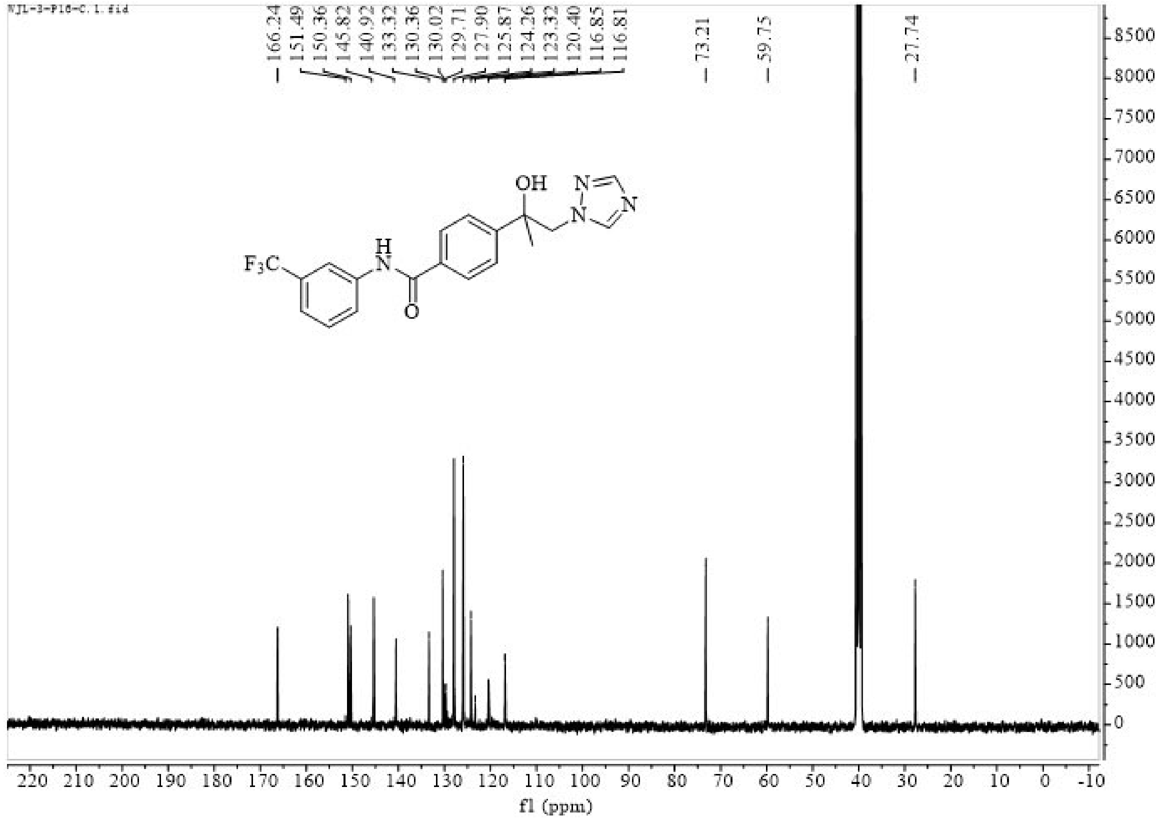

Figure S20. ^{13}C NMR spectrum of **5g**

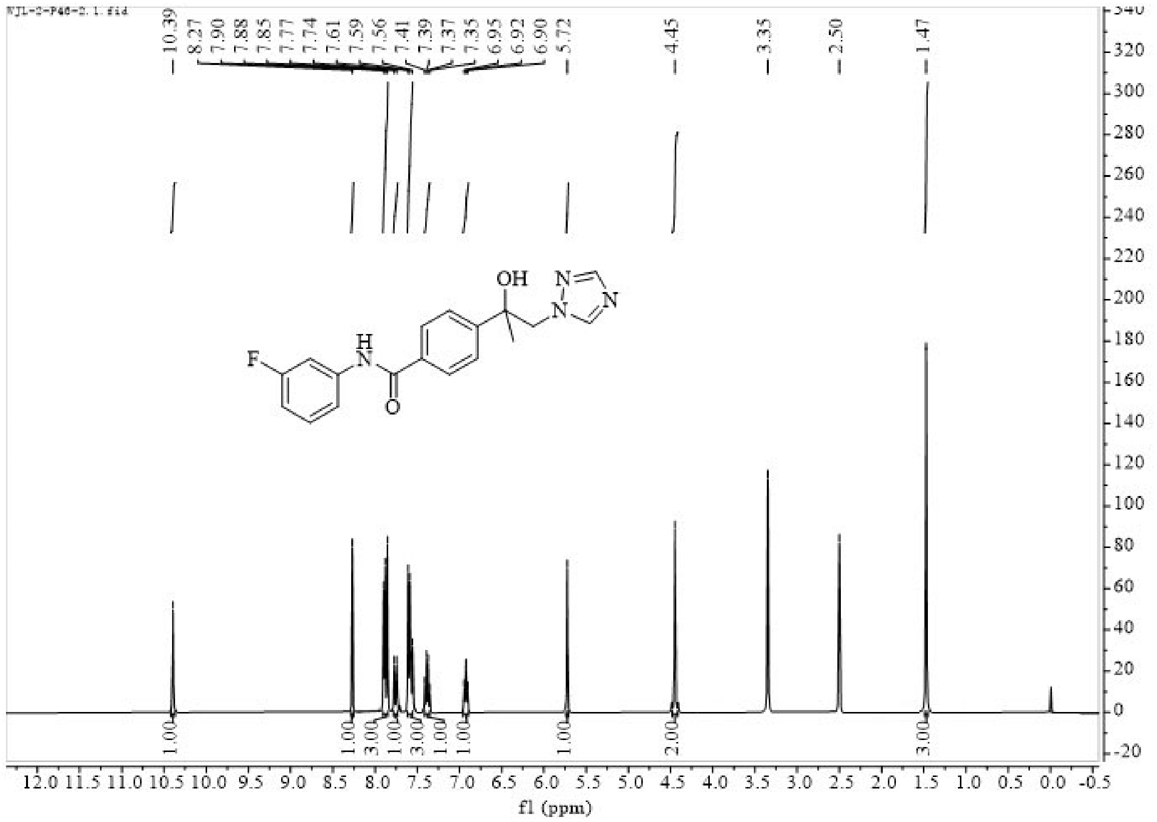

Figure S21. ^{1}H NMR spectrum of **5h**

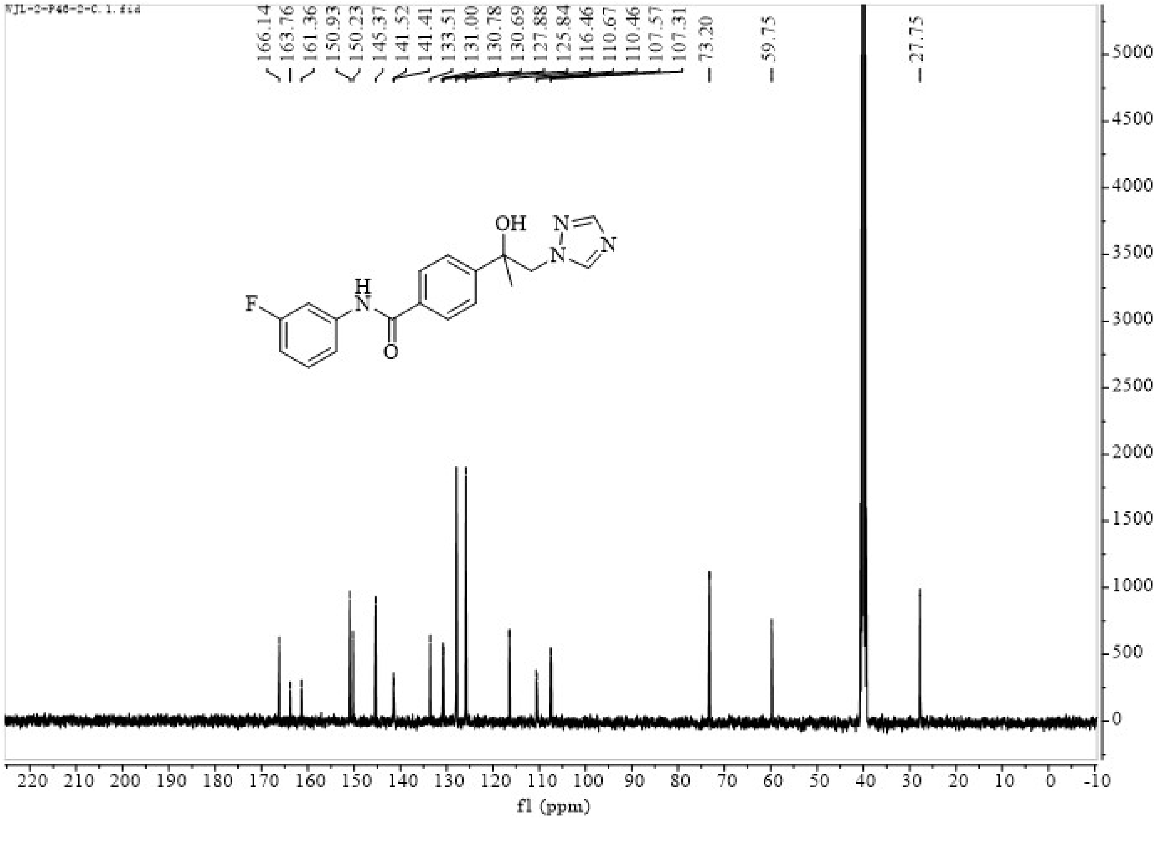

Figure S22. ^{13}C NMR spectrum of **5h**

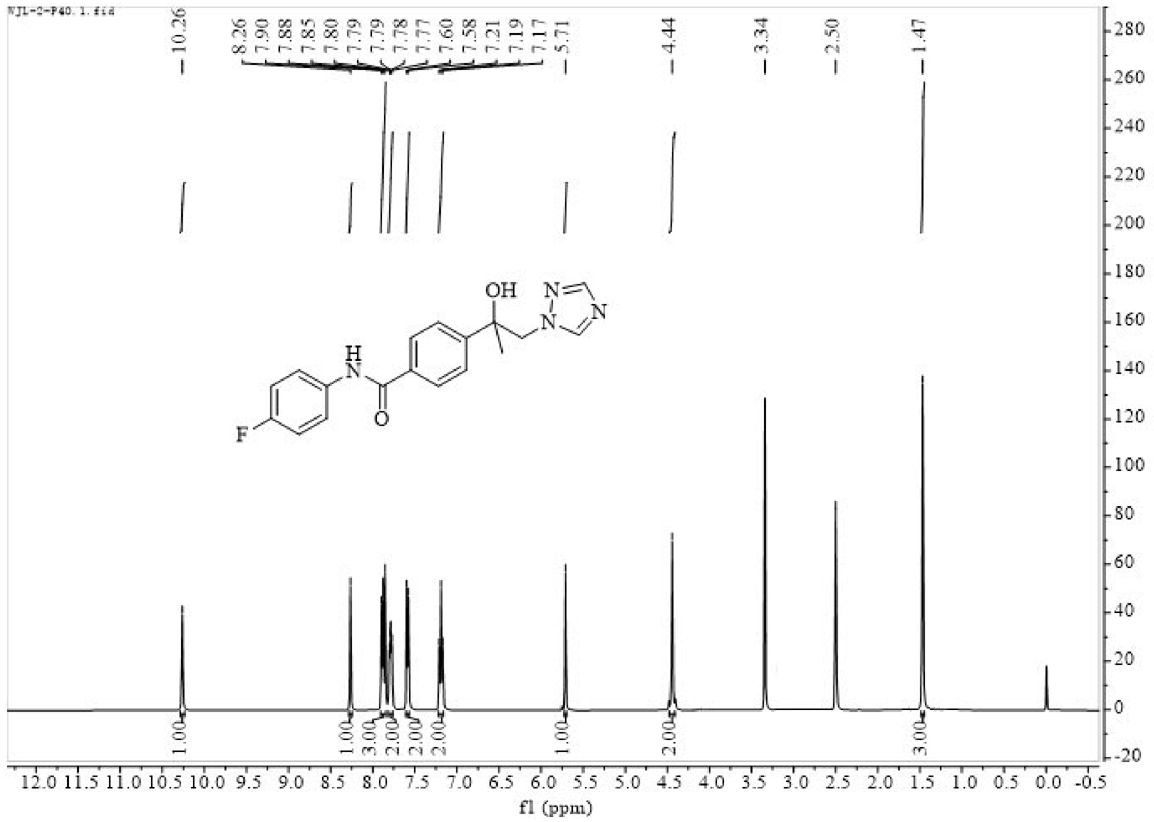

Figure S23. ^{1}H NMR spectrum of **5i**

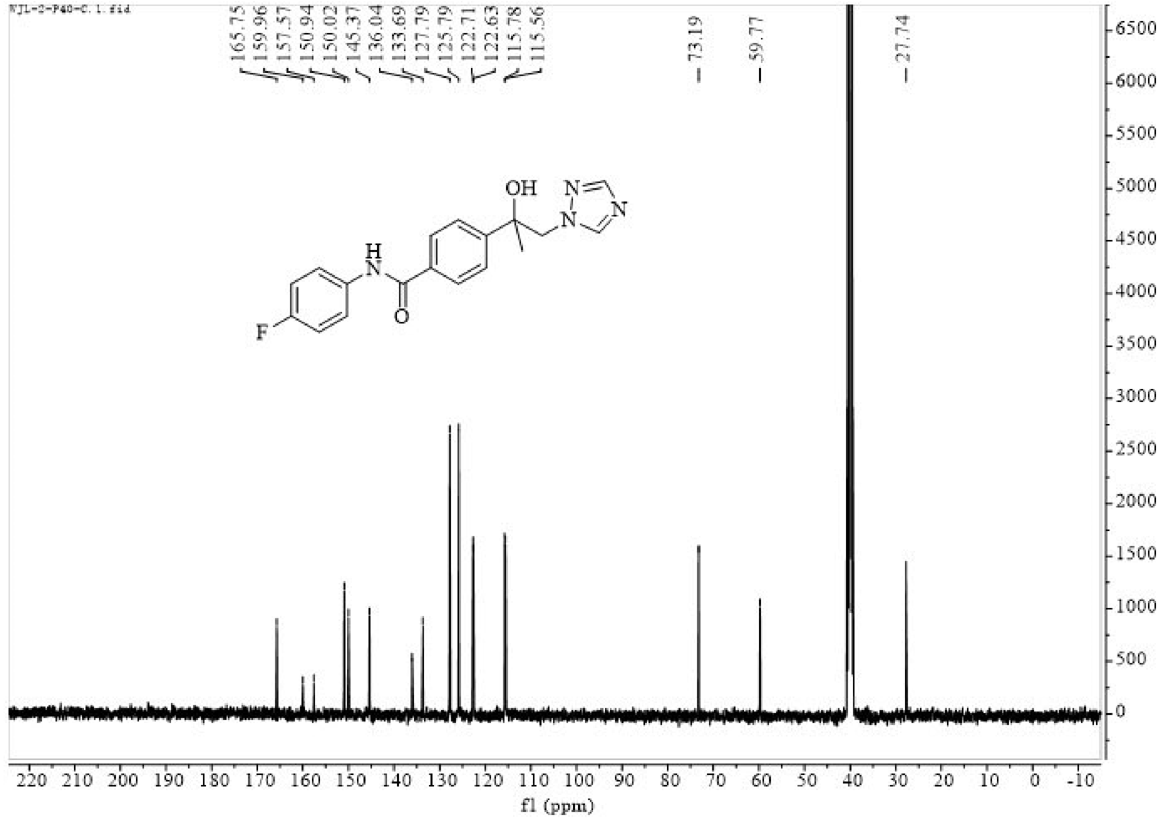

Figure S24. ^{13}C NMR spectrum of **5i**

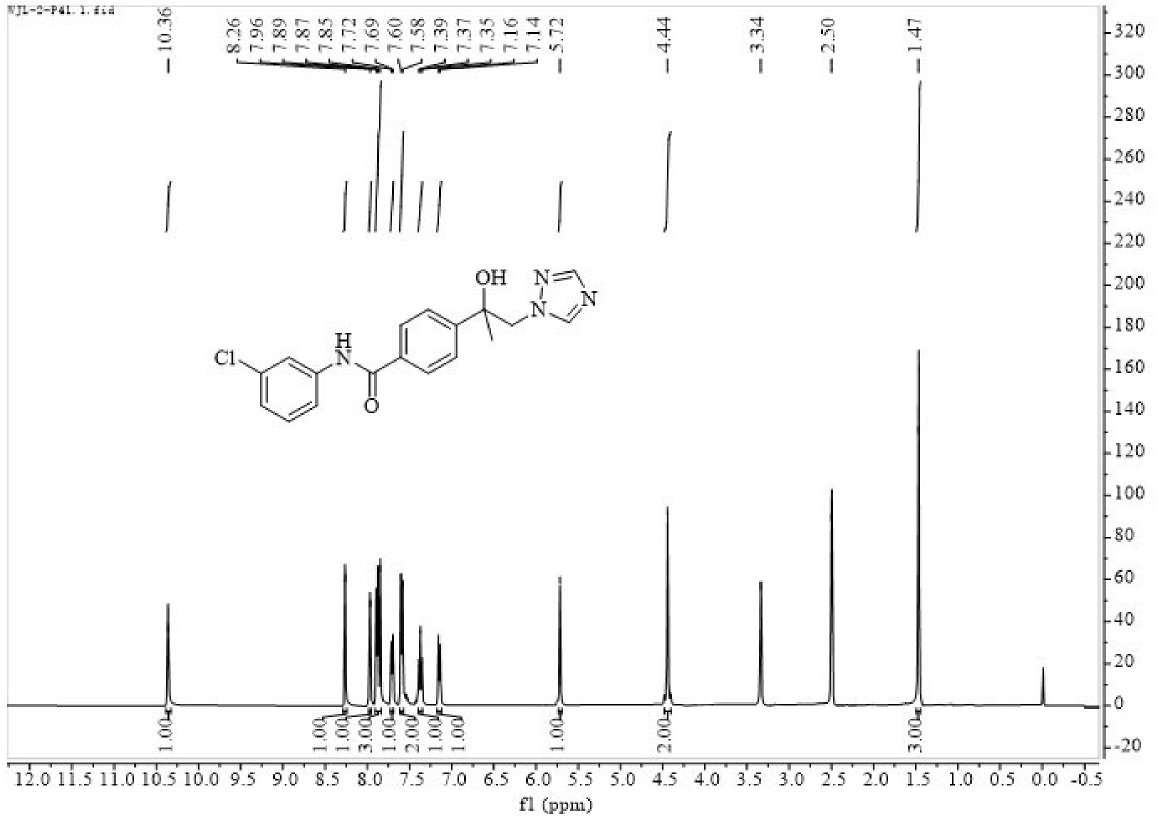

Figure S25. ^{1}H NMR spectrum of **5j**

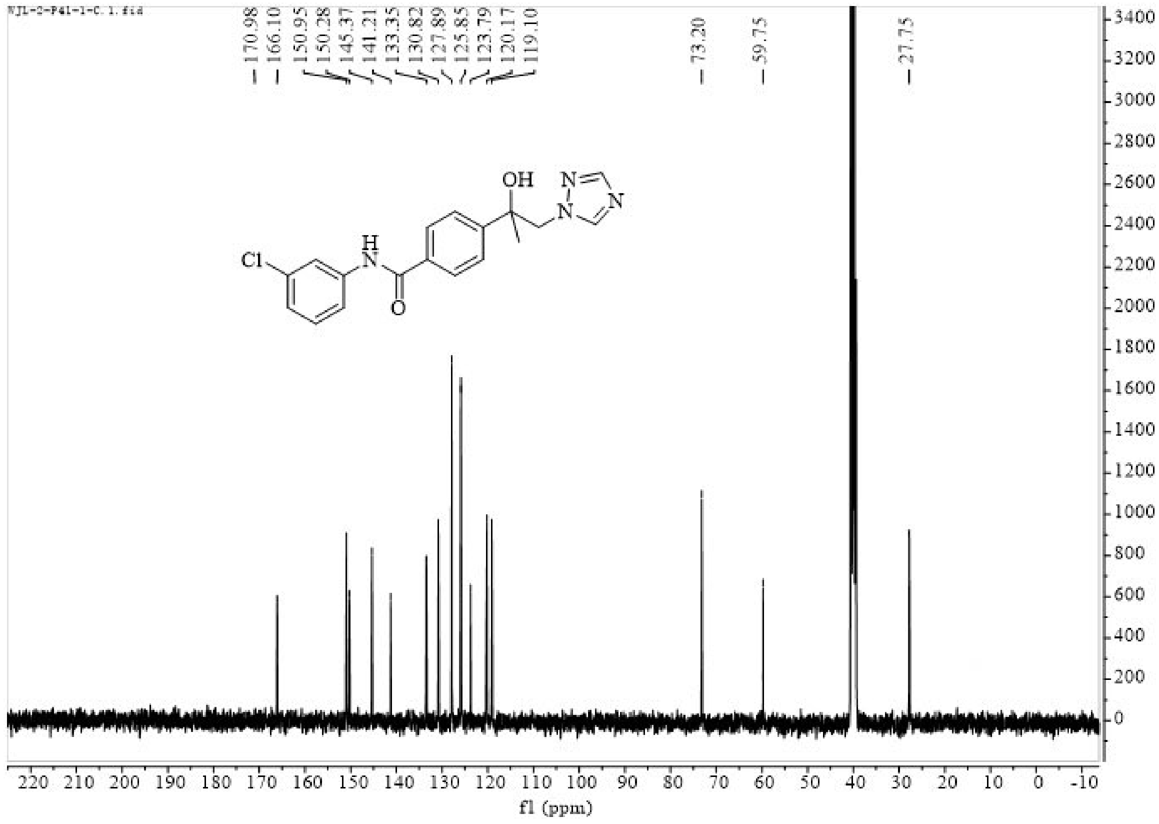

Figure S26. ^{13}C NMR spectrum of **5j**

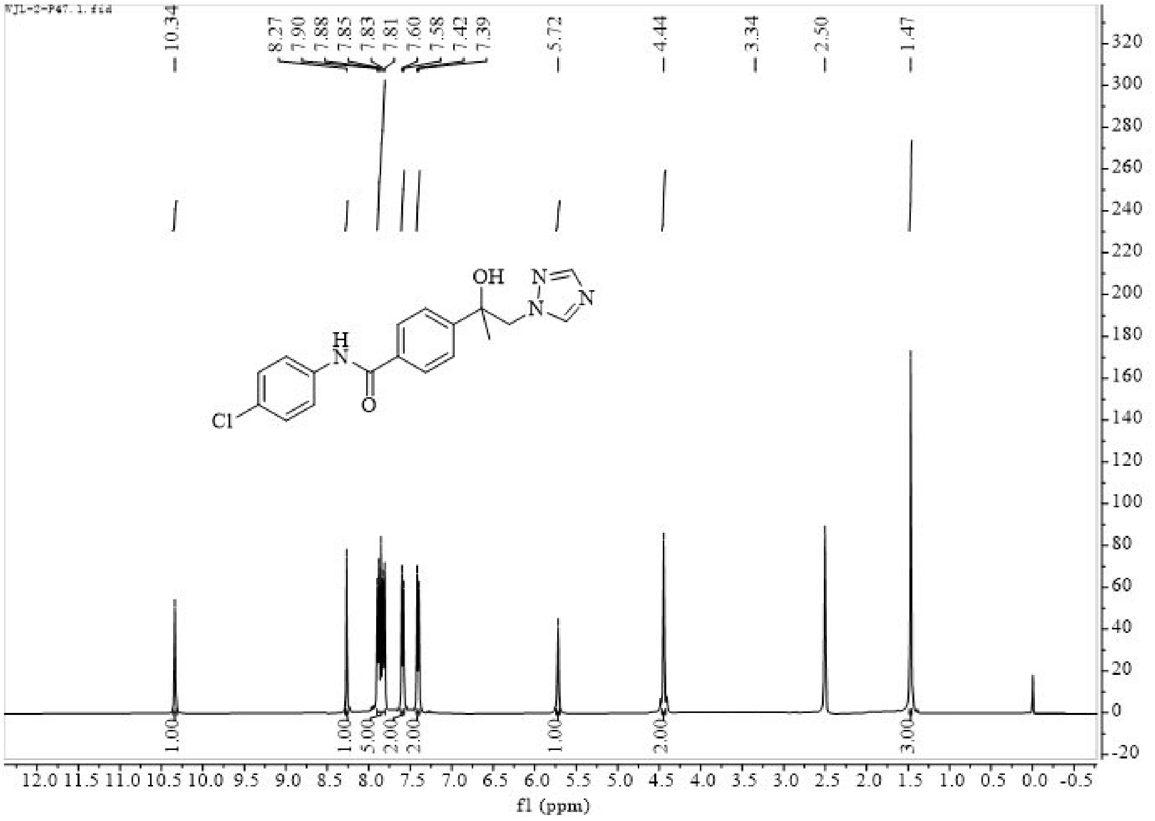

Figure S27. ^{1}H NMR spectrum of **5k**

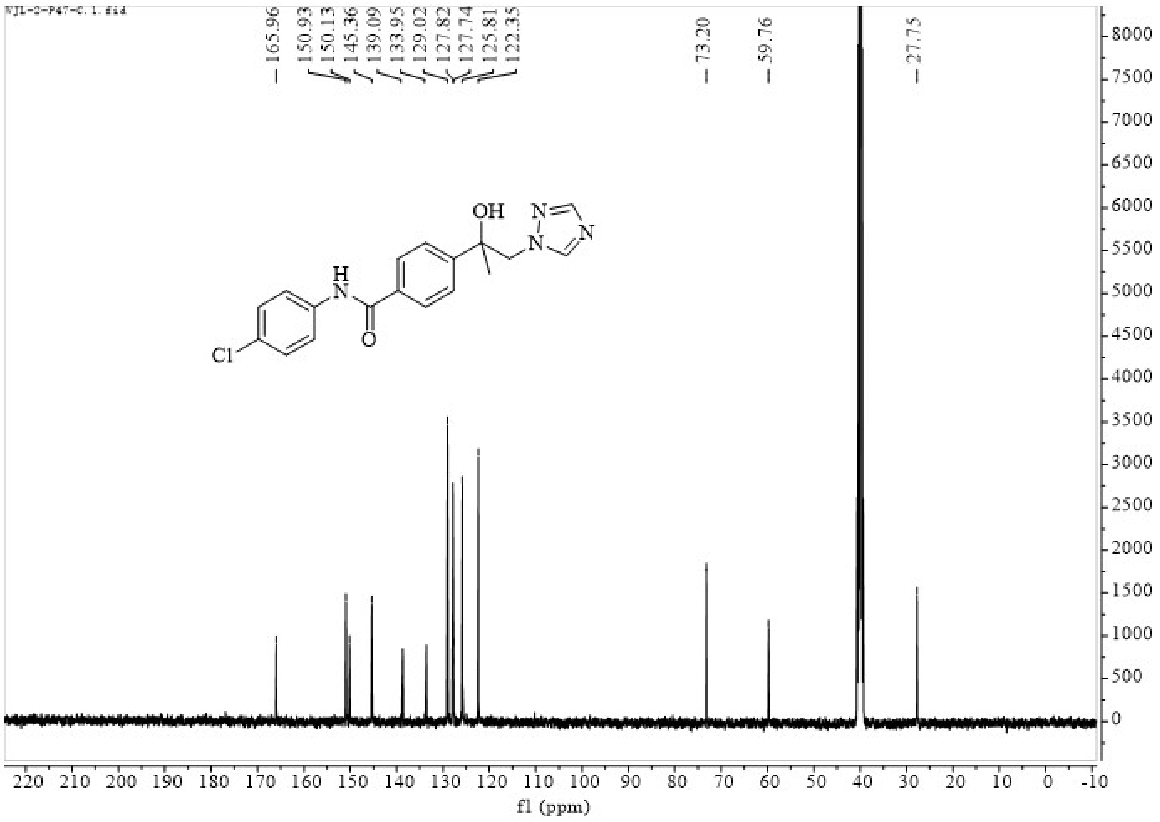

Figure S28. ^{13}C NMR spectrum of **5k**

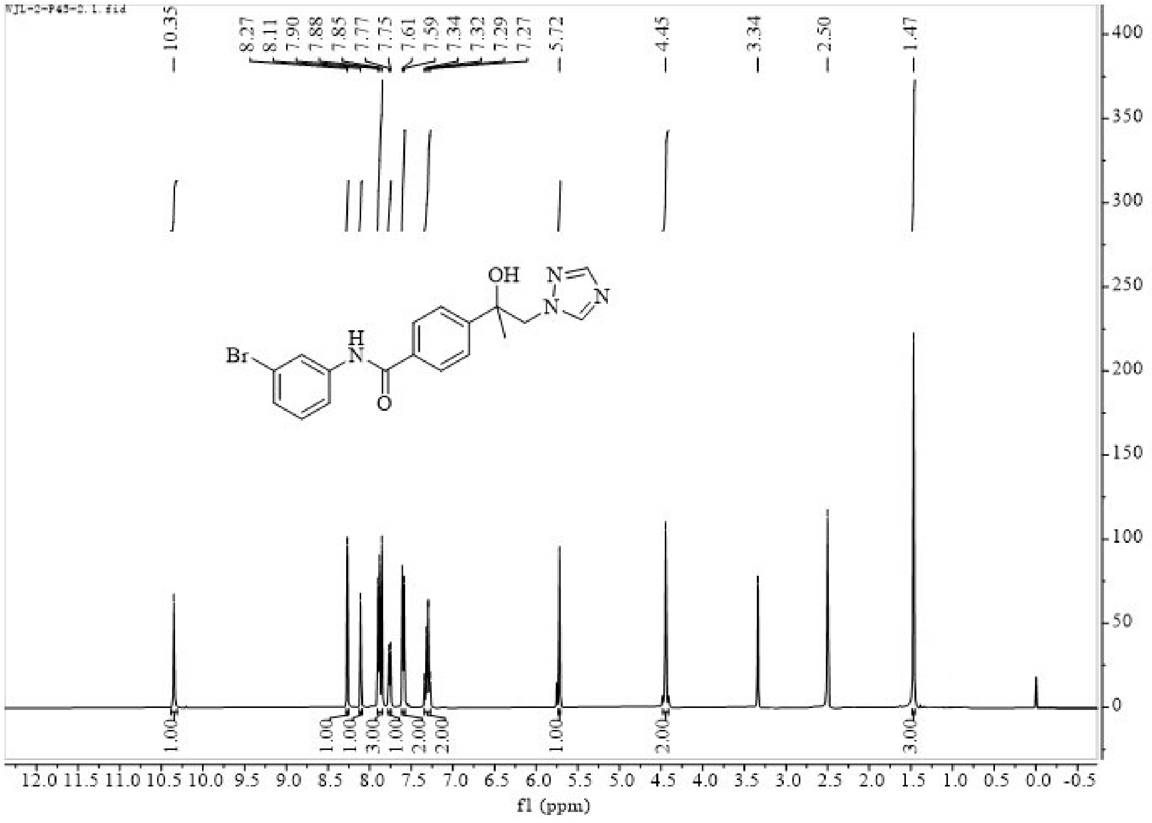

Figure S29. ^{1}H NMR spectrum of **5l**

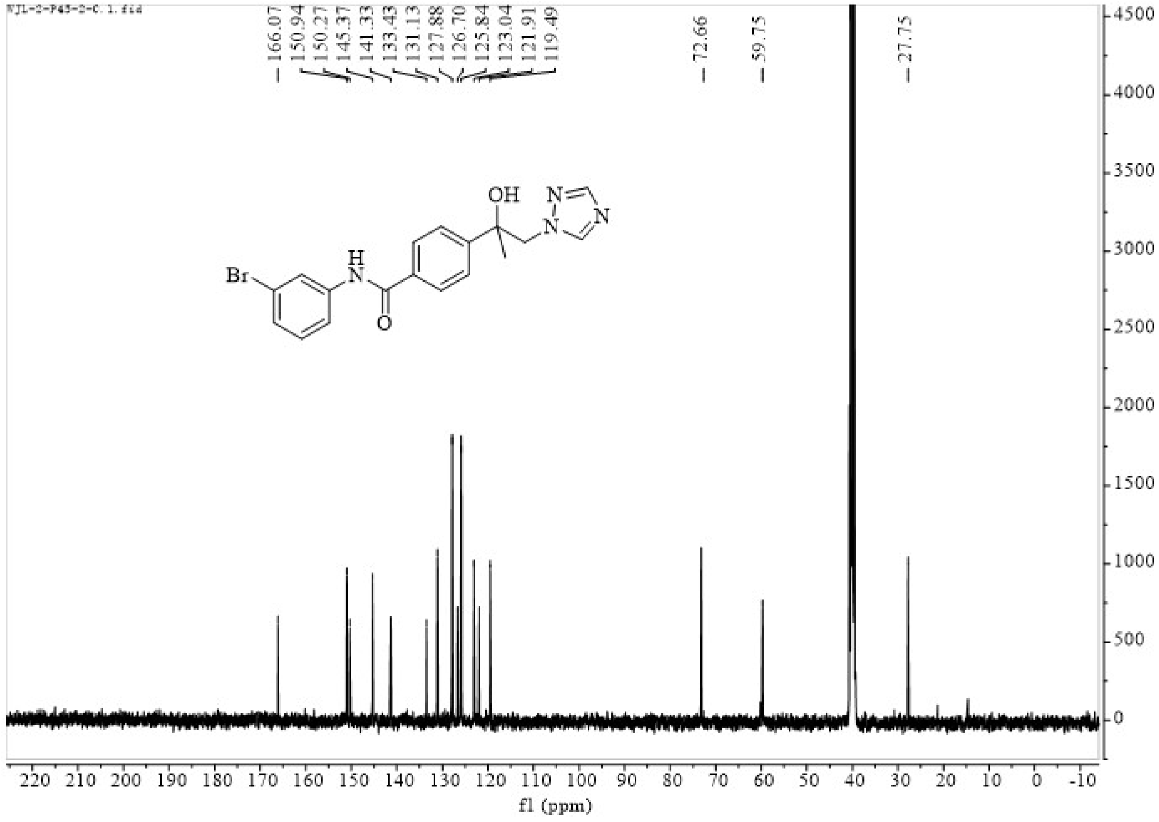

Figure S30. ^{13}C NMR spectrum of **5l**

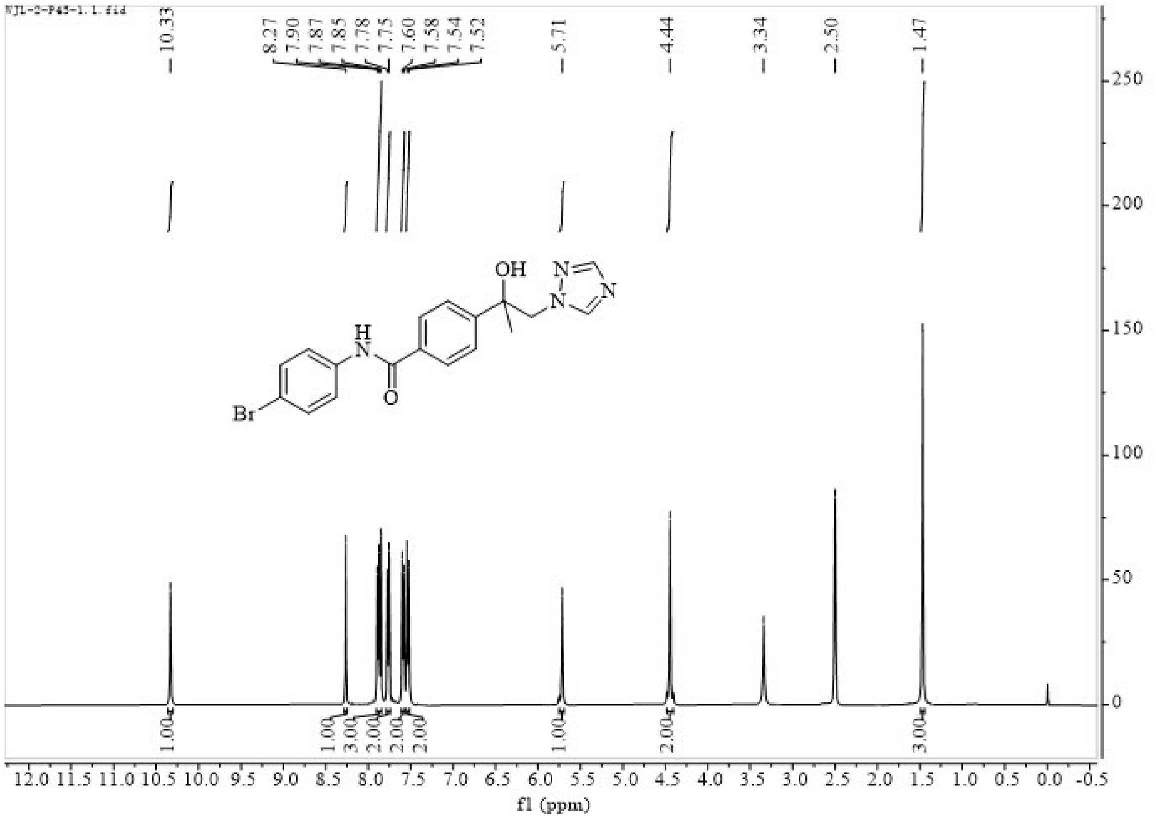

Figure S31. ^{1}H NMR spectrum of **5m**

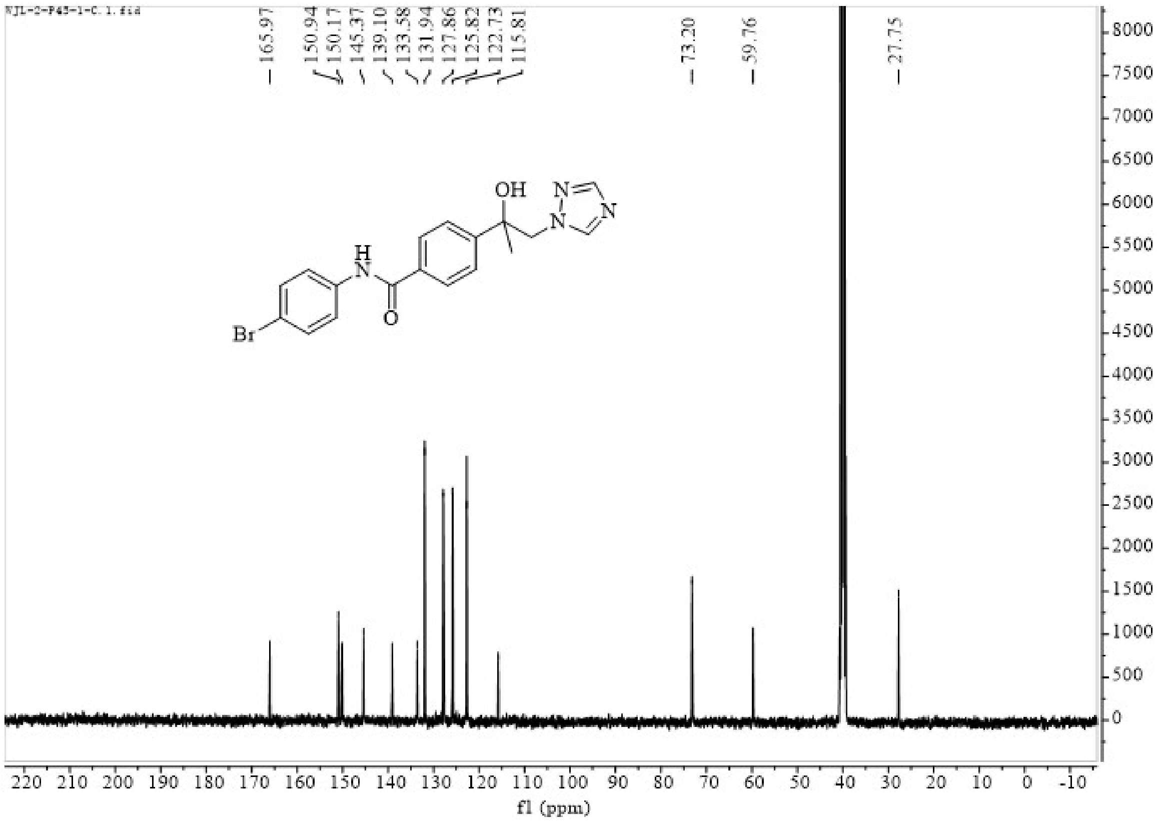

Figure S32. ^{13}C NMR spectrum of **5m**

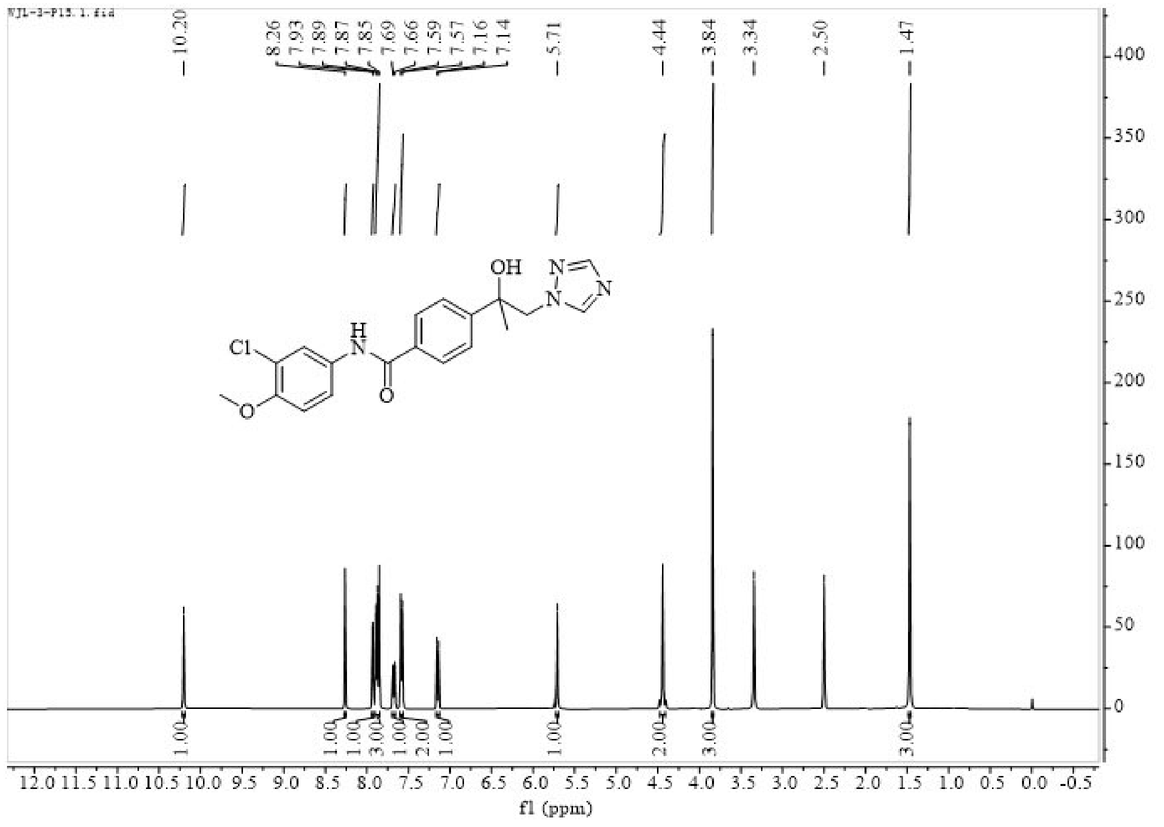

Figure S33. ^{1}H NMR spectrum of **5n**

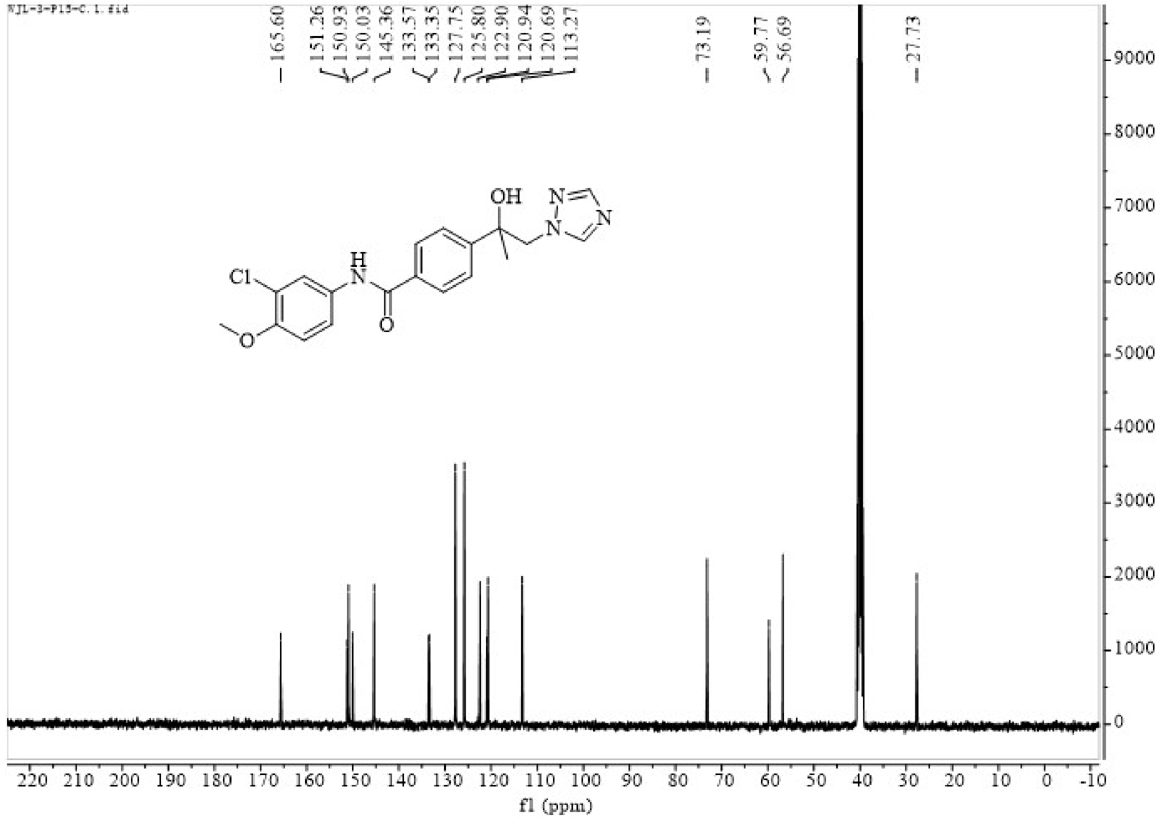

Figure S34. ^{13}C NMR spectrum of **5n**

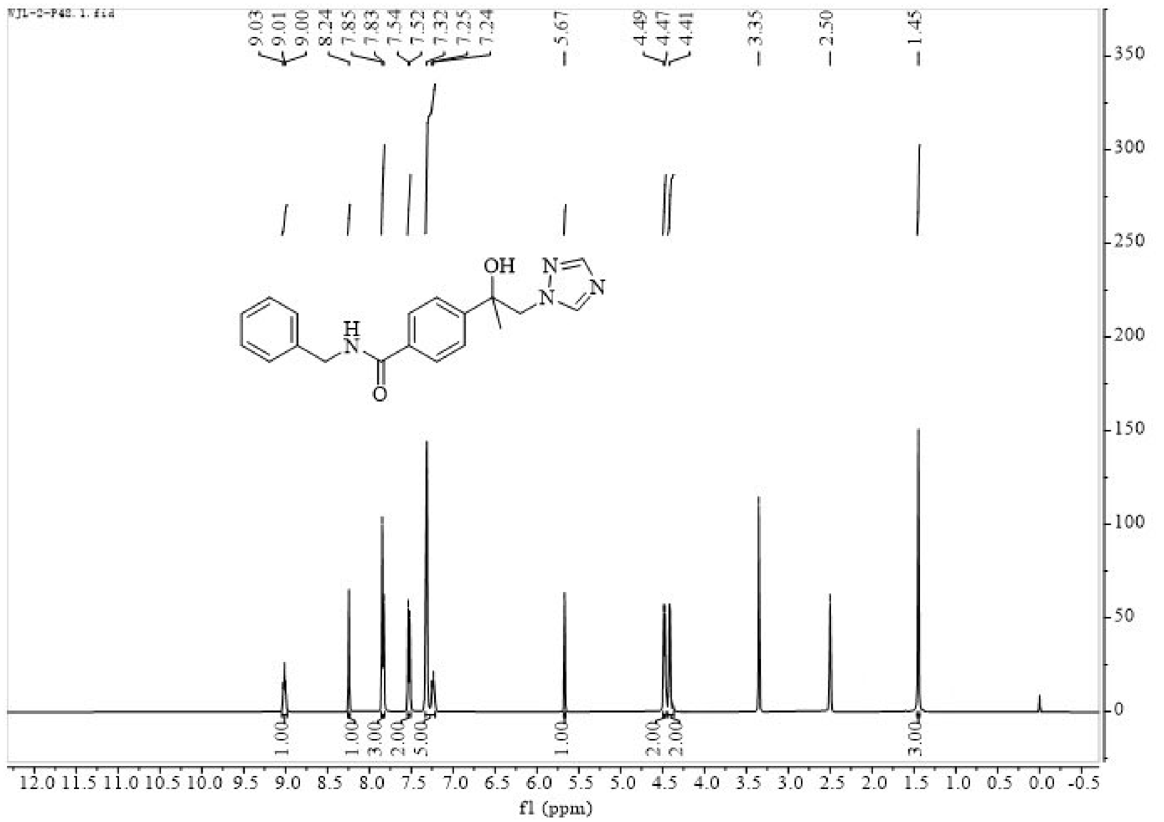

Figure S35. ^{1}H NMR spectrum of **6a**

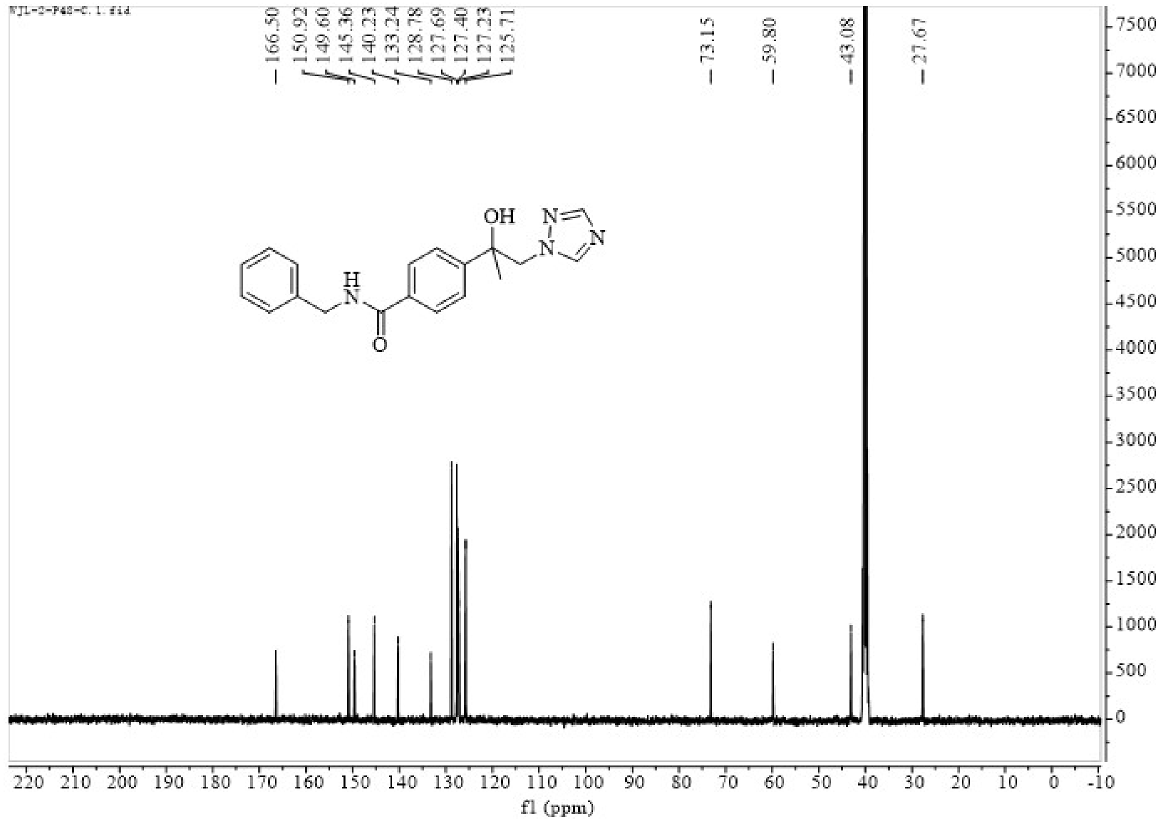

Figure S36. ^{13}C NMR spectrum of **6a**

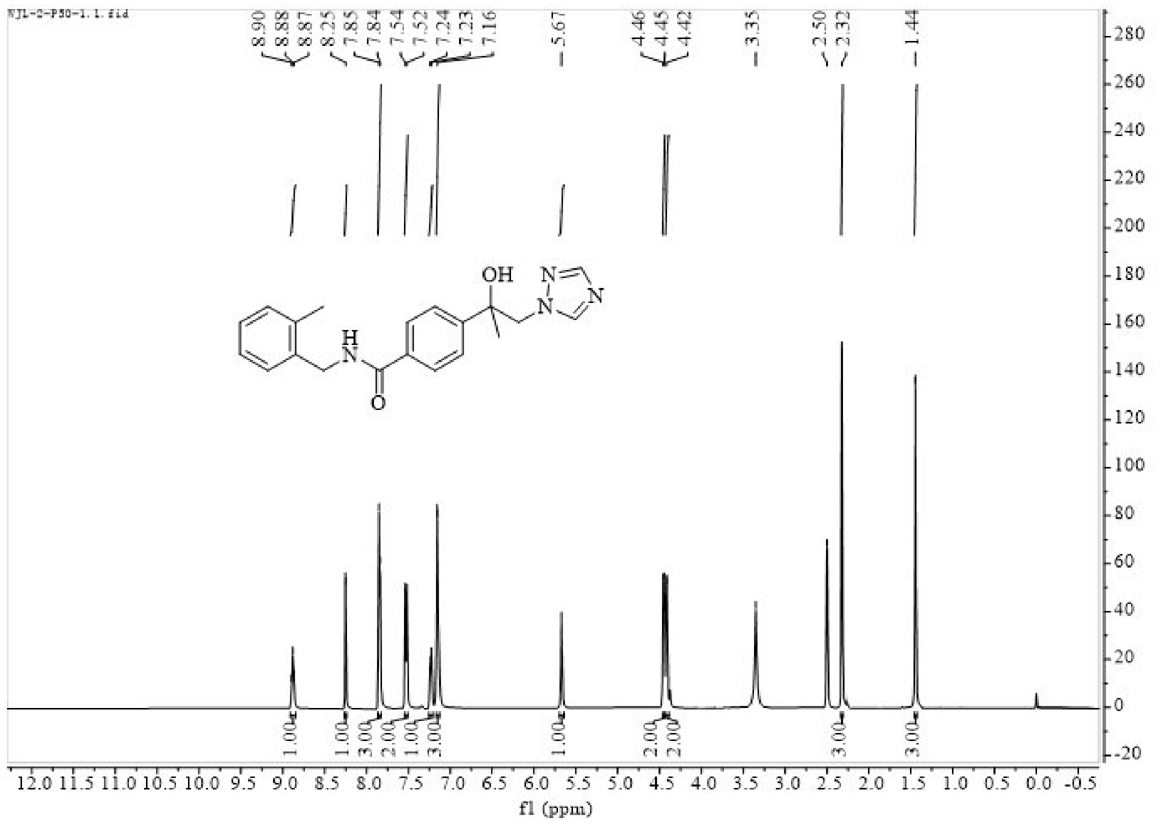

Figure S37. ^{1}H NMR spectrum of **6b**

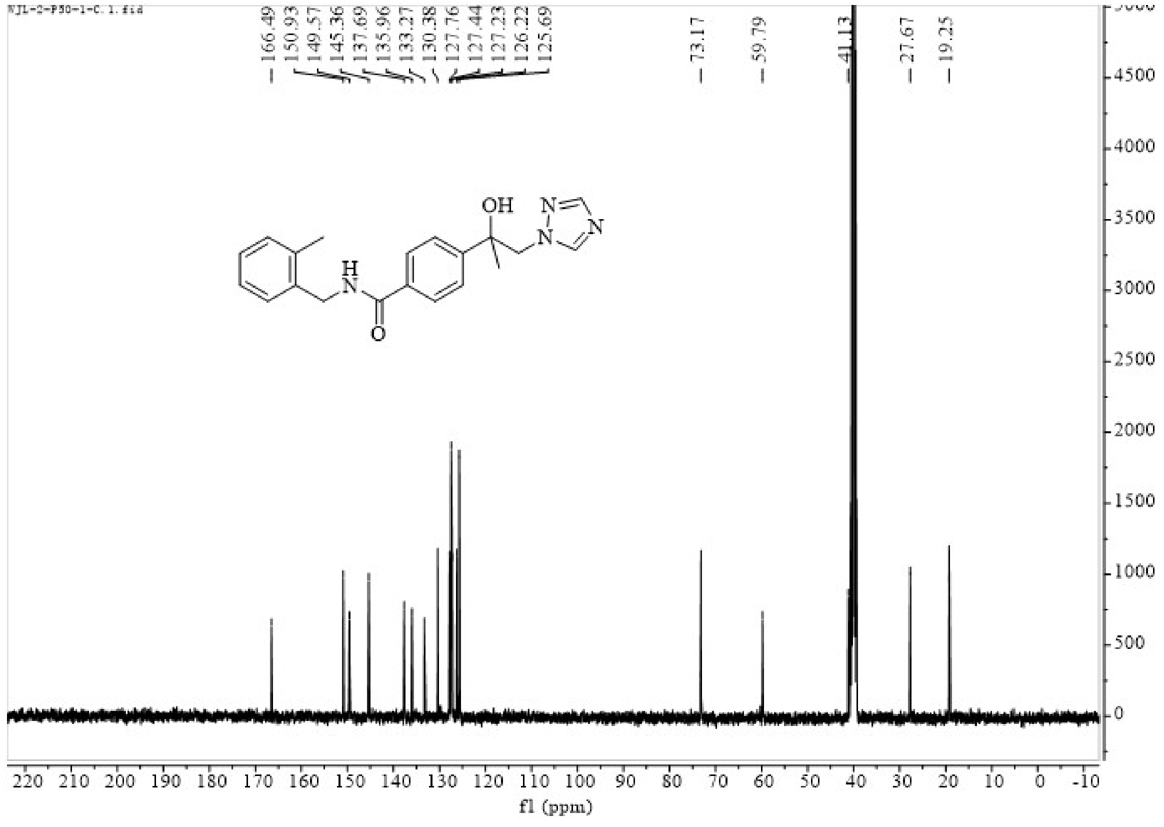

Figure S38. ^{13}C NMR spectrum of **6b**

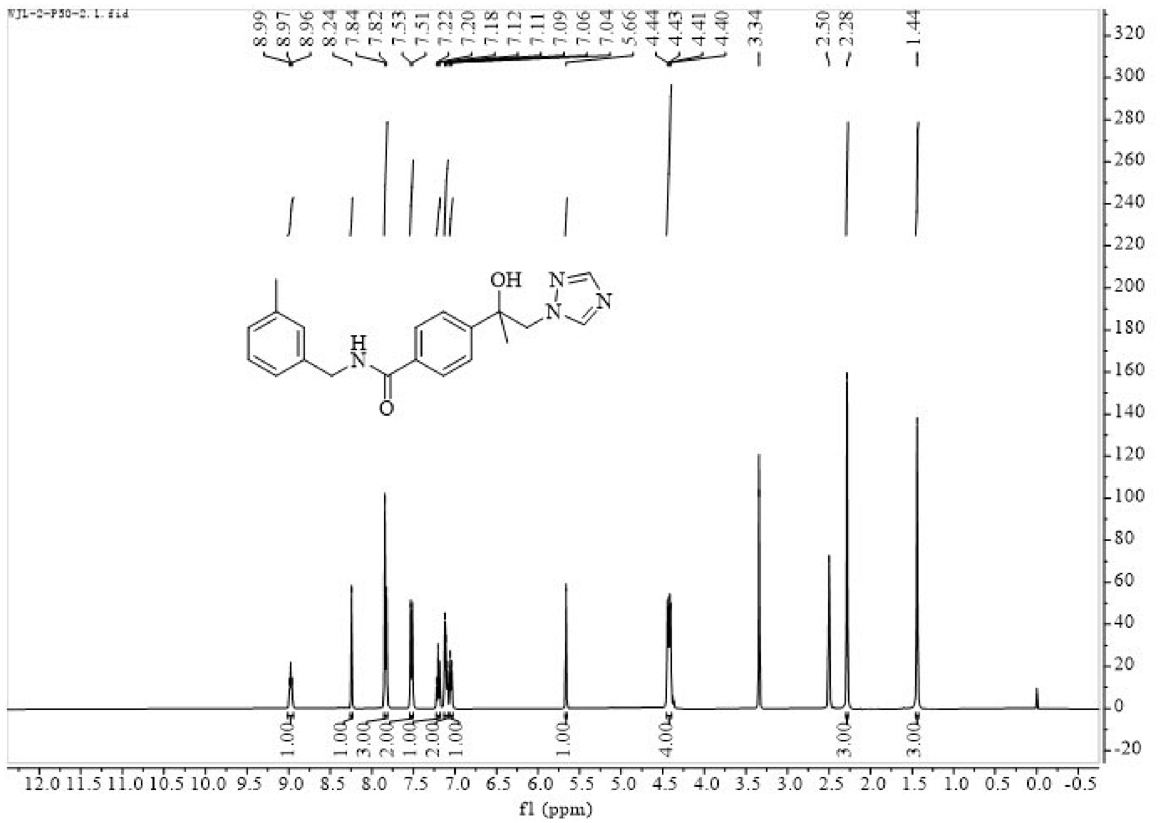

Figure S39. ^{1}H NMR spectrum of **6c**

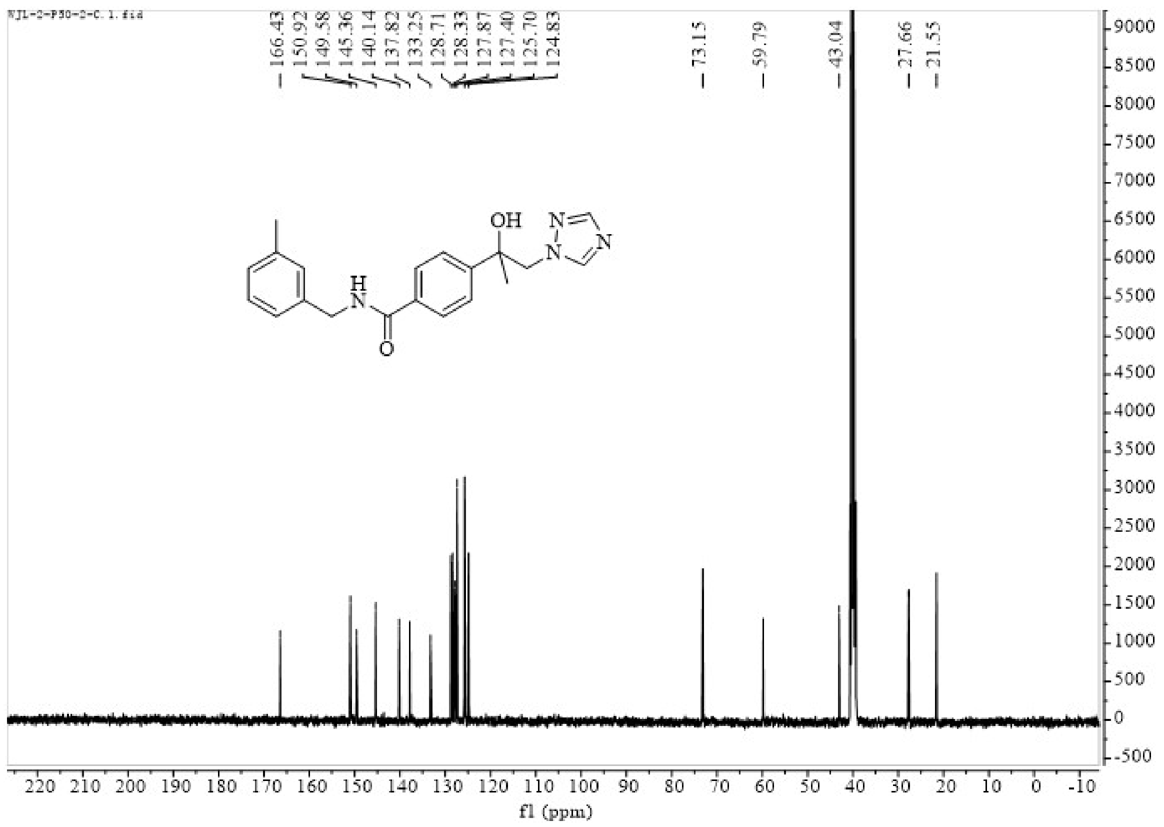

Figure S40. ^{13}C NMR spectrum of **6c**

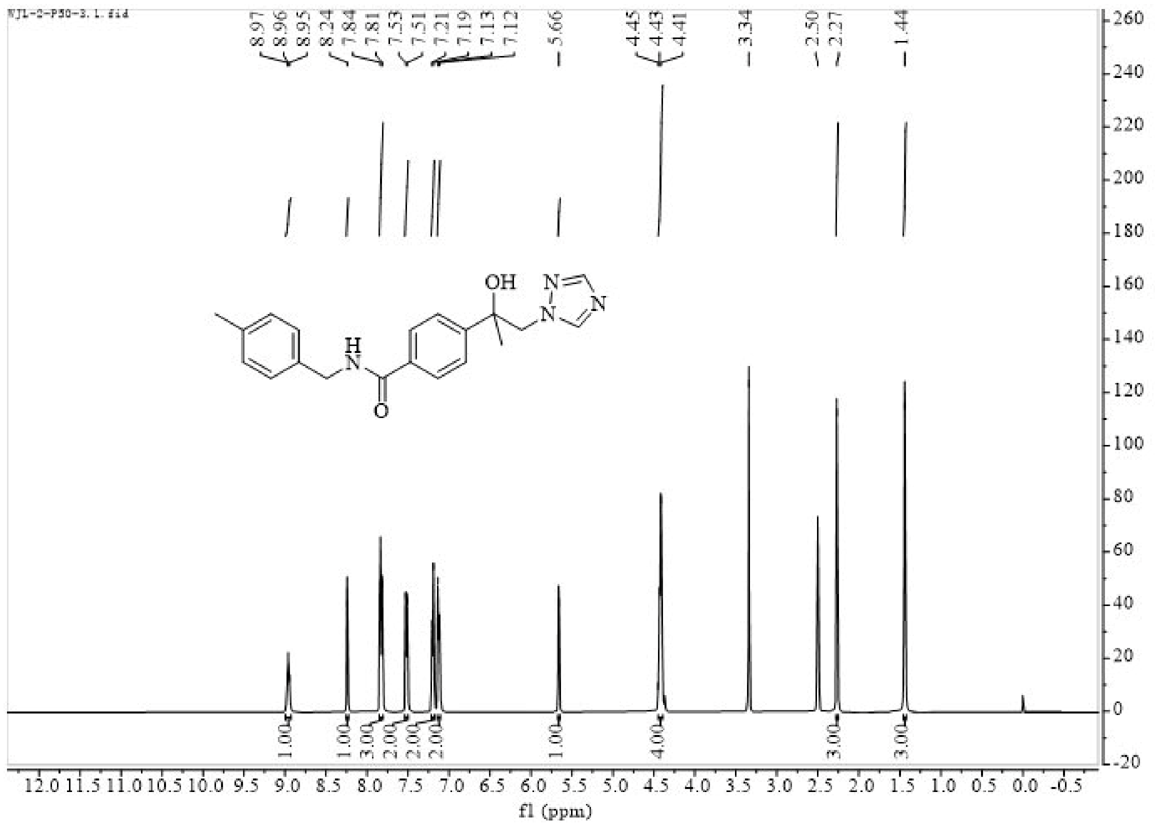

Figure S41. ^{1}H NMR spectrum of **6d**

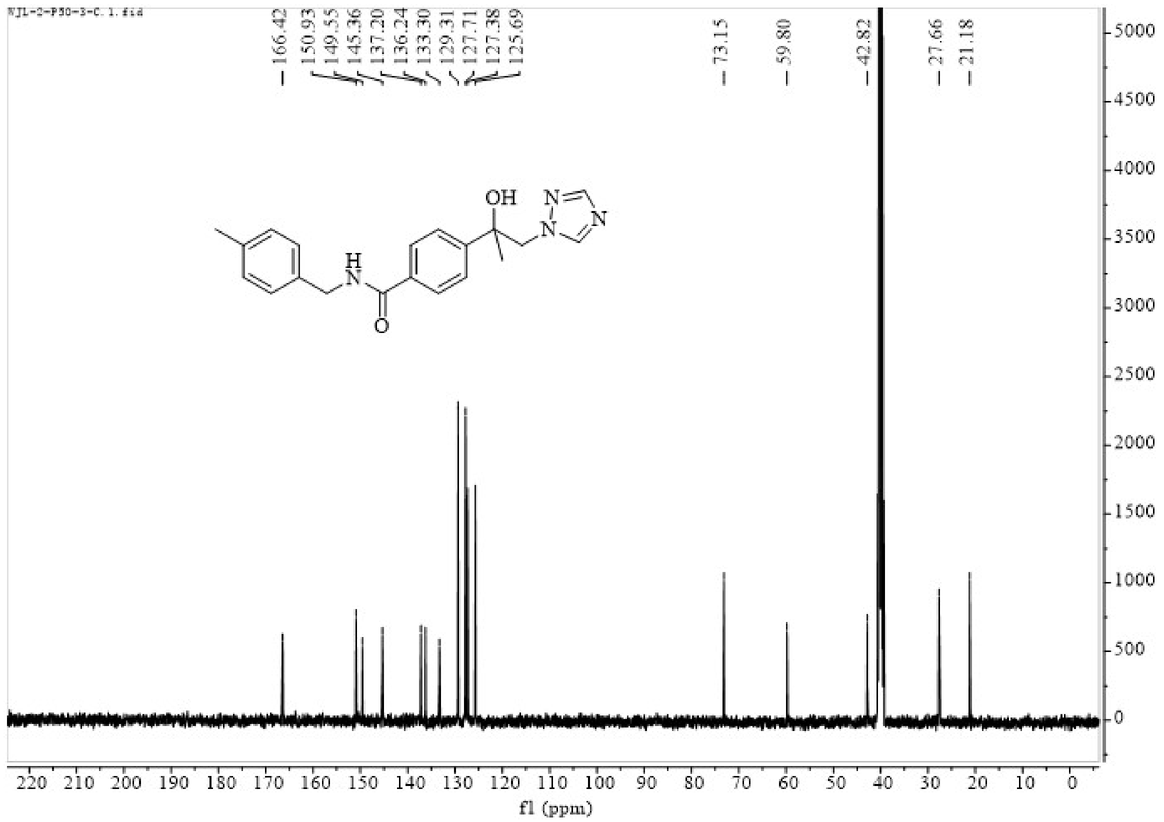

Figure S42. ^{13}C NMR spectrum of **4d**

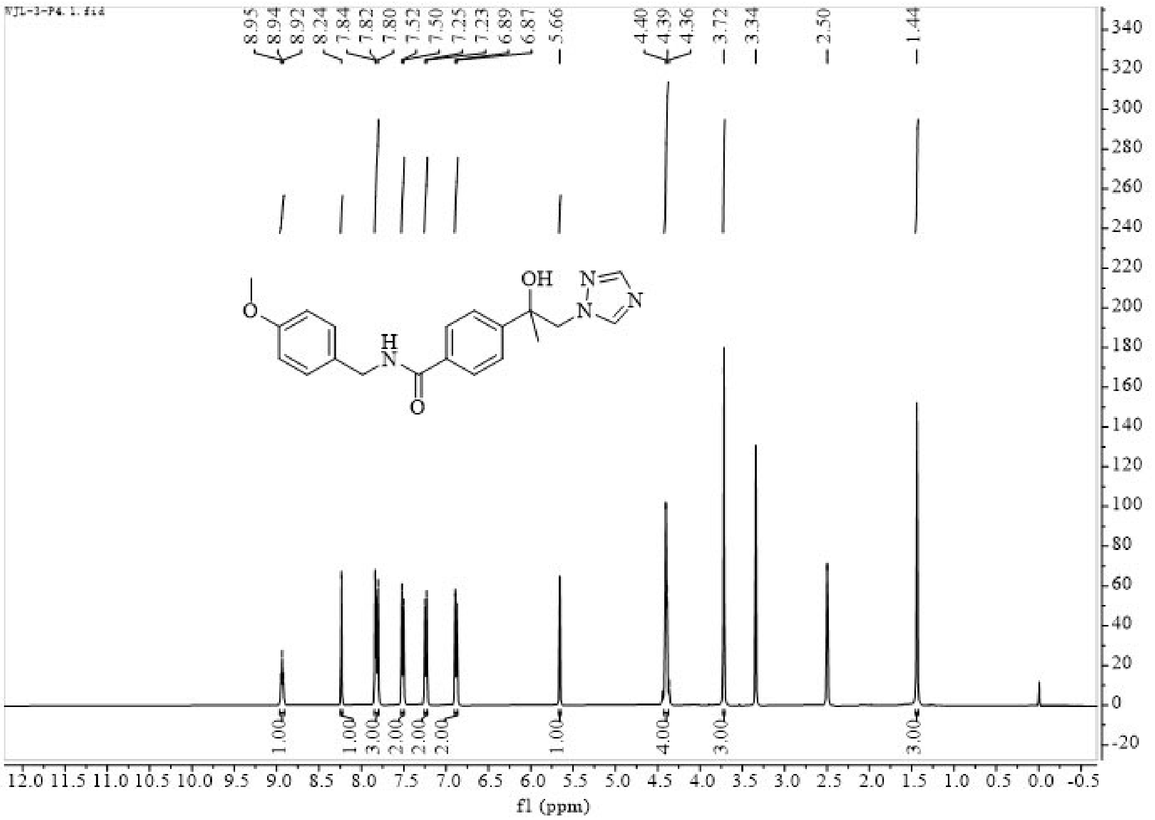

Figure S43. ^{1}H NMR spectrum of **6e**

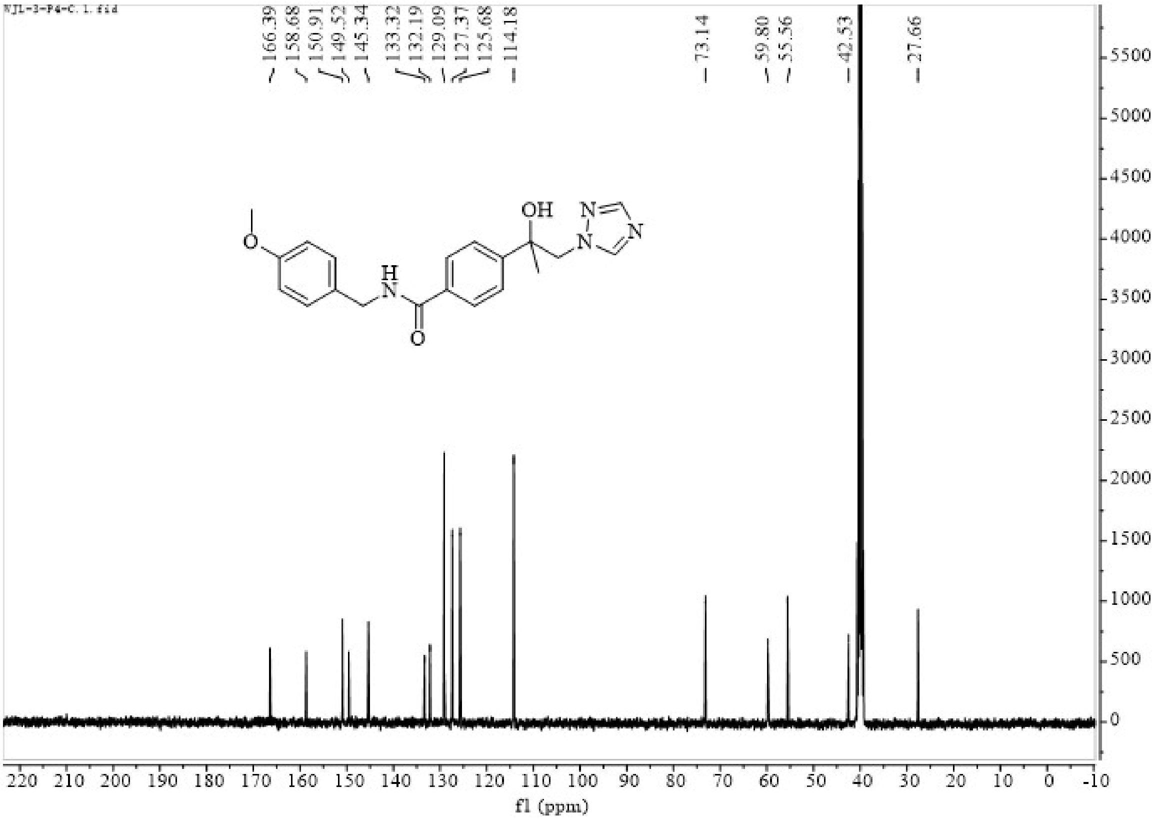

Figure S44. ^{13}C NMR spectrum of **6e**

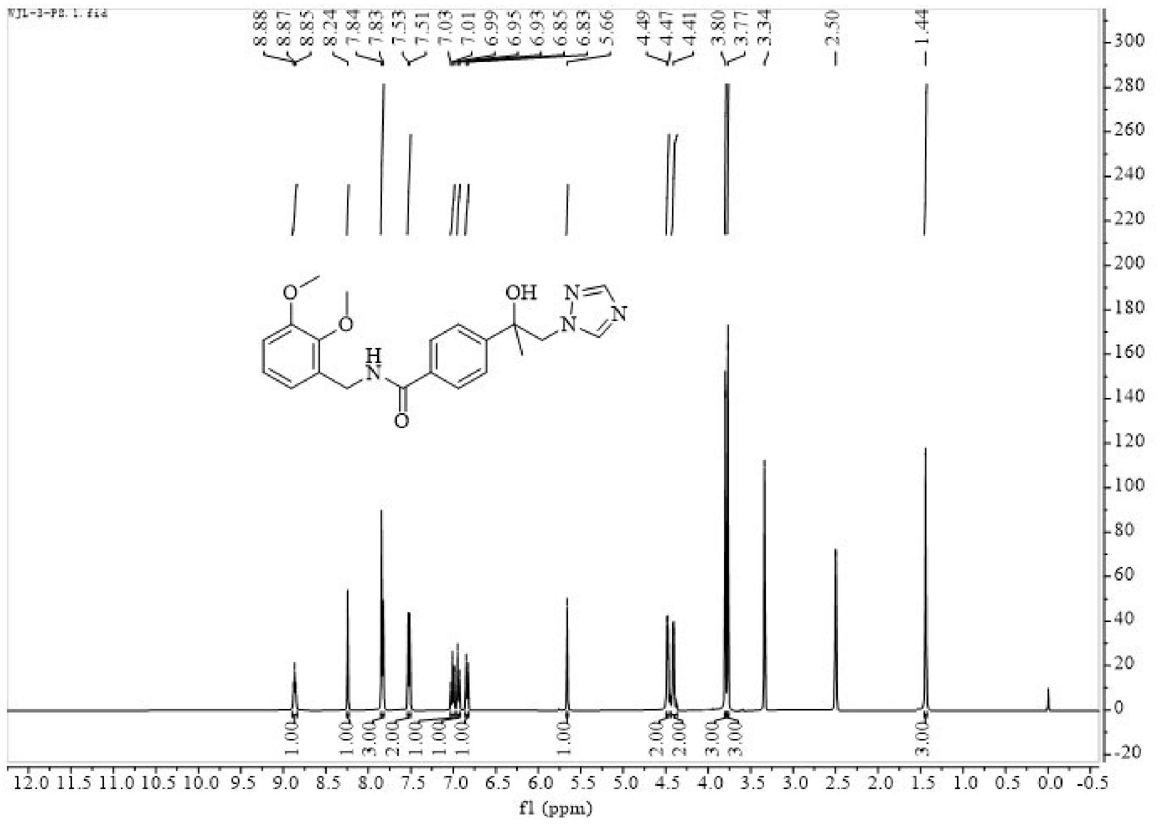

Figure S45. ^{1}H NMR spectrum of **6f**

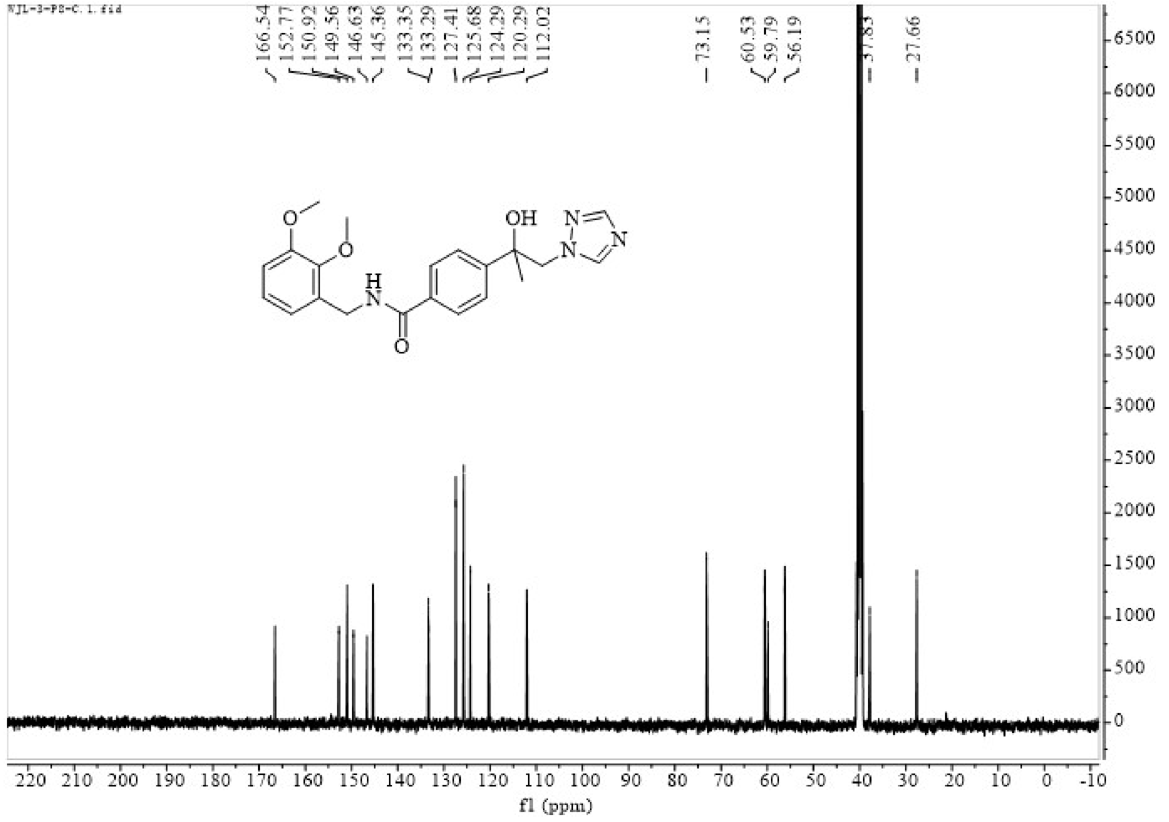

Figure S46. ^{13}C NMR spectrum of **6f**

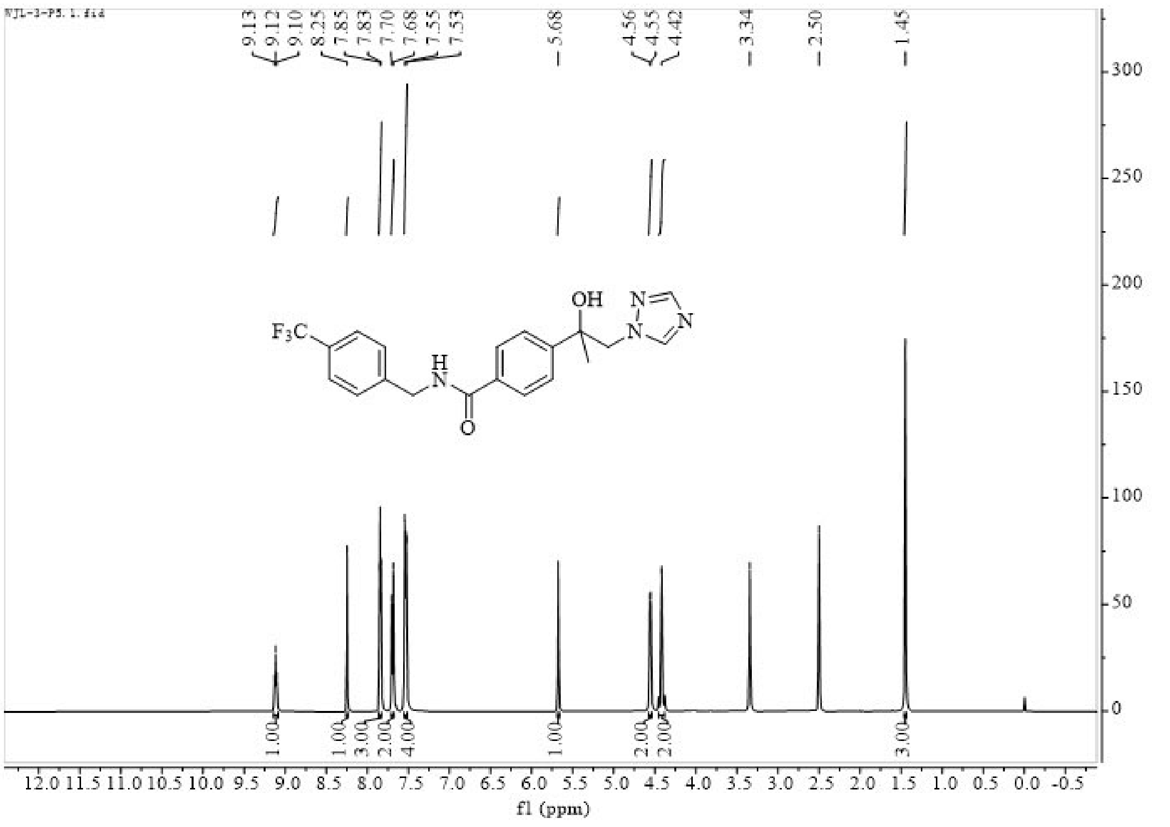

Figure S47. ^{1}H NMR spectrum of **6g**

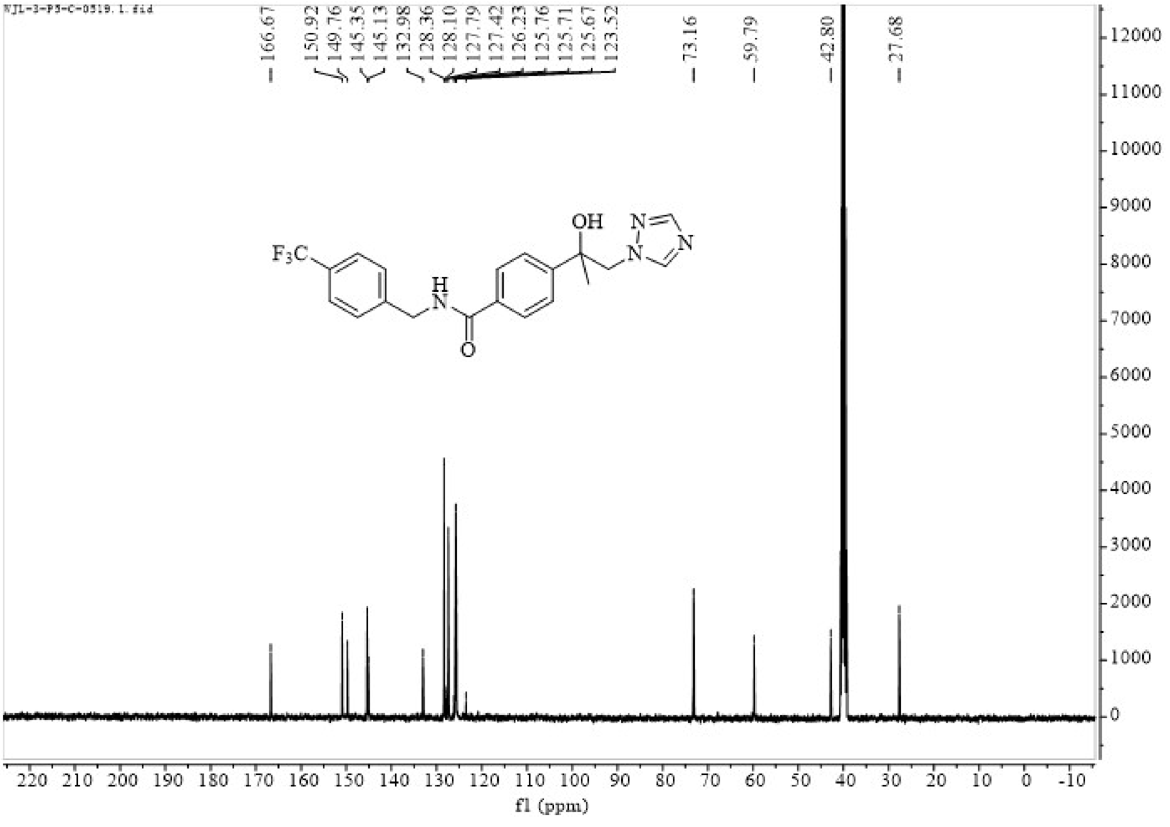

Figure S48. ^{13}C NMR spectrum of **6g**

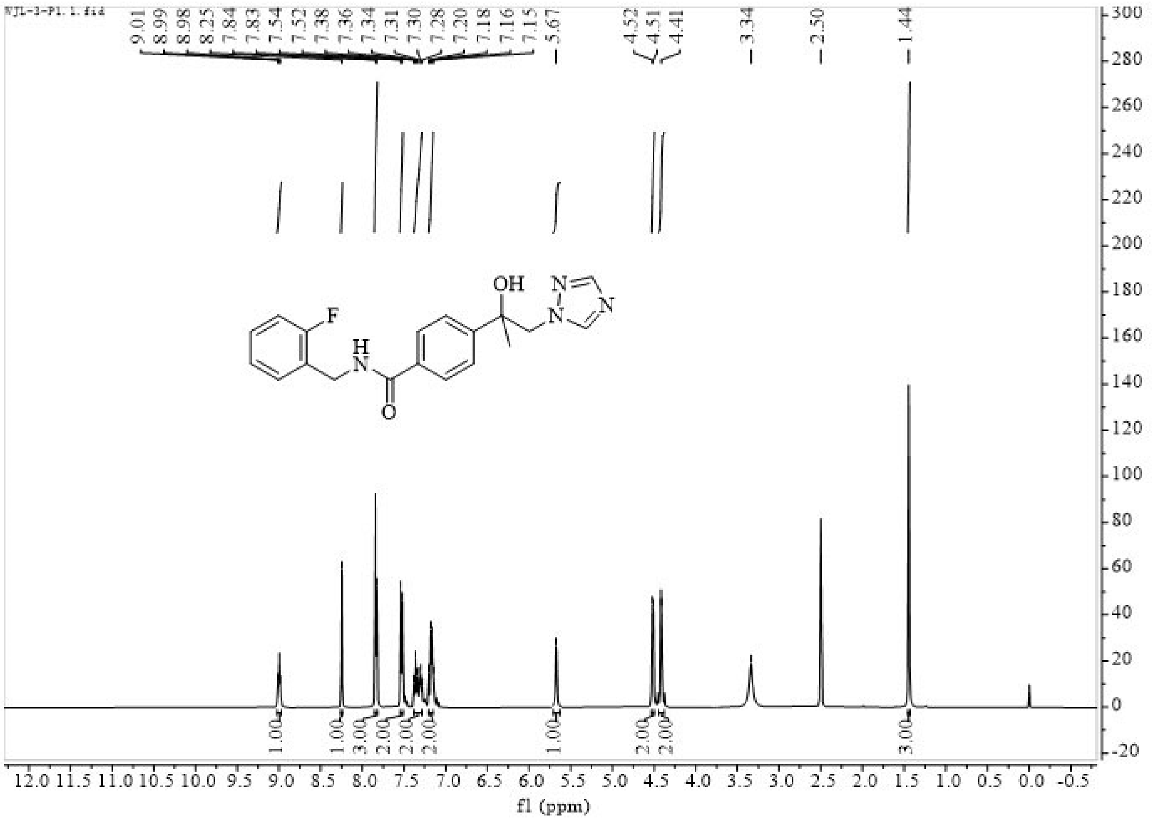

Figure S49. ^{1}H NMR spectrum of **6h**

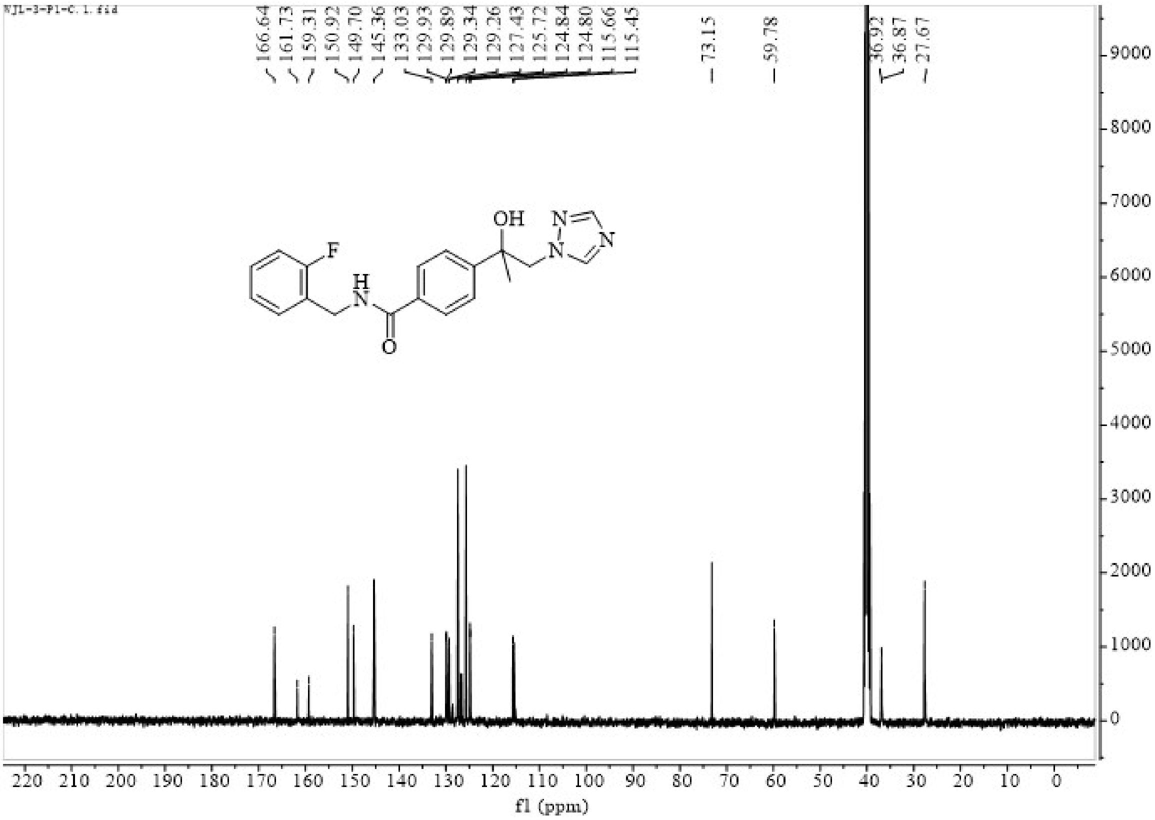

Figure S50. ^{13}C NMR spectrum of **6h**

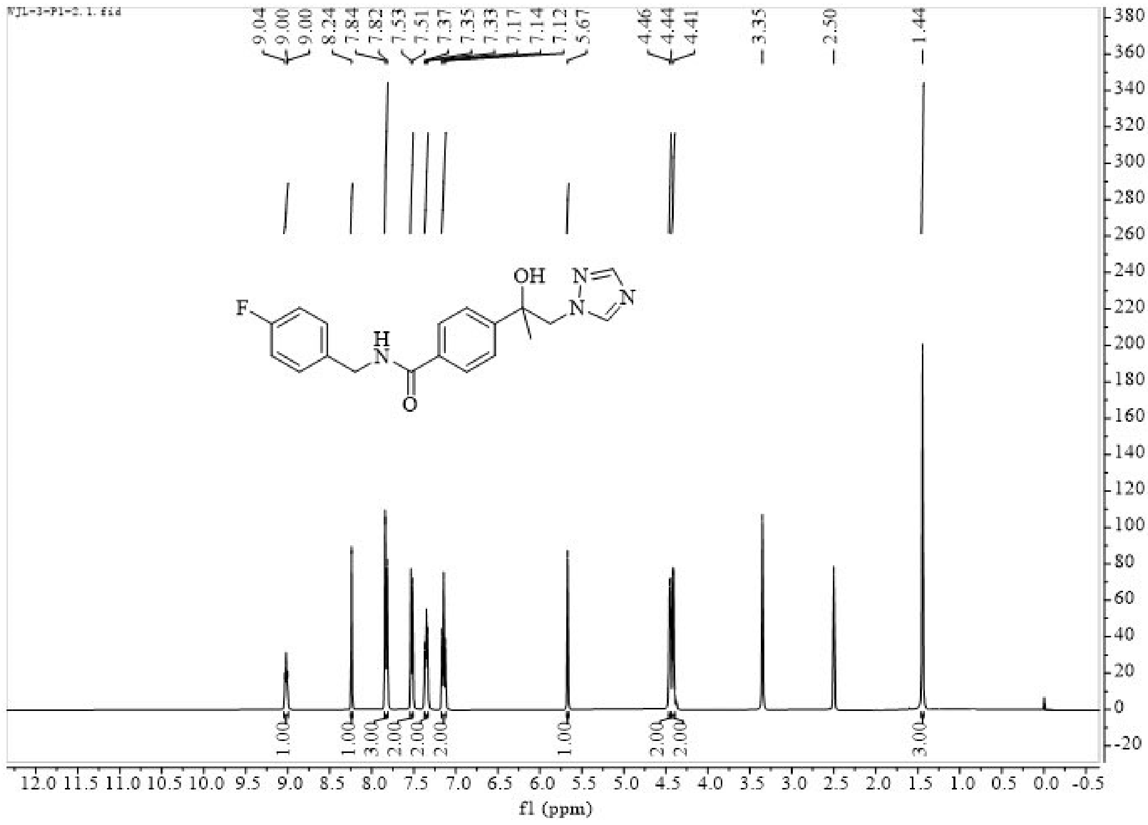

Figure S51. ^{1}H NMR spectrum of **6i**

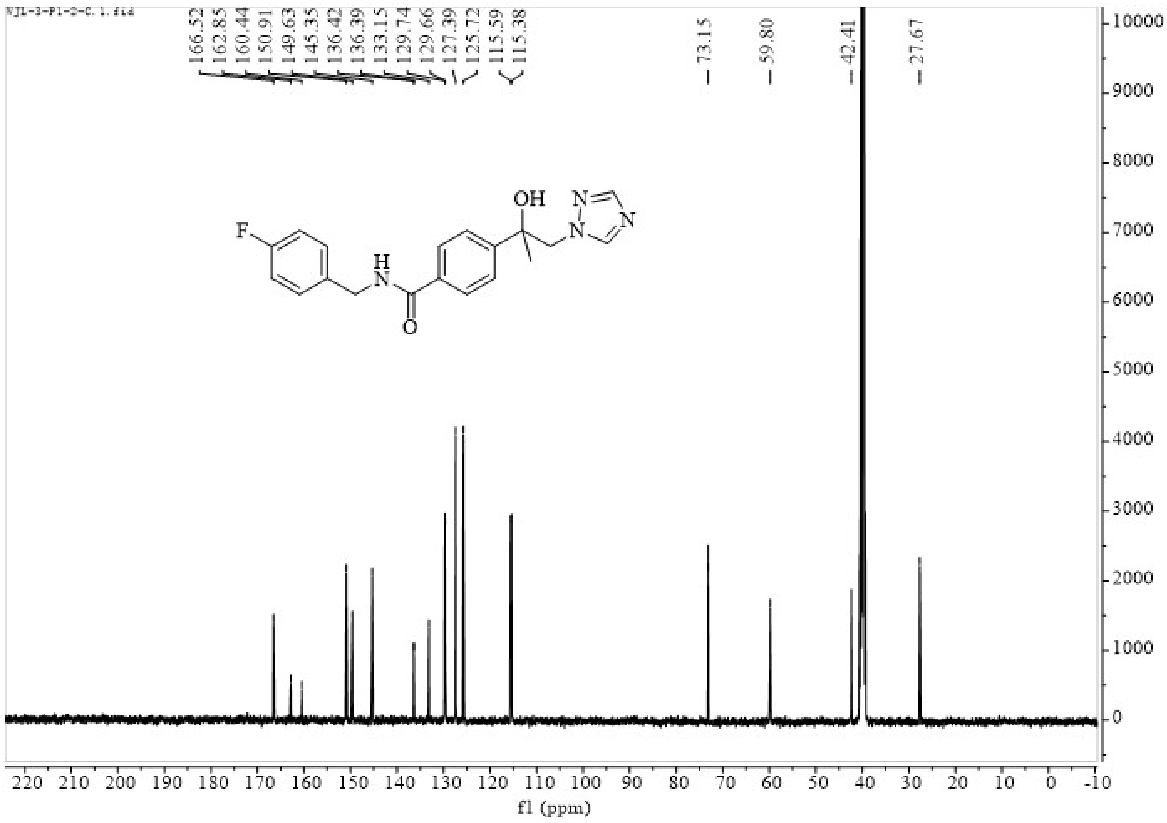

Figure S52. ^{13}C NMR spectrum of **6i**

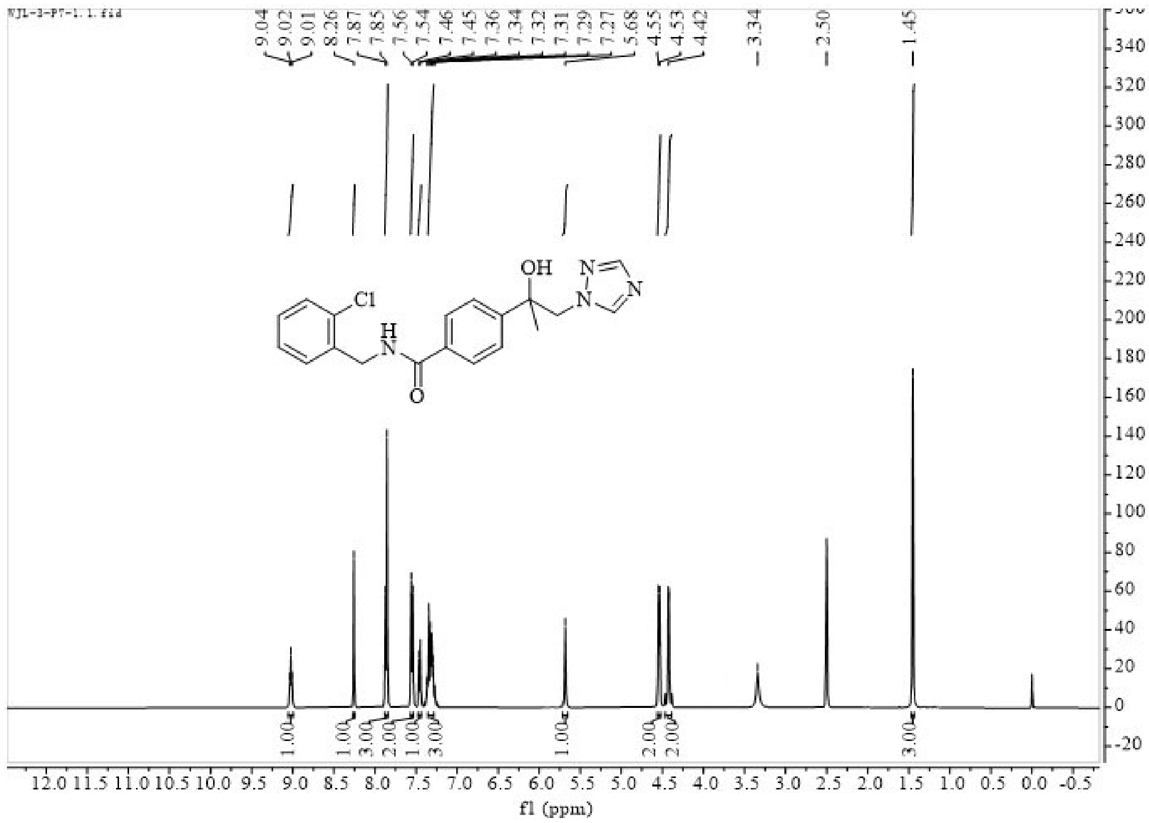

Figure S53. ^{1}H NMR spectrum of **6j**

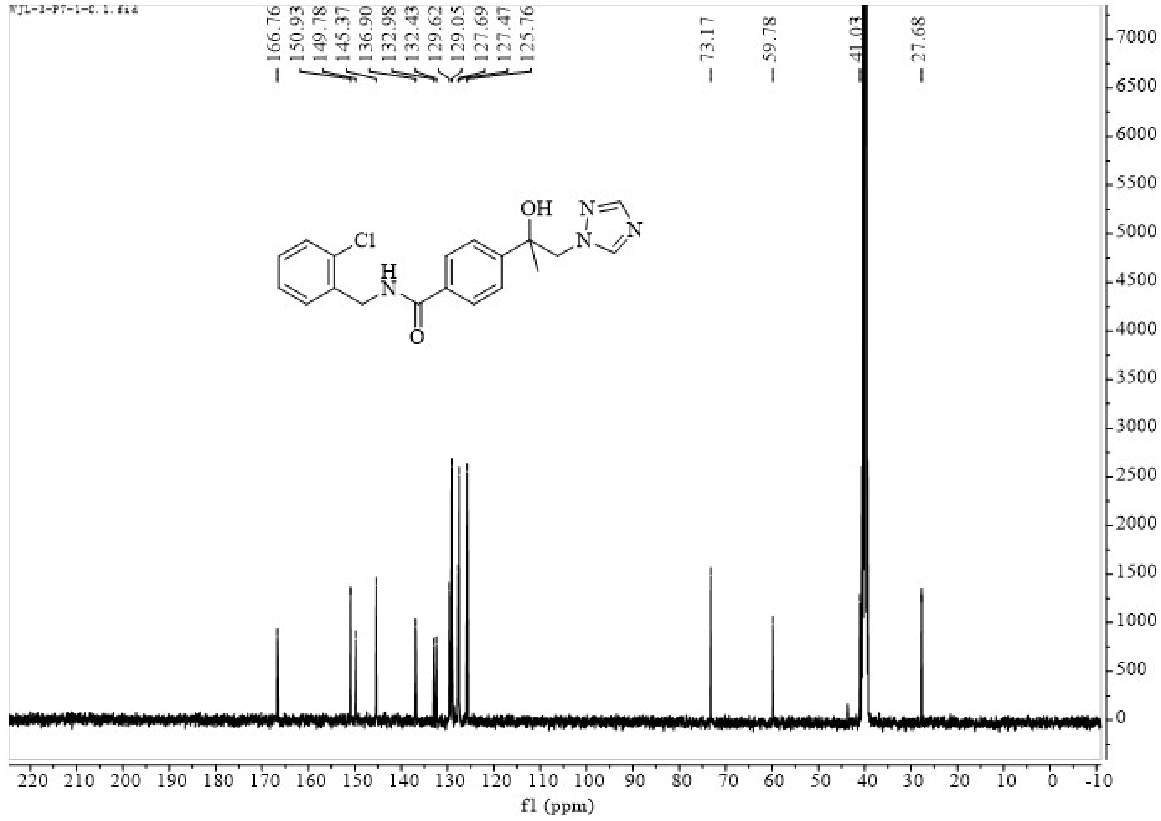

Figure S54. ^{13}C NMR spectrum of **6j**

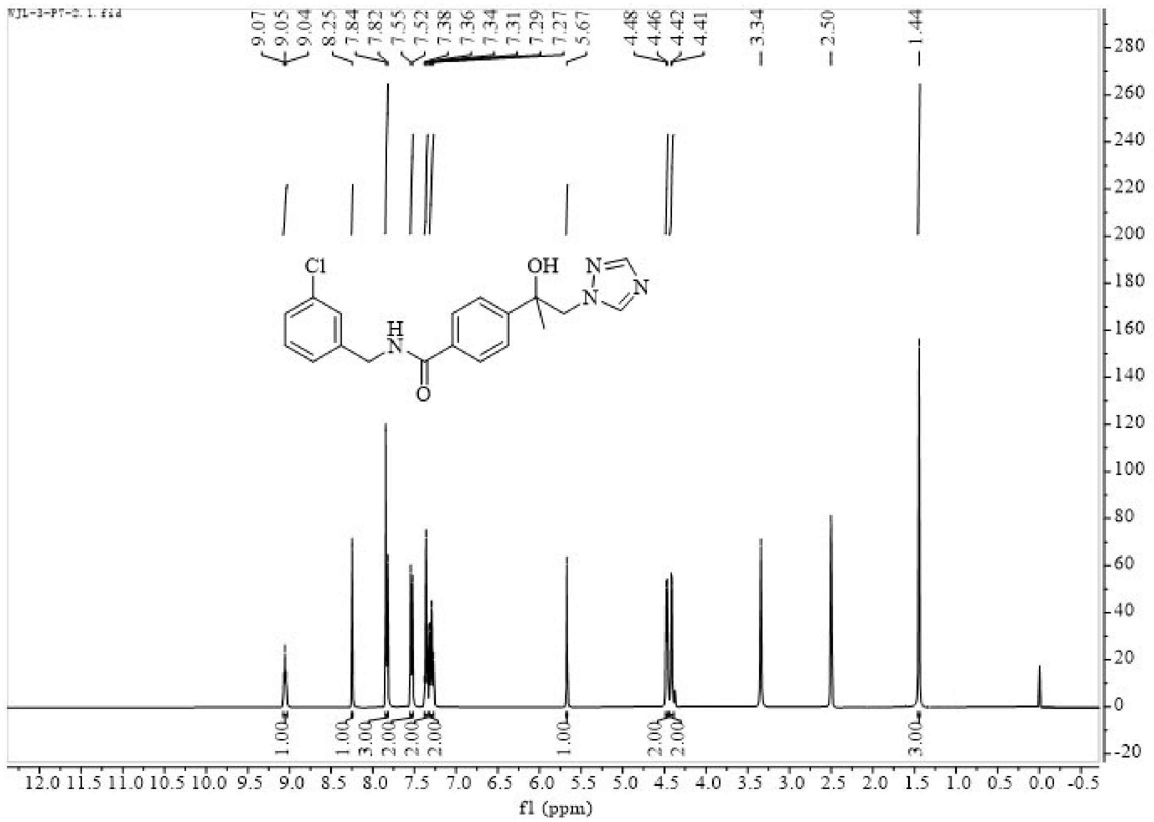

Figure S55. ^{1}H NMR spectrum of **6k**

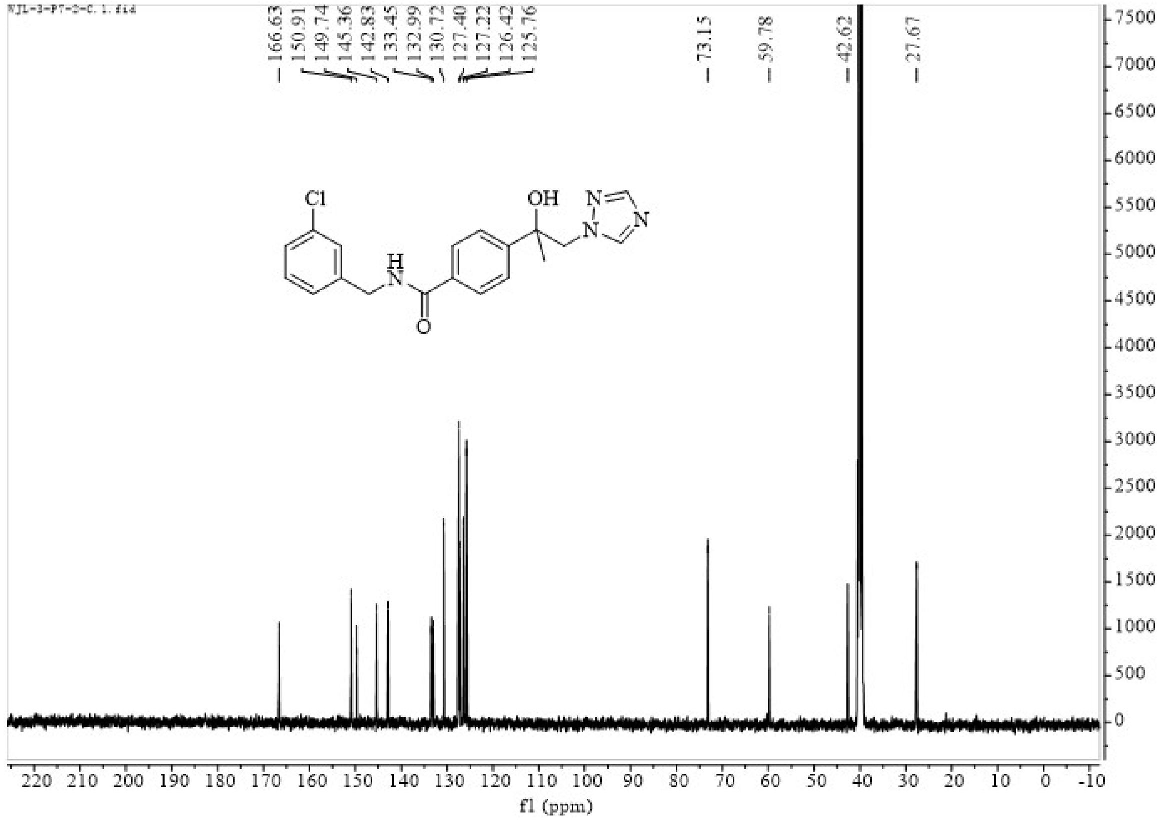

Figure S56. ^{13}C NMR spectrum of **6k**

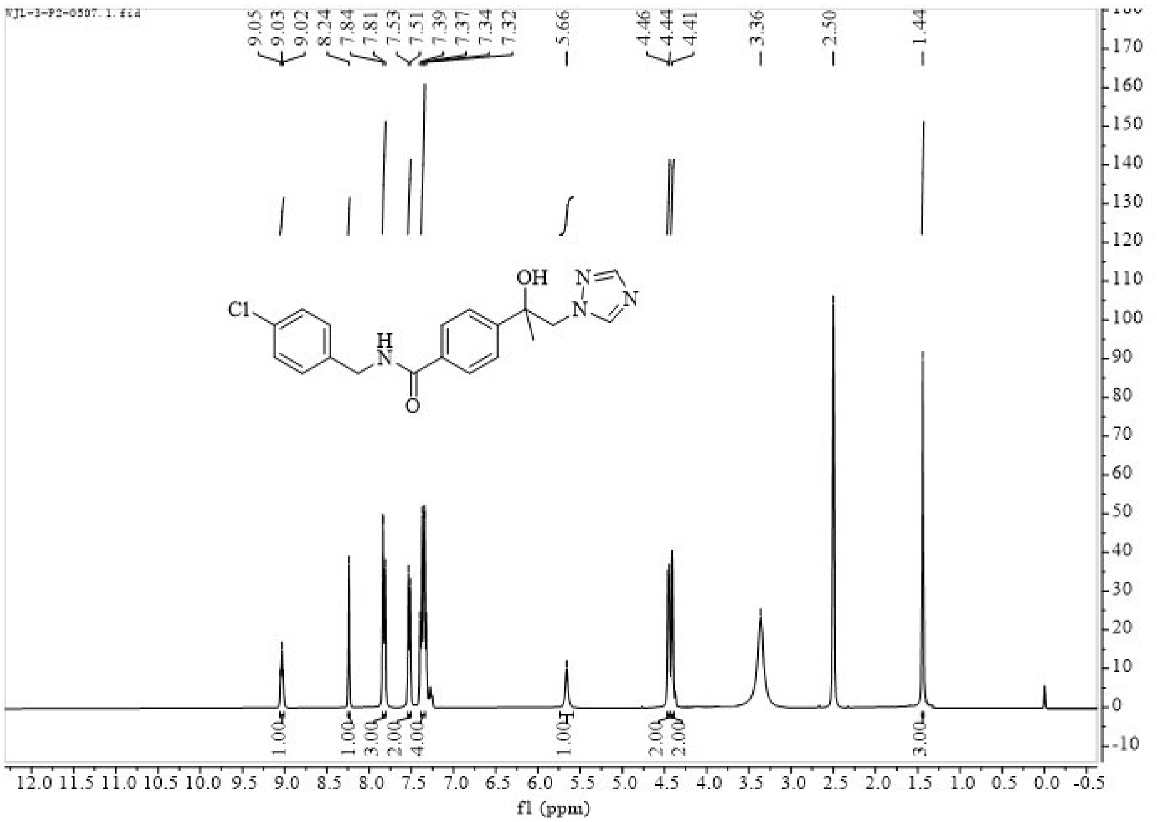

Figure S57. ^{1}H NMR spectrum of **6l**

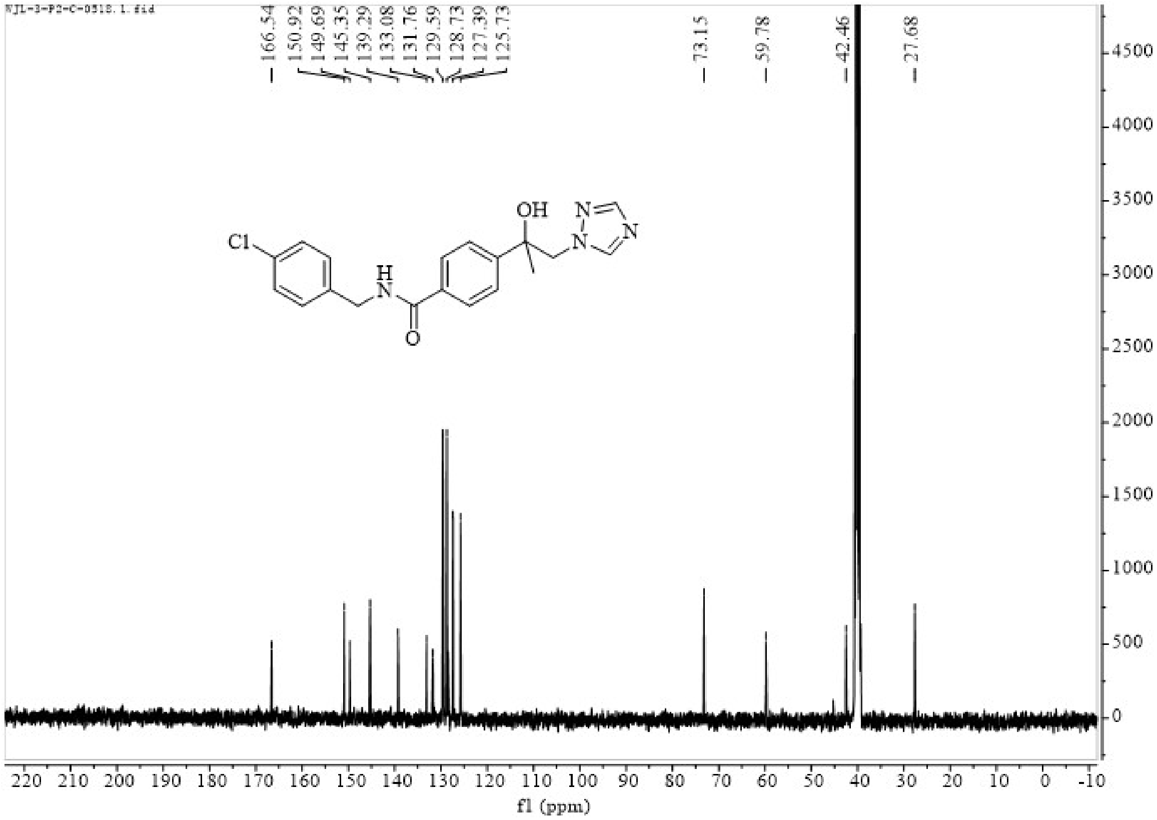

Figure S58. ^{13}C NMR spectrum of **6l**

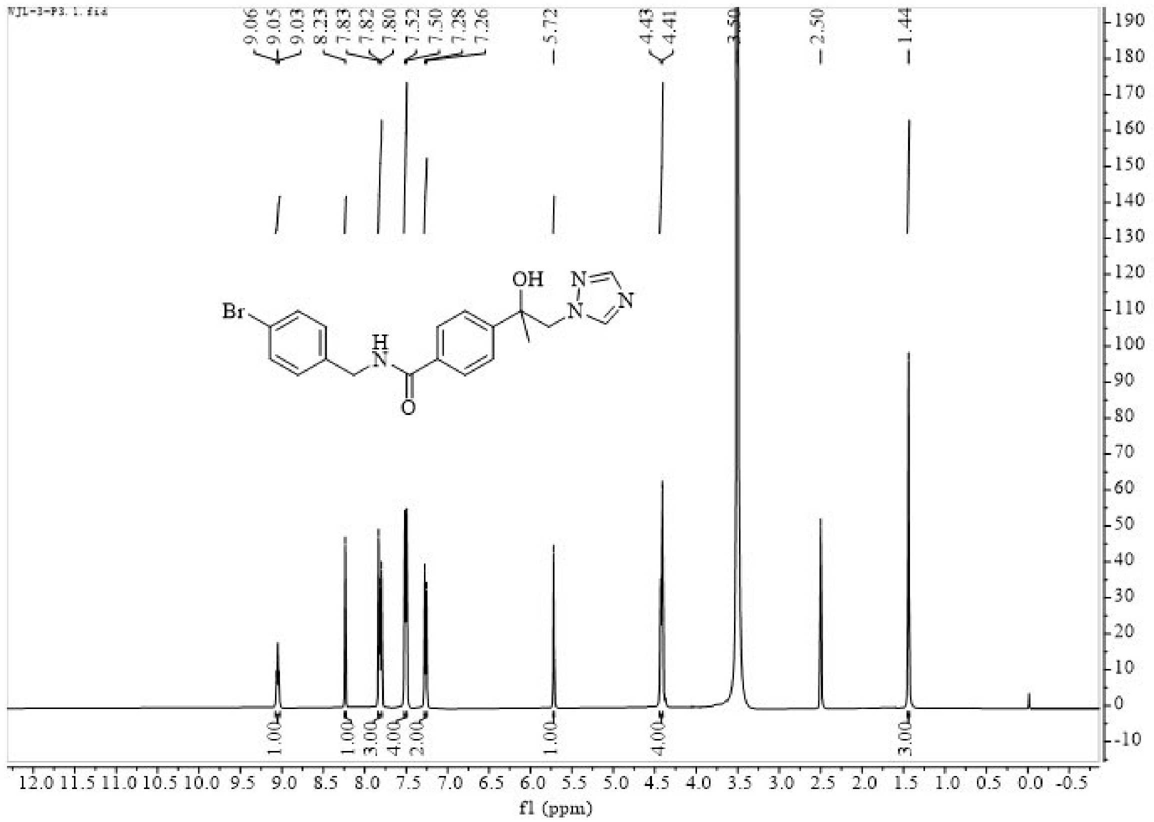

Figure S59. ^{1}H NMR spectrum of **6m**

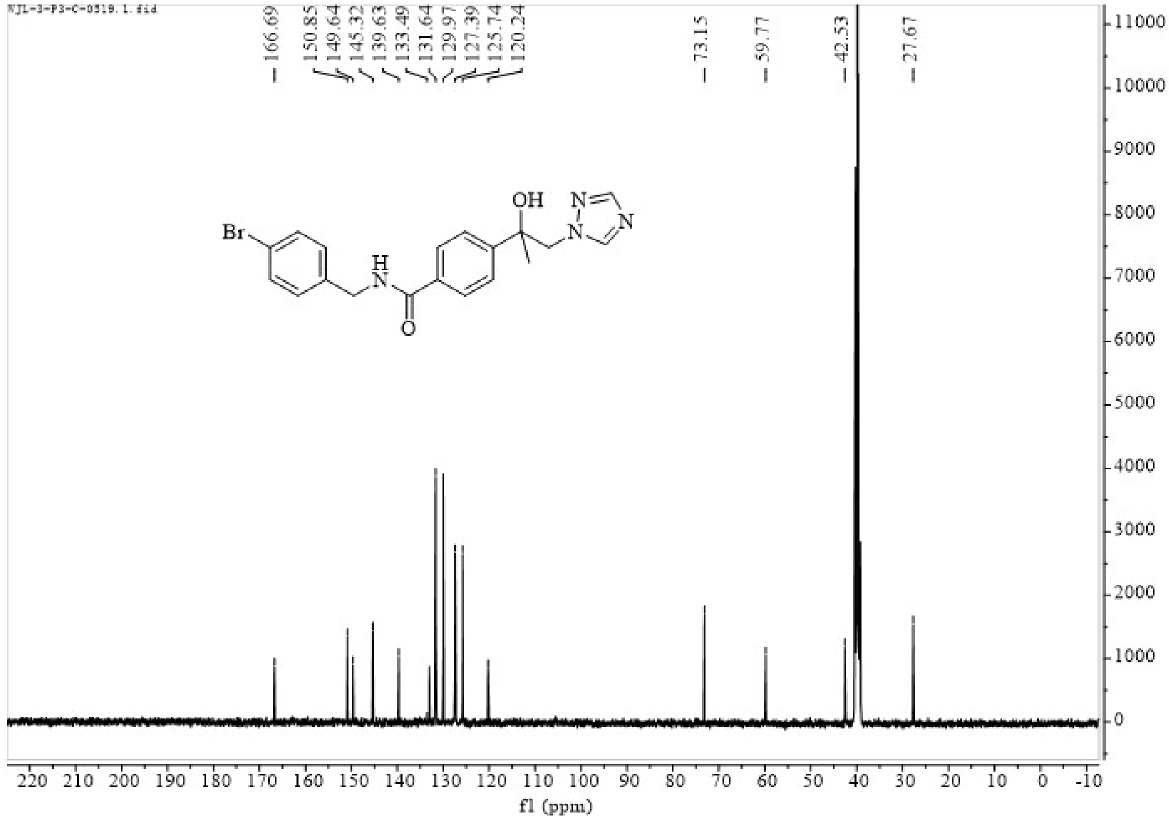

Figure S60. ^{13}C NMR spectrum of **6m**

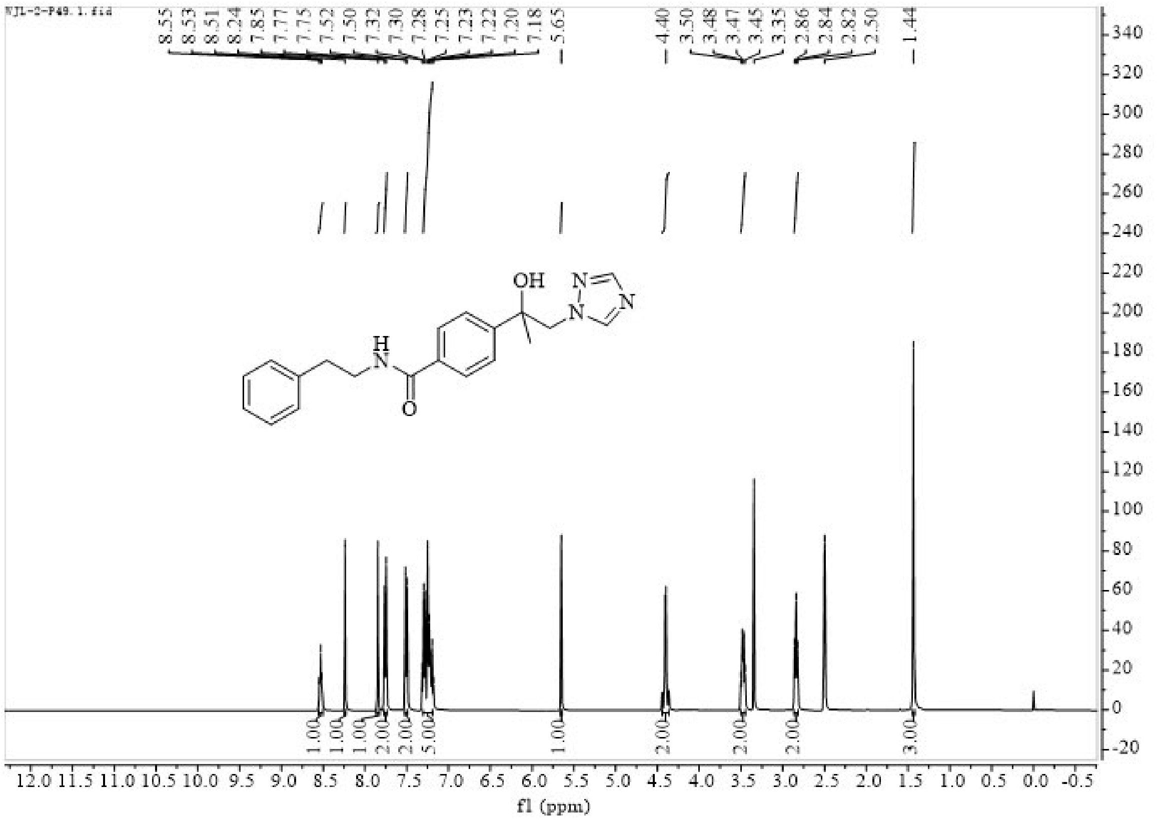

Figure S61. ^{1}H NMR spectrum of **7a**

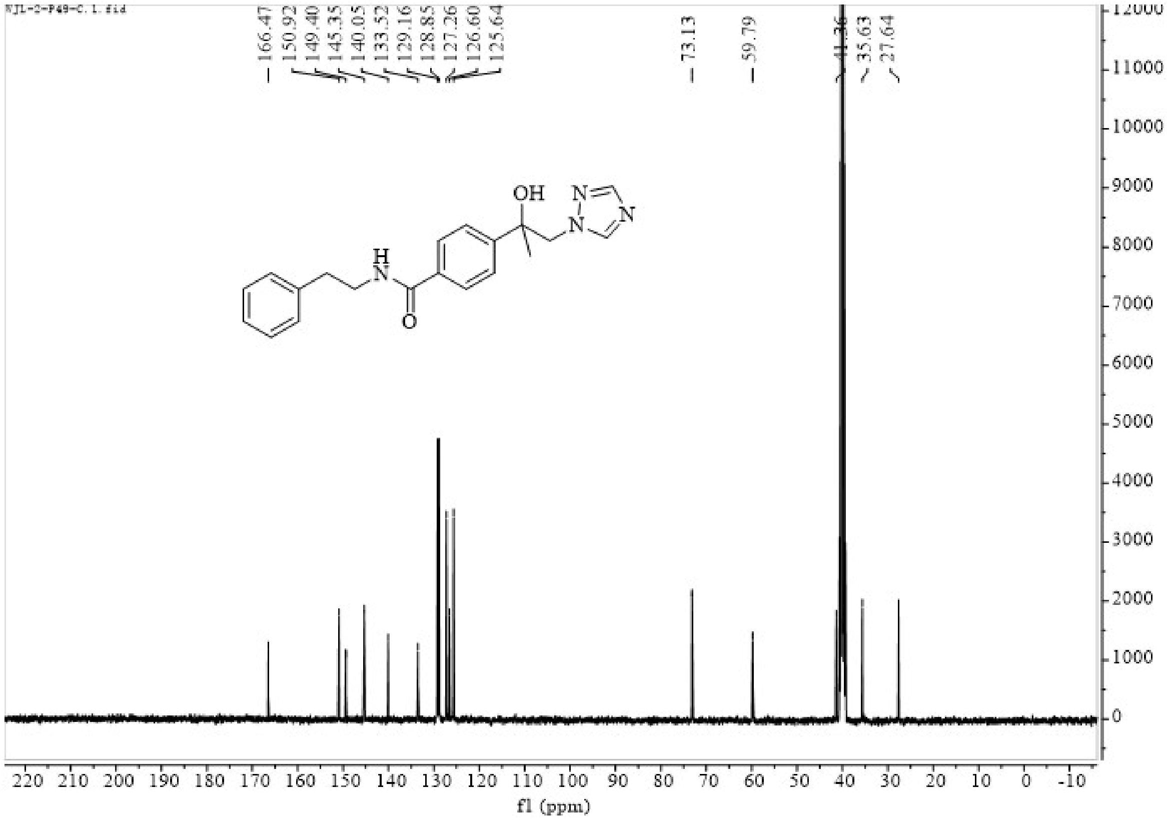

Figure S62. ^{13}C NMR spectrum of **7a**

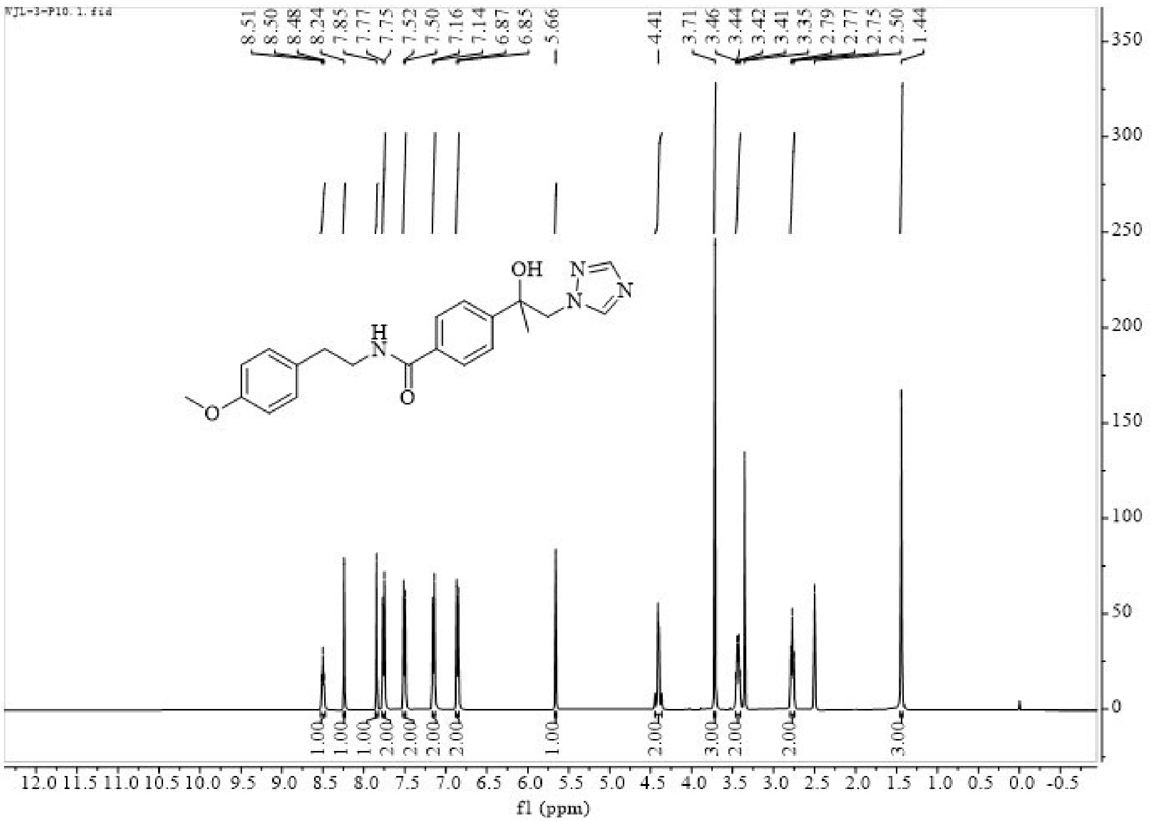

Figure S63. ^{1}H NMR spectrum of **7b**

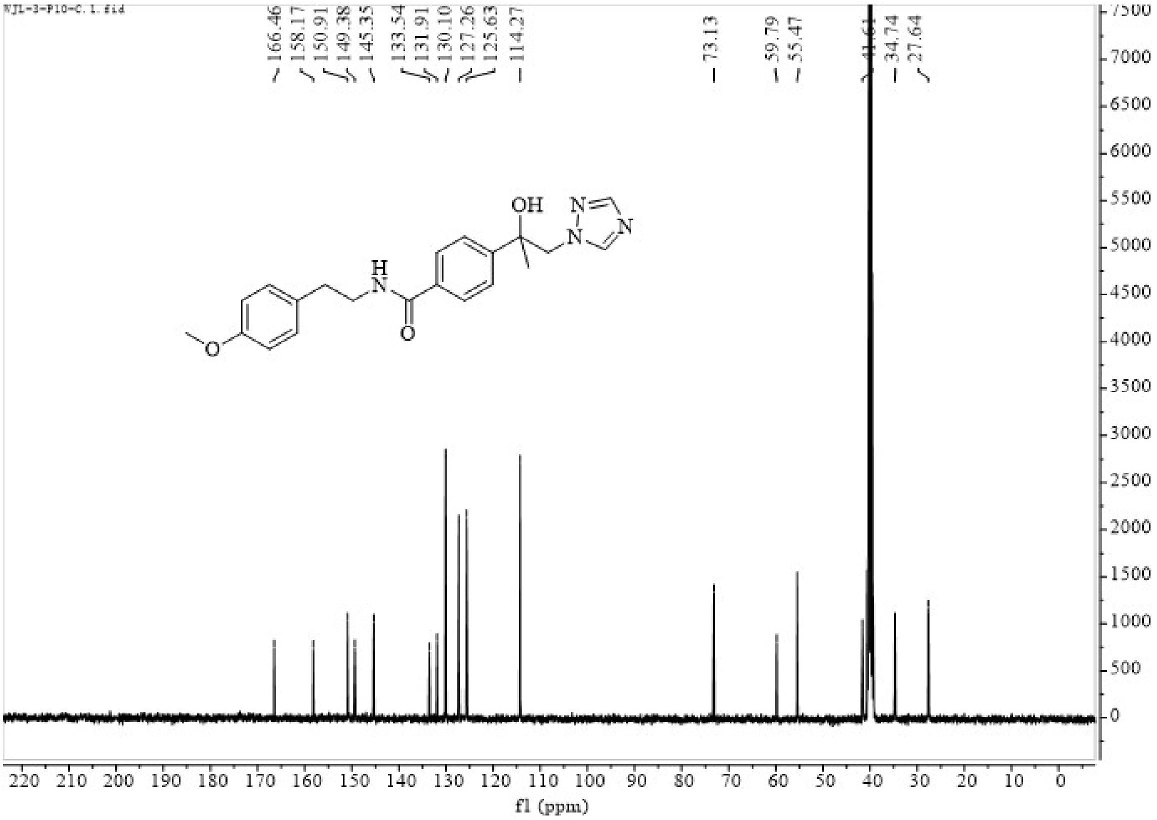

Figure S64. ^{13}C NMR spectrum of **7b**

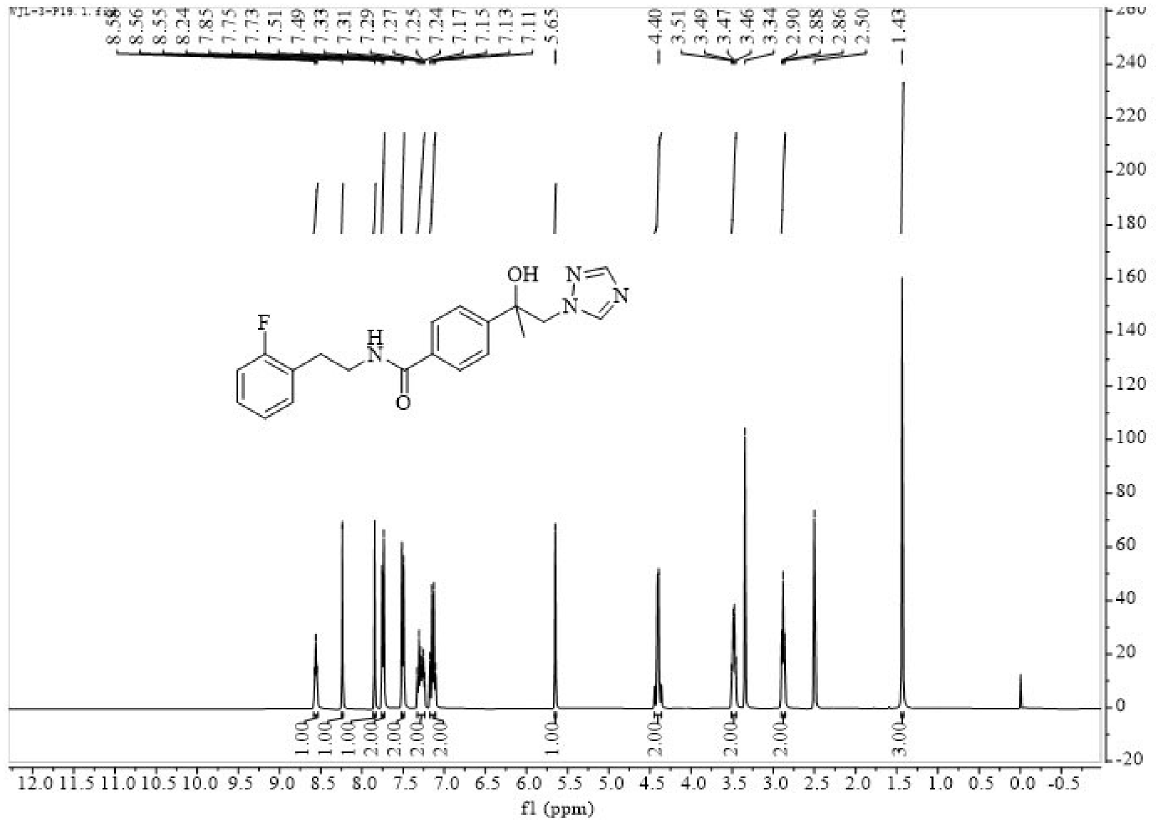

Figure S65. ^{1}H NMR spectrum of **7c**

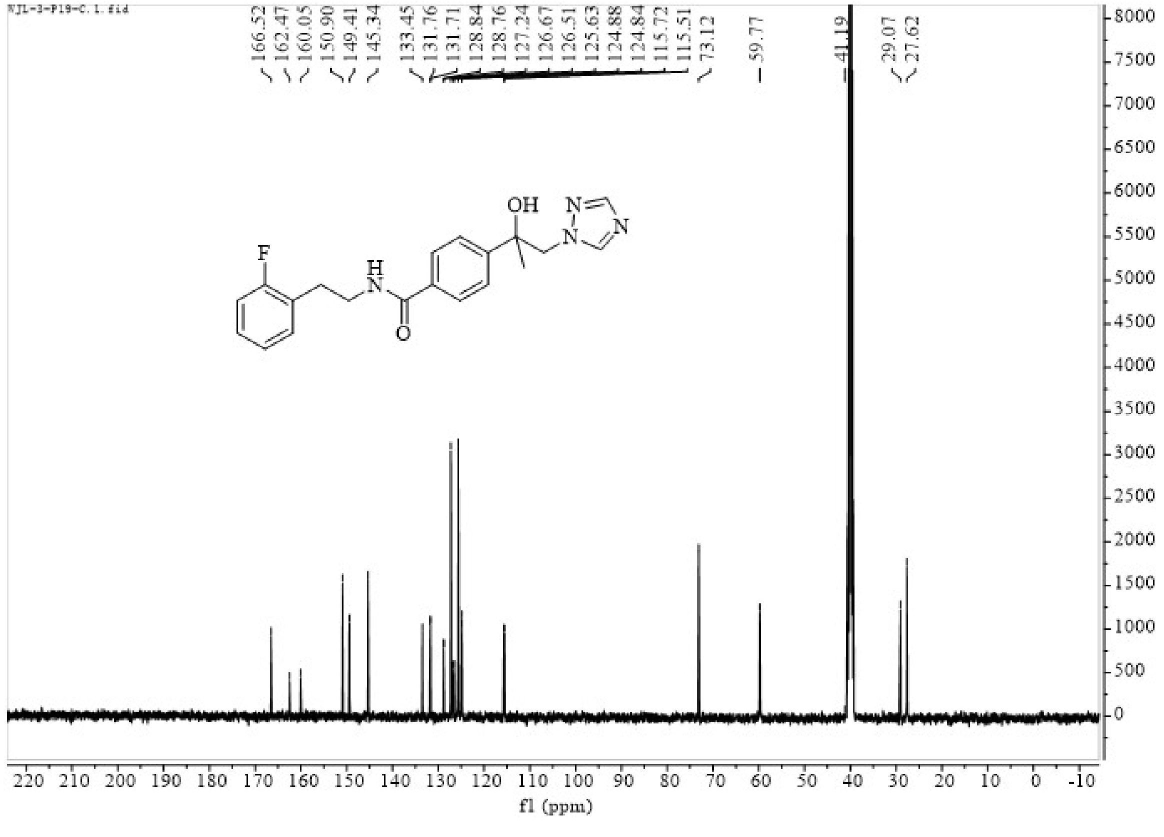

Figure S66. ^{13}C NMR spectrum of **7c**

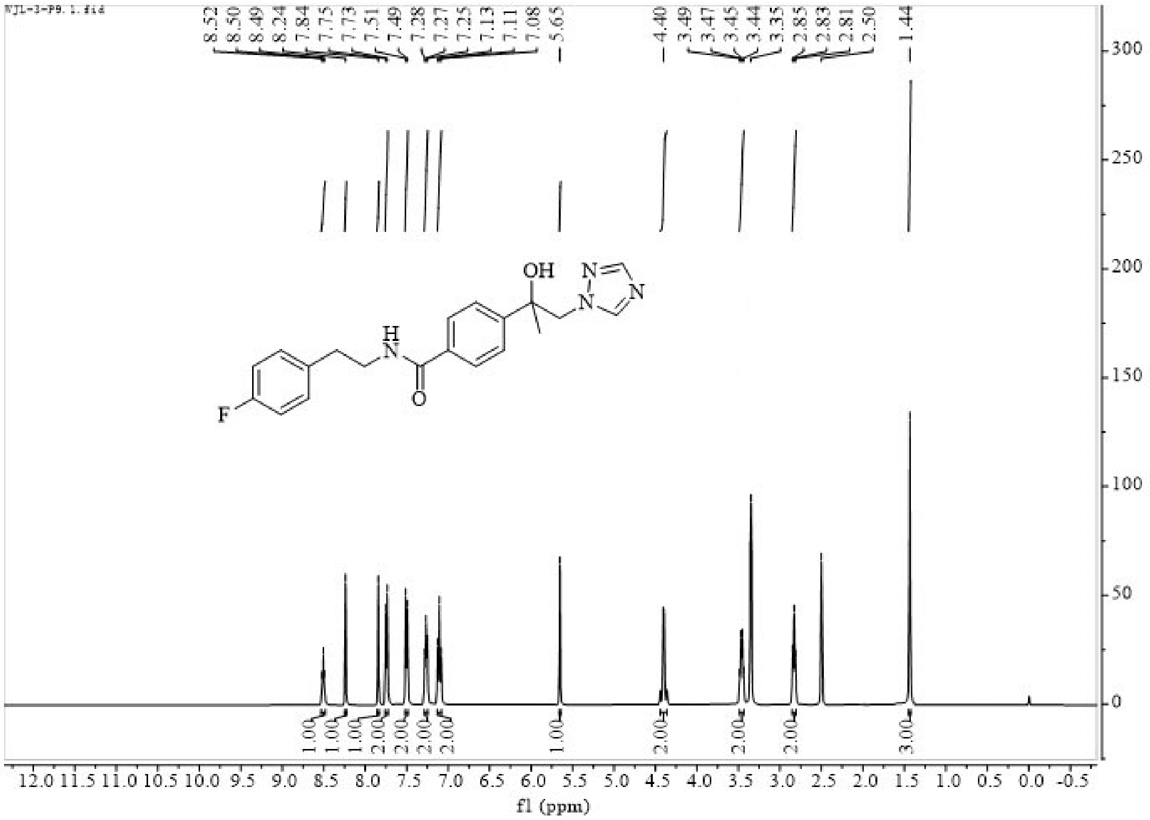

Figure S67. ^{1}H NMR spectrum of **7d**

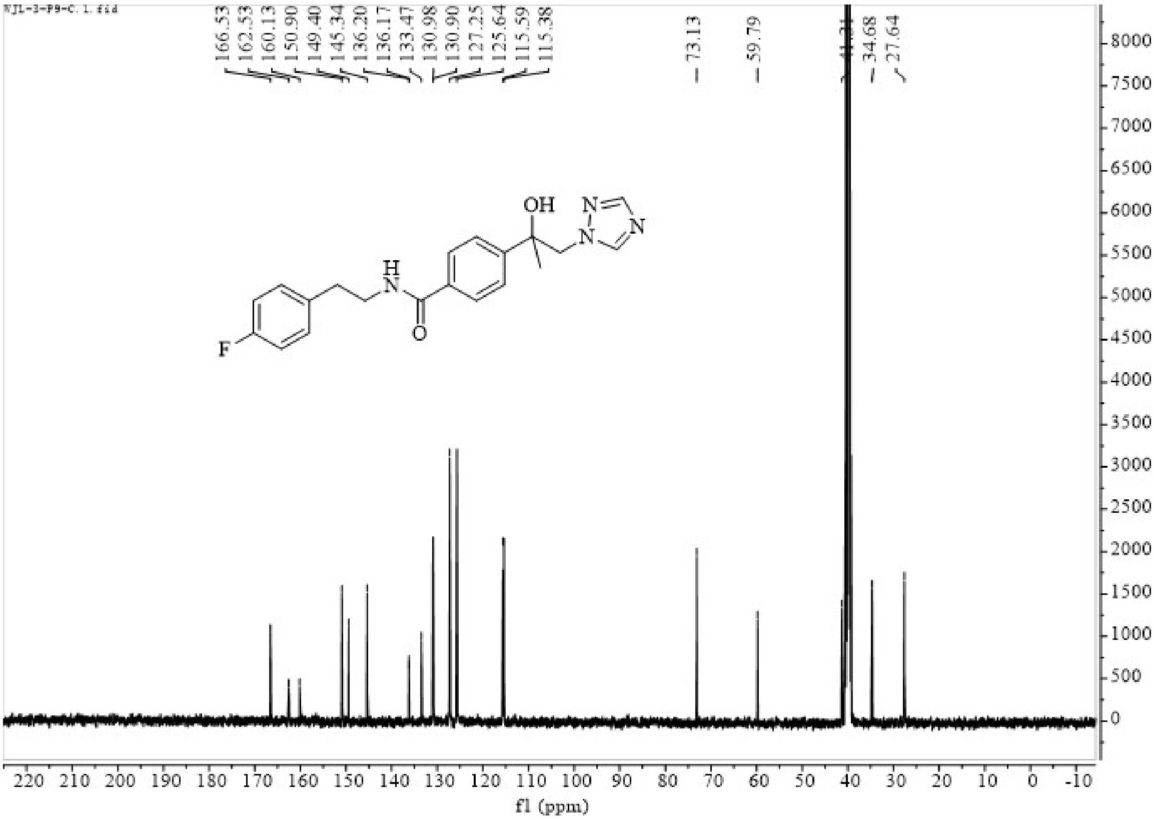

Figure S68. ^{13}C NMR spectrum of **7d**

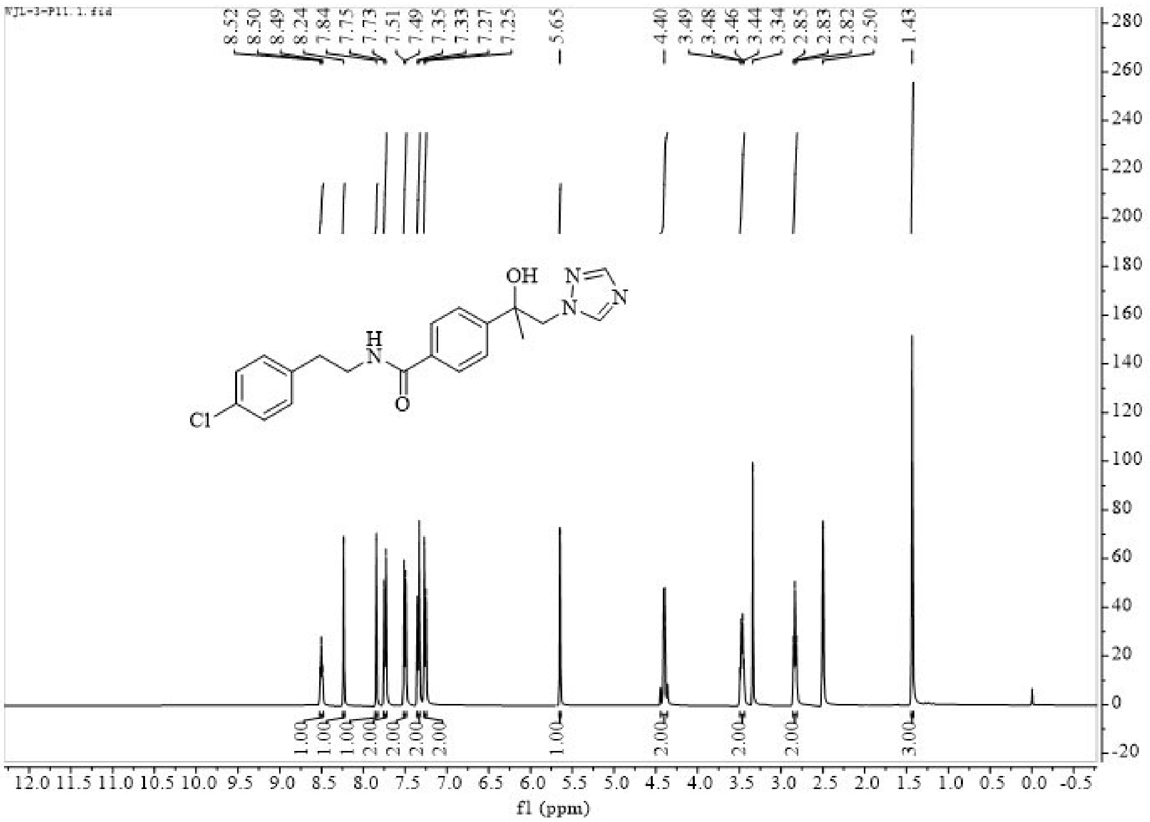

Figure S69. ^{1}H NMR spectrum of **7e**

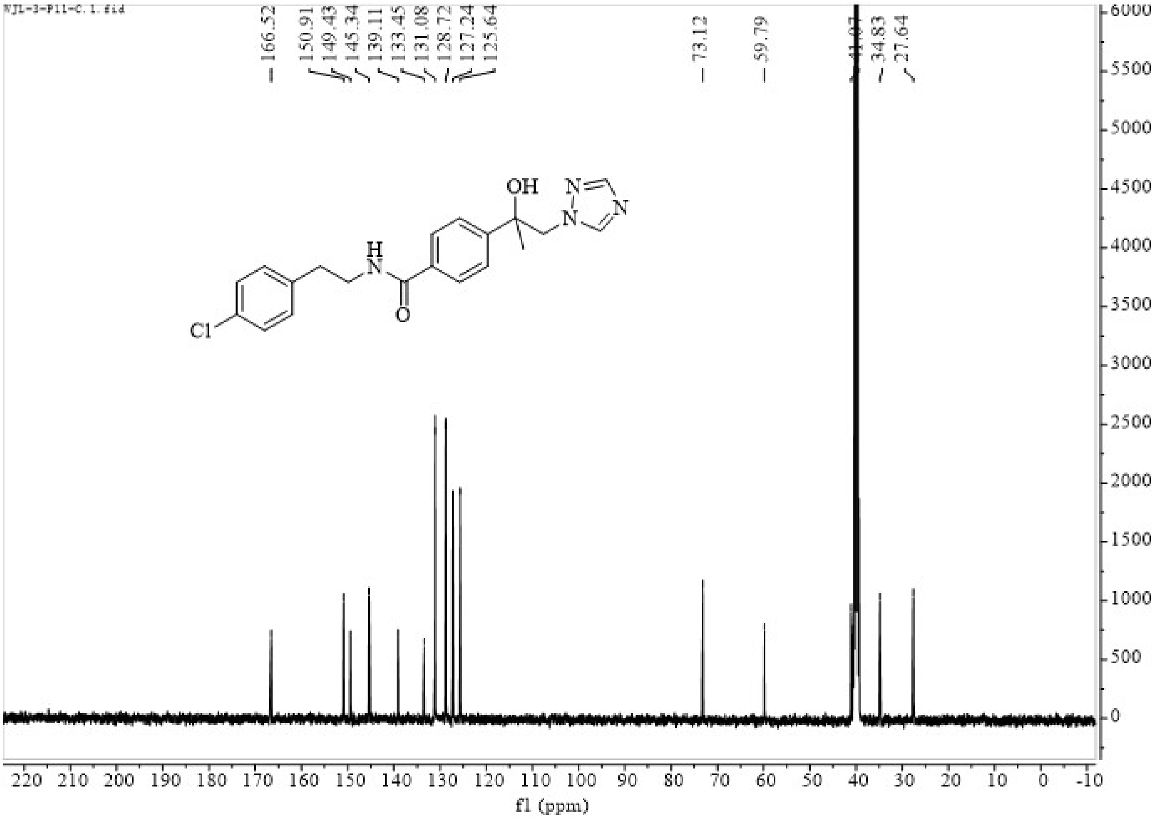

Figure S70. ^{13}C NMR spectrum of **7e**

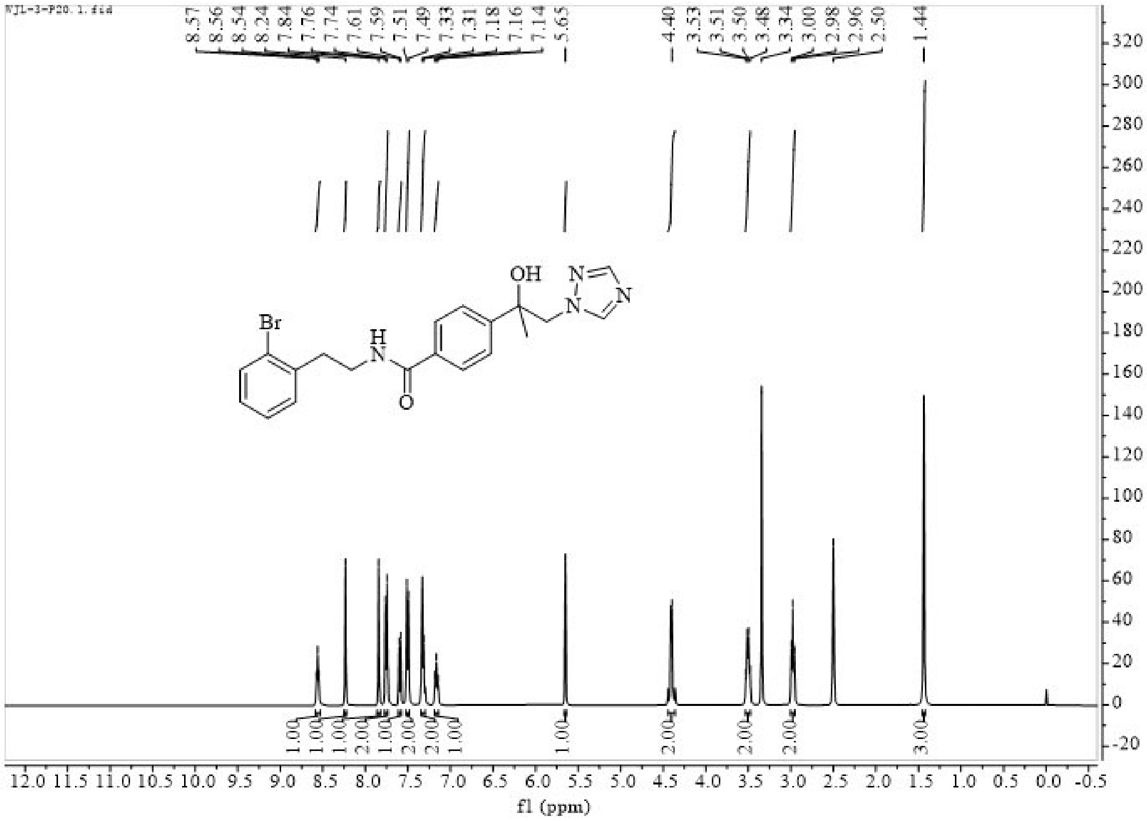

Figure S71. ^{1}H NMR spectrum of **7f**

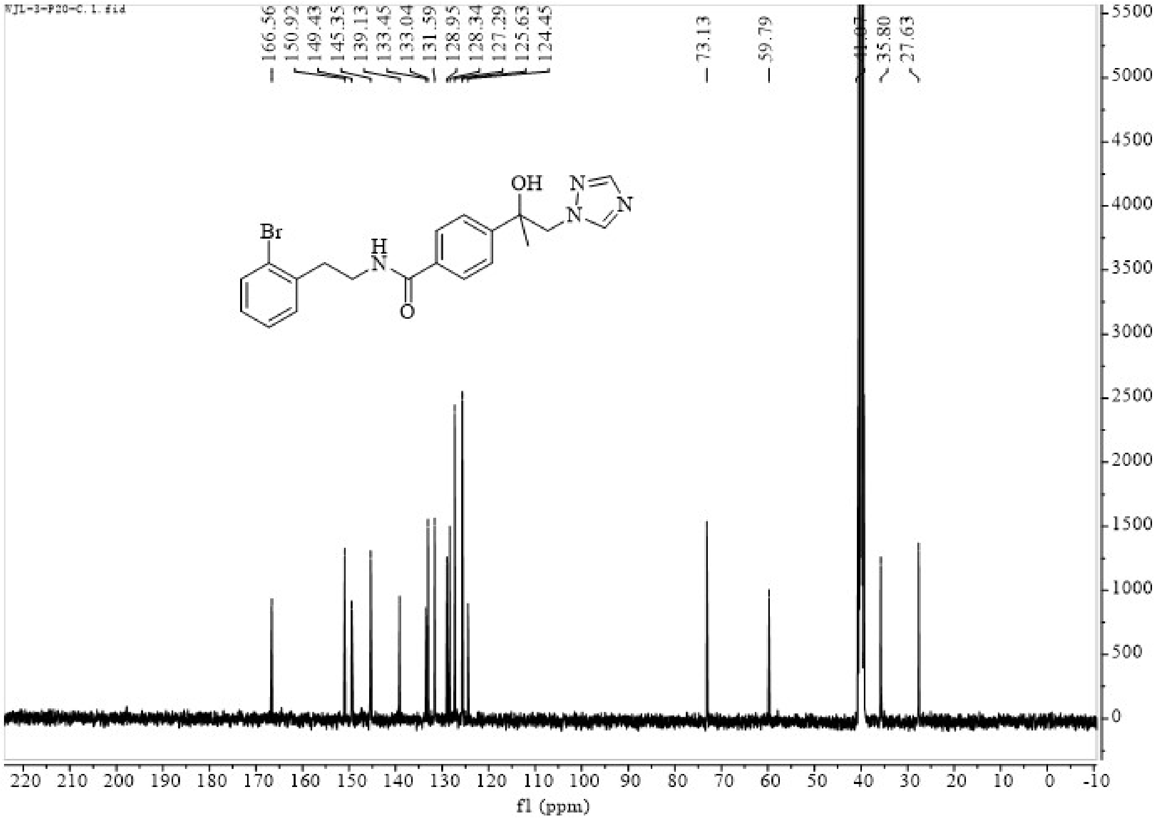

Figure S72. ^{13}C NMR spectrum of **7f**

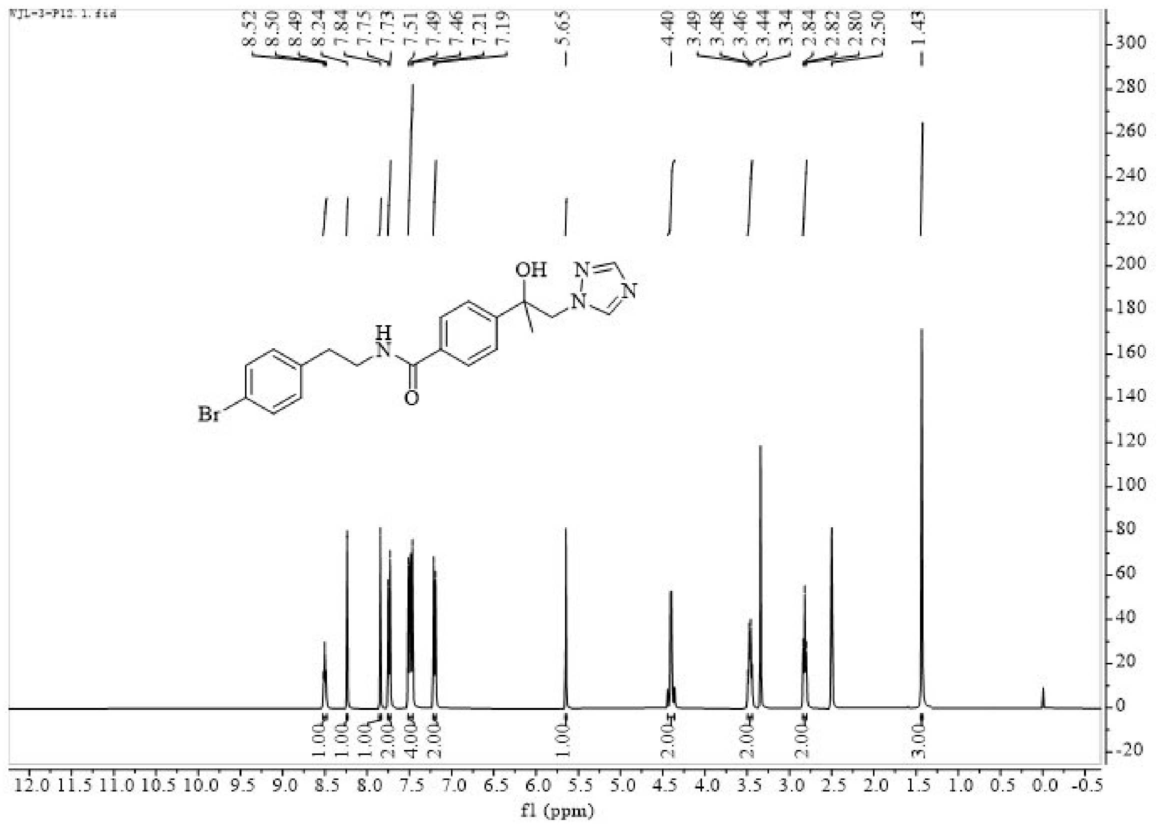

Figure S73. ^{1}H NMR spectrum of **7g**

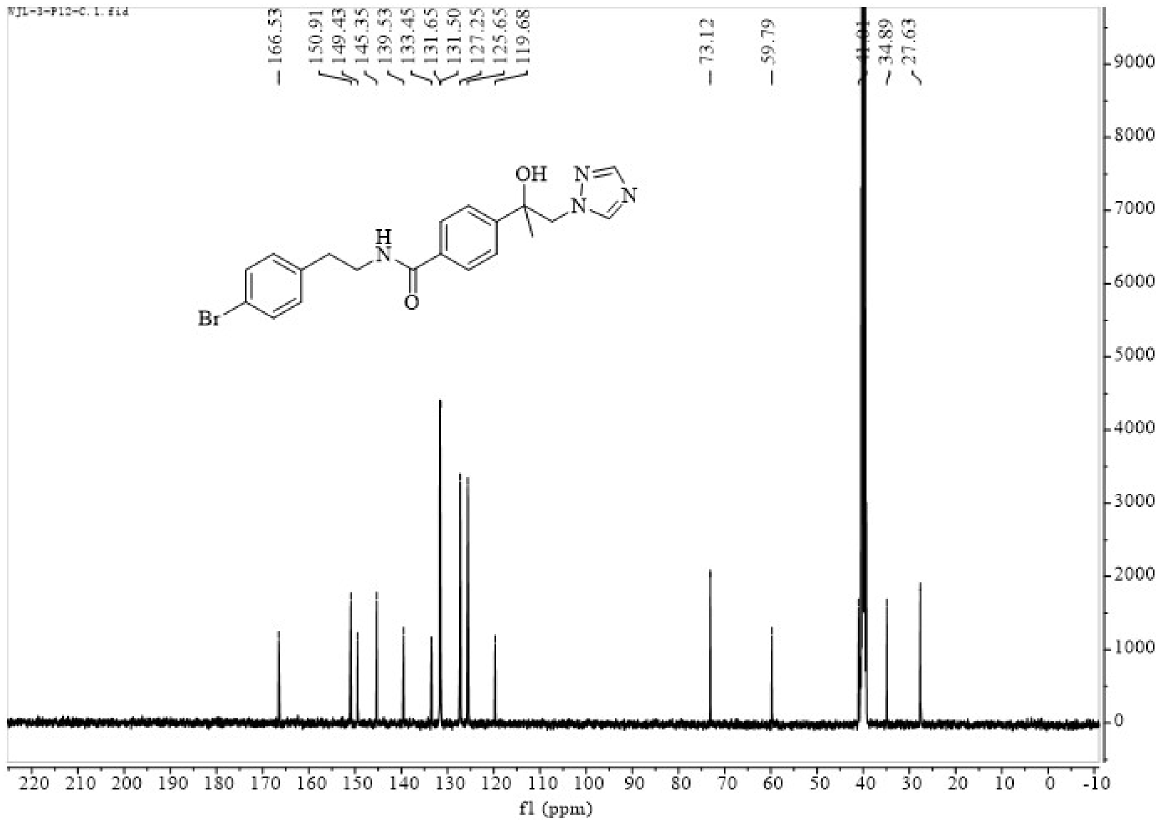

Figure S74. ^{13}C NMR spectrum of **7g**
